# Interpretable Machine Learning in Food Analysis: Closed-Form Chemometric Calibration of Mid- and Near-Infrared Spectra Using Kolmogorov–Arnold Networks

**DOI:** 10.3390/foods15203639

**Published:** 2026-10-13

**Authors:** Abdulhamid Batayhi, Muhammed Özgölet, Hasan Cankurt, Muhammet Arici

**Affiliations:** 1 Department of Mechatronics Engineering, Faculty of Mechanical Engineering, Yildiz Technical University, 34220 Istanbul, Türkiye; abdulhamid.batayhi@std.yildiz.edu.tr; 2 Department of Food Engineering, Faculty of Chemical and Metallurgical Engineering, Yildiz Technical University, 34220 Istanbul, Türkiye; mozgolet@yildiz.edu.tr (M.Ö.); hasan.cankurt@yildiz.edu.tr (H.C.); 3 Department of Nutrition and Dietetics, Faculty of Health Sciences, Biruni University, 34214 Istanbul, Türkiye

**Keywords:** Kolmogorov–Arnold network, interpretable machine learning, closed-form calibration, symbolic regression, FTIR spectroscopy, near-infrared spectroscopy, food adulteration, tahini, chemometrics

## Abstract

A Kolmogorov–Arnold network (KAN) learns a univariate function on every edge, so a trained network can be written as a closed-form equation, unlike a multilayer perceptron (MLP). A KAN was compared with five models, with selection nested within each withheld-lot or withheld-season fold, on tahini adulteration by attenuated total reflectance Fourier-transform infrared (ATR–FTIR) spectroscopy (1554 spectra, released here) and mango dry matter by near-infrared (NIR) spectroscopy (across-season test). No accuracy advantage was found: the KAN’s coefficient of determination (R^2^) was comparable to a parameter-matched MLP’s but never higher on average, and on withheld lots no difference between the leading models was significant. Most of the accuracy lost in converting the network to an equation was due to the conversion procedure; correcting it raised the median external R^2^ of the tahini equation from 0.952 to 0.988. Repeated conversions of one network gave equations scoring −0.76 to 0.96, whereas an elementary edge basis makes the printed equation identical to the network up to rounding. With every lot serving as the blank, detection limits for samples resembling the calibration population were 8.9–21.6% adulterant, and blends at 4–12% were not quantified. The KAN provides an exact, inspectable screening calibration rather than higher accuracy.

## 1. Introduction

Food fraud and the misrepresentation of quality create a standing demand for rapid, non-destructive screening [1]. Vibrational spectroscopy in the mid-infrared (MIR) and near-infrared (NIR) regions is the most widely used approach, because a spectrum records a chemical fingerprint in seconds without reagents [2]. Sesame is a relevant example: it costs several times more than the seed oils and nut pastes with which it can be extended, and attenuated total reflectance Fourier-transform infrared (ATR–FTIR) spectroscopy with chemometrics detects that substitution in cold-pressed sesame oil [3]. Tahini (sesame paste) is defined as a product of sesame alone by the Codex regional standard for tehena [4] and by the Turkish Food Codex [5], and undeclared peanut in products sold as tahini has led to allergen recalls [6]. NIR calibration of dry matter in intact mango underpins commercial maturity grading and is among the most thoroughly characterised quantitative NIR problems in horticulture [7].

Vibrational spectroscopy is also widely used to authenticate edible oils [8] and to monitor lipid oxidation in sunflower kernel paste [9]. One ATR–FTIR study of tahini spiked with peanut or sunflower oil, rather than paste, reported one-class authenticity thresholds of about 10.8% [10]. We found no quantitative calibration of tahini substituted with other pastes over the complete mixing range and with more than one adulterant. Confirmatory methods work at far lower levels: gas chromatography of fatty acids detects oil substitution of a few percent [11], and DNA-based, immunochemical and mass-spectrometric methods detect peanut or sesame at mg/kg levels [12,13,14], three or more orders of magnitude below the detection limits of spectroscopic screening. Spectroscopic screening therefore addresses percent-level substitution only: a negative result cannot exclude trace peanut, which matters to allergic consumers, and a positive result requires confirmation by one of these methods.

Partial least squares (PLS) regression [15], usually preceded by scatter and baseline correction such as the standard normal variate (SNV) transformation [16] and Savitzky–Golay (SG) differentiation [17], remains the standard spectroscopic calibration method because it is transparent: its latent variables and coefficients map back onto wavenumbers, and through them onto chemical bonds, so that each term of a calibration can be related to a chemical assignment. Its principal limitation is that it is linear. More flexible learners, such as support-vector regression (SVR) [18], random forests (RF) [19] and one-dimensional convolutional neural networks (CNNs), can model the non-linear structure that PLS does not capture; CNNs have repeatedly matched or surpassed PLS on NIR calibration [20], including dry matter in mango [21]. This flexibility comes at the cost of transparency: these models expose no functional form, and although post hoc explainers such as SHapley Additive exPlanations (SHAP) [22] attribute a prediction to input features, such an attribution approximates the model rather than constituting it [23]. In this paper, “interpretable” has a deliberately narrow meaning: the calibration is a closed-form equation of elementary functions of its inputs, which a reader can inspect and evaluate [24], a property Lipton calls simulatability [25]. It does not imply that the variables identify the underlying chemistry.

Kolmogorov–Arnold networks (KANs) were proposed as a more interpretable alternative to the multilayer perceptron (MLP) [26]. A KAN learns a univariate function on every edge and sums those functions at its nodes, whereas an MLP applies a fixed activation at each node and learns a single scalar weight on each edge. A trained network can therefore be pruned and each surviving edge replaced by an elementary analytic form, collapsing the model into one closed-form expression; the same mechanism has been used to recover physical relationships from data [27]. Because the standard B-spline edge functions are not themselves elementary, this replacement, here called conversion, is an additional fitting step, and the resulting equation only approximates the network. Chebyshev [28], radial-basis [29] and wavelet [30] edge functions have also been proposed; with an elementary basis no conversion is needed, and the trained network is itself the equation.

KANs have been rapidly adopted in spectroscopy, including food analysis: radial-basis variants for coal calorific value [31], a KAN classifier behind a vision transformer [32], a convolutional–KAN hybrid for functional groups in MIR spectra [33], KAN modules on convolutional or recurrent backbones for Raman diagnostics [34,35,36], and, in food and agriculture, crude fat in maize [37] (known to us only from its title), hyperspectral authentication of dried fish maw [38], small-sample NIR calibration inside a recurrent network [39], and image-based disease and maturity grading [40,41]. We therefore make no claim of priority for applying a KAN to food spectroscopy. In all of these studies, the KAN is a component selected for accuracy within a larger architecture, and in none of the studies we were able to access is the trained calibration reported as a closed-form expression. The distinguishing property of the architecture, its expressibility as a closed-form equation, has therefore not yet been exploited in this literature.

Our companion study evaluated an interpretable KAN on three ATR–FTIR matrices under leakage-free, sample-grouped validation [42]. That study found the KAN competitive with, but not superior to, the baselines, and reported closed-form equations with no loss of accuracy, a result the present study qualifies (Section 3.3). Its claim that the KAN is more parameter-efficient is withdrawn here, because a parameter-matched MLP was comparable in accuracy and had the higher mean coefficient of determination (R^2^) in every configuration compared (Section 3.6). It left three questions open: all three matrices were measured by MIR; none was clearly non-linear; and validation was confined to a fixed pool of five commercial brands, so no external population or season was withheld. The present study was designed to address these questions by evaluating the same KAN against the same five baselines on two contrasting problems. The first is a newly collected ATR–FTIR dataset of tahini adulterated with sunflower and peanut paste over the complete mixing range; as a lipid-in-lipid dilution, it should follow Beer–Lambert mixing and be essentially linear, a regime in which a non-linear model is not expected to outperform a linear calibration. The second is the public mango dry-matter NIR benchmark [7], which is expected to be non-linear, is the canonical dataset on which CNNs outperform PLS [21,43], and supplies the external test that the companion study lacked.

The aim was to determine whether a KAN calibration can be reported as an exact closed-form equation, and at what cost in accuracy and reproducibility. Both calibrations are intended for material resembling the calibration population, and the tahini calibration is intended as a rapid screen for percent-level substitution, not as a confirmatory or regulatory quantitative method; the tahini dataset is released with this paper. The specific objectives were:

1. To compare the KAN with PLS, SVR, RF, an MLP and a CNN, including on withheld production lots and harvest seasons with all model selection repeated inside each training set;
2. To compare the KAN with a parameter-matched MLP and with additive glass-box models;
3. To quantify the accuracy lost when a B-spline KAN is converted to an equation, how much a corrected conversion recovers, and how reproducible the conversion is;
4. To test whether an elementary edge basis removes the conversion step, and whether the printed equation remains identical to its network on withheld lots and seasons;
5. To write the calibration in a few named absorbance bands, determine how many are needed, and test whether the selected tahini bands reflect composition rather than instrumental artefacts;
6. To estimate the limit of detection with each authentic tahini lot serving in turn as the blank.

## 2. Materials and Methods

### 2.1. Tahini Samples and ATR–FTIR Spectral Acquisition

Five authentic tahini (sesame paste) samples, each from a different production lot (T1–T5), were obtained from established suppliers in Istanbul, Türkiye, and the two adulterants—sunflower paste and peanut paste—from a local supermarket. Both are lipid-rich seed or nut pastes like tahini, which makes the substitution difficult to detect spectroscopically. Blends span the complete mixing range, with adulterant fractions from 4% to 100% in 4% steps. Each lot contributes five sunflower and five peanut blends at interleaved levels, so that each of the 25 levels appears exactly once per adulterant across the five lots; together with the five unadulterated lots, this gives 55 physical samples. Blends were prepared gravimetrically (by weight). Each blend was homogenised by hand with a spatula for 2 min and scanned 14 to 17 times, with repositioning on the ATR crystal (ZnSe) between acquisitions. The authentic lots were scanned more often, 150 or 151 times each, to characterise the spectral variability of the authentic reference class against which adulteration is assessed and to balance the authentic and adulterated classes. The dataset comprises 1554 spectra, 754 authentic and 800 from blends, each with 1762 channels at a spacing of about 1.93 cm^−1^.

The levels assigned to each lot were T1: 4, 24, 44, 64 and 84%; T2: 8, 28, 48, 68 and 88%; T3: 12, 32, 52, 72 and 92%; T4: 16, 36, 56, 76 and 96%; and T5: 20, 40, 60, 80 and 100%.

Spectra were recorded on an FTIR spectrometer (Bruker Tensor 27; Bruker Optik GmbH, Ettlingen, Germany) with an ATR accessory, at 4 cm^−1^ resolution over 4000–600 cm^−1^ with 16 co-added scans per spectrum, and exported with SpectraGryph 1.2 (Dr Friedrich Menges, Oberstdorf, Germany).

### 2.2. Mango NIR Dataset

The NIR part of the study uses a public benchmark so that the results can be compared with the published literature. The mango dry-matter dataset of Anderson et al. [7] (Mendeley Data 46htwnp833) comprises 11,691 absorbance spectra of intact fruit over 285–1200 nm at 3 nm spacing, each paired with an oven-dried dry-matter content and a population identifier (112 populations). The populations span four harvest seasons, two growing regions, ten cultivars, two fruit conditions (hard-green and ripened) and a recorded temperature-treatment label; fruit size and skin properties are not recorded. These sources of variation were included in the benchmark by design rather than controlled, and their association with the prediction errors is analysed in Section S18 (the temperature label could not be analysed, because it has a single level in season 4). Following the source study, modelling used the 684–990 nm band (103 channels), and the published partition is used unchanged: seasons 1–3 for calibration and tuning (*n* = 10,243), season 4 for external validation (*n* = 1448). The two partitions share no population (94 and 18 populations, respectively), so the external test requires the calibration to predict a harvest season and populations not seen during calibration. The shift between them is substantial: mean dry matter is 16.18% (standard deviation (SD) 2.41) in the calibration set, compared with 17.02% (SD 2.67) in season 4 [7,21], and ripened fruit make up 61% of season 4 but occur in only one calibration season.

### 2.3. Validation Design, Preprocessing and Leakage Control

**Figure 1:**
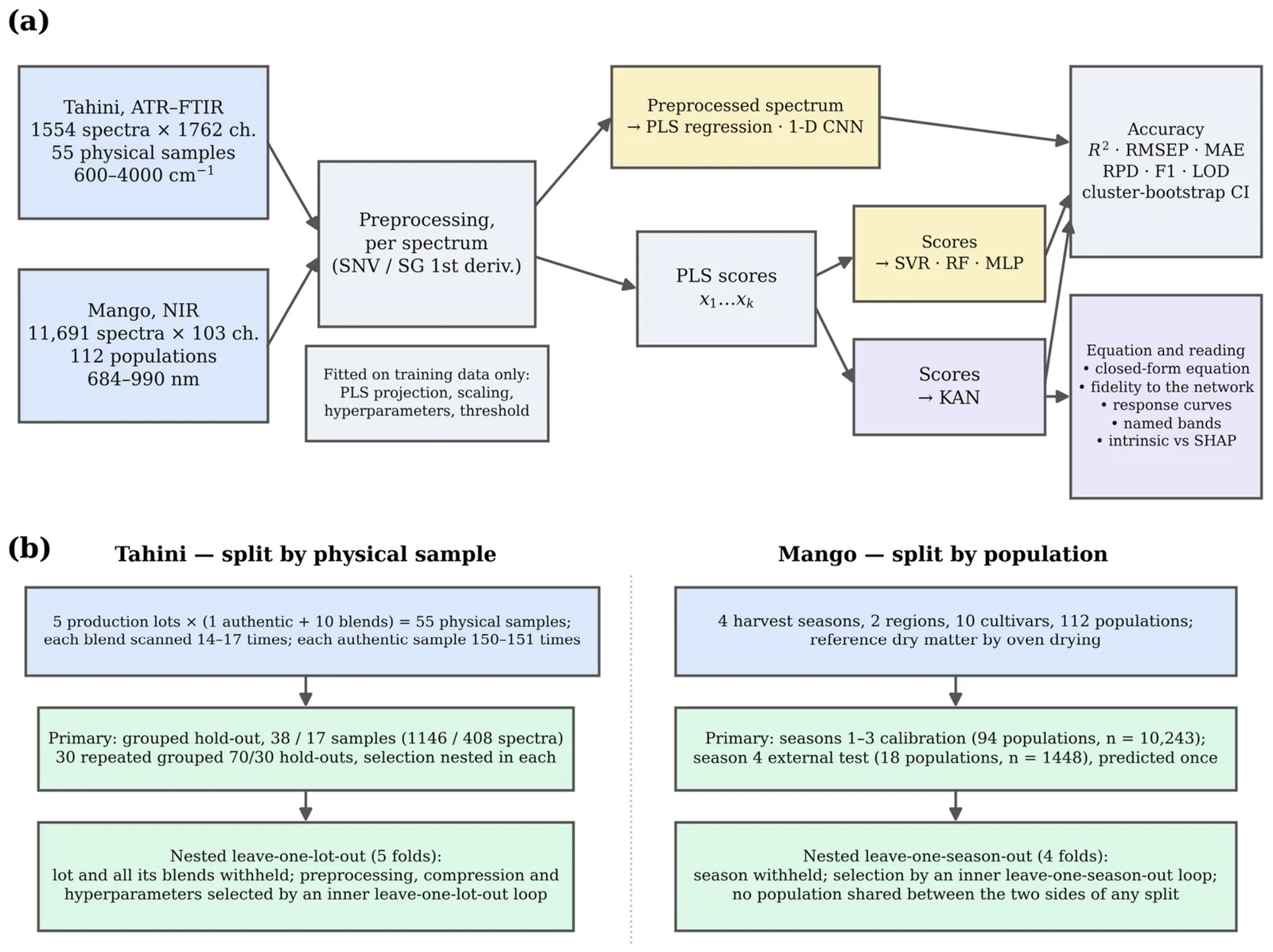
The shared, leakage-free analysis pipeline and the splitting protocol. (**a**) Every fitted transform—the PLS projection, scaling, every hyperparameter and the detection threshold—is learned on the training partition only, and the held-out partition is used only once. In the reruns of Section S3 and the control design of Section S4 the transforms are refitted per split or fold but the hyperparameters are carried over from the primary partition; in the nested leave-one-lot-out and leave-one-season-out validation and the 30 repeated grouped hold-outs, compression and hyperparameters (and, in one tahini analysis, also the preprocessing) are selected by an inner loop within each training set. PLS regression and the CNN read the preprocessed spectrum directly; SVR, RF, the MLP and the KAN read the leading PLS latent scores (Table 1). (**b**) The splitting unit is the physical sample for tahini and the population for mango, so replicate scans of one material never appear on both sides of a split.

For tahini the unit of splitting is the physical sample—one pure lot or one prepared blend—of which there are 55. Replicate scans of one sample are far more similar to one another than to scans of another, so treating them as independent would inflate apparent accuracy. No split, fold or bootstrap resample therefore separates the replicate scans of a sample; for mango, the corresponding unit is the population. Samples are in turn nested within five production lots, and populations within four harvest seasons. The primary tahini partition is a grouped hold-out, approximately 70%/30%: 38 samples (1146 spectra) for training and 17 (408 spectra) for testing. Because a single split can favour or penalise a model by chance, Section S3 repeats the comparison over ten independent grouped hold-outs (Section S23 over thirty, with nested re-tuning) and Section S4 withholds one entire tahini lot or mango season at a time, with all selection nested inside each fold (Figure 1).

The composition of every tahini blend is a three-component vector summing to 100%, and every model predicts all three; error statistics are quoted for the tahini fraction, and Section S2 uses the other two to name the adulterant. Samples below 100% tahini were labelled adulterated for the binary task. Every recorded composition label was checked against the coding of its sample identifier, a check that revealed one inconsistent label in the companion study [42]; no inconsistency was found here, and no recorded value was corrected.

Every quantity estimated from data was estimated on the training partition only: the reference spectrum of multiplicative scatter correction (MSC), when used; the PLS projection; the scaling of each model’s inputs and outputs; every hyperparameter; and the detection threshold. SNV [16] and SG filtering [17] act on each spectrum separately and estimate nothing from other spectra, so they are identical whether applied before or after splitting. Preprocessing was SNV for tahini and, for mango, a first-derivative SG filter (window 11, second-order), chosen from a narrow grid on the training data; Section S6 repeats that selection over a widened grid. Hyperparameters, the PLS projection and the detection threshold were likewise selected inside the training partition of the primary split, by three-fold grouped inner cross-validation maximising the mean inner-fold coefficient of determination (R^2^; averaged over the three composition fractions for tahini), never on its test set. This procedure has exceptions. In the reruns of Section S3, and in the control design of Section S4, preprocessing and the projection are refitted inside each split or fold, but the hyperparameters are not re-selected: every model is refitted at the configuration chosen on the primary training partition, whose samples reappear on the held-out side of those reruns. Those sections therefore measure sensitivity to the split, not to the tuning. The same applies to the three grouped five-fold partitions from which the out-of-fold detection limit of Section 3.2 is computed; its nested counterpart is reported in Section S17. In the nested leave-one-lot-out and leave-one-season-out validation (Section S4) and the thirty repeated hold-outs (Section S23), compression and hyperparameters are instead re-selected by an inner loop within each training set; in one tahini analysis the preprocessing was also selected in that loop (described below).

The second design tests transfer to material from a new source. In leave-one-lot-out validation for tahini, each production lot is withheld in turn together with all ten blends prepared from it (five folds); in leave-one-season-out validation for mango, each harvest season is withheld in turn (four folds). The whole selection is nested inside each fold: an inner leave-one-lot-out or leave-one-season-out loop over the remaining units selects the hyperparameters and, for the MLP and the KAN, the number of PLS scores, by the mean inner-fold R^2^, and the chosen configuration is refitted and applied once to the withheld unit, which therefore plays no part in any selection. For both datasets this was run with the preprocessing of the primary partitions, which isolates the effect of nesting. For tahini it was also run with the preprocessing selected in the same inner loop from five candidates (SNV, MSC, first-derivative SG, SNV followed by first-derivative SG, or none), with SVR and RF then also searching their number of PLS scores; the CNN, by far the most computationally expensive model, was not included in this second run. For comparison, the same folds were also run at the configuration selected on the primary partition, as in Section S4; that configuration was chosen with material from every tahini lot and from three of the four mango seasons present, so it overstates transfer and is reported as a control. Because each lot carries its own set of adulteration levels, every level of both adulterants, including 4, 8 and 12%, and every authentic lot are predicted out of fold exactly once in leave-one-lot-out validation.

The third design addresses the statistical power of the tahini comparison. The six-model comparison was repeated over 30 random grouped 70%/30% hold-outs, with the hyperparameters re-selected inside each training set by the same inner cross-validation, and each model fitted once per split.

Stochastic models (RF, MLP, CNN and KAN) were fitted with three random seeds in the lot and season folds, and a fold’s R^2^ is the mean over seeds.

**Table 1:** Composition, acquisition, model inputs and outputs, and validation designs of the two datasets.

Property	Tahini (This Study)	Mango (Public Benchmark)
Matrix	Tahini (sesame paste)	Intact mango fruit
Technique	ATR–FTIR (mid-infrared)	NIR absorbance
Spectral range modelled	600–4000 cm^−1^ (1762 channels)	684–990 nm (103 channels)
Target	Tahini fraction (%)	Dry-matter content (% *w*/*w*)
Adulterants/factors	Sunflower paste, peanut paste; 4–100% in 4% steps	4 seasons, 2 regions, 10 cultivars, 2 fruit conditions
Spectra	1554 (754 authentic, 800 blends)	11,691
Grouping unit	Physical sample (55)	Population (112)
Replicates per group	14–17 per blend; 150–151 per authentic lot	Variable (per population)
Primary validation	Grouped hold-out, 38/17 samples (1146/408 spectra)	Published across-season external test (season 4, *n* = 1448)
Preprocessing selected	SNV	Savitzky–Golay 1st derivative (window 11, order 2)
Lots or seasons	5 production lots (T1–T5), each blended with both adulterants at its own five levels	4 harvest seasons (94 calibration, 18 test populations)
Off-distribution validation	Nested leave-one-lot-out (5 folds)	Nested leave-one-season-out (4 folds)
Repeated validation	30 random grouped 70/30 hold-outs, selection nested in each	—
Input, PLS regression and CNN	SNV spectrum, 1762 channels (PLS: 12 latent variables)	First-derivative spectrum, 103 channels (PLS: 24 latent variables)
Input, SVR and RF	12 PLS scores	16 PLS scores
Input, MLP and KAN	8 PLS scores	MLP: 16 PLS scores; KAN: 24 (accuracy) or 12 (compact) PLS scores
Model output	Tahini, peanut and sunflower fractions (%), summing to 100	Dry matter (%)
Source	Collected for this study	Anderson et al. [7] (Mendeley 46htwnp833)

### 2.4. Calibration Models

Six calibration models were compared. Two read the preprocessed spectrum directly: PLS regression [15] and a one-dimensional CNN built to the design established for NIR calibration [20]. The other four read the leading PLS latent scores: a radial-basis SVR [18], an RF [19], an MLP and the KAN [26]. This compression is necessary because adjacent channels are almost perfectly correlated; its disadvantage is that the equation is expressed in latent variables, so the loadings are required to interpret it. Section 3.4 addresses this limitation. The inputs, data dimensions and outputs of each model are listed in Table 1.

PLS reads the spectrum because it derives its own latent variables, and the CNN because convolution requires a contiguous, ordered spectral axis whose neighbouring channels describe band shape. A PLS score is the projection of a preprocessed spectrum onto a PLS weight vector fitted on the training set: a weighted sum over all channels, with the weights chosen so that the scores covary with the composition, and with successive scores mutually uncorrelated. The compression also keeps the number of inputs small relative to the number of independent samples and, for the KAN, keeps the number of edge functions, and hence the length of the printed equation, small. It is linear but supervised, and the models that read the scores can be non-linear in them. For mango, three alternative inputs were also compared (Section S19): all 103 channels directly, unsupervised principal-component scores and kernel principal-component scores. The named-band models of Section 2.7 use no compression at all.

For tahini, every model predicts the three composition fractions; negative predictions are set to zero and the three are rescaled to sum to 100%. Accuracy is reported for the tahini fraction, and the larger of the two predicted adulterant fractions identifies the adulterant (Section S2).

Edge functions were cubic B-splines on a five-interval grid, with inputs rescaled to the spline domain [−1, 1] using the training set (test inputs of these B-spline networks were not clipped; the clipping rule of Section 2.7 applies to the elementary-basis networks and their printed equations). For tahini, one hidden layer of width three was used. For mango, the hidden width was included in the search: a two-layer network was selected on accuracy grounds, and the single hidden layer of width three was retained as the variant from which the equation is read, so that the trade-off between accuracy and readability is reported explicitly. Sparsity was encouraged by an L1-plus-entropy penalty on the learned activations, with weight λ = 1 × 10^−3^, followed by pruning. Optimiser settings, every step budget, and the six additional baselines of Section 3.6 are specified in Section S13; Section S5 measures how much each choice matters.

Tuning budgets were made comparable, because a comparison against an untuned baseline would favour the tuned model. Every model was searched over a comparable number of configurations by inner cross-validation confined to the training partition, and the two deep baselines over several architectures rather than one. On the primary partitions, SVR and RF used a fixed 12 (tahini) or 16 (mango) PLS scores, whereas the MLP and KAN searched their number; the nested design with preprocessing selection removes this asymmetry. The 38 tahini training samples are fewer than the trainable parameters of the neural models (Table 2 and Table 3). Overfitting was limited by grouped inner selection, fixed training budgets, sparsity and pruning (KAN), and batch normalisation, dropout and weight decay (MLP); a learning curve over the number of training samples tests the effect of this small sample size (Section S20). An MLP was then built to match the KAN’s parameter budget as closely as possible (684 against 689 on tahini; 8996 against 8989 and 820 against 817 on mango). Because the outcome depends on both the training procedure and the configuration of each architecture, these two factors were crossed rather than fixed. In addition, because the published recipes give the MLP 3600 (tahini) and 32,200 (mango) gradient updates against the KAN’s 300, each architecture was also trained at the other’s budget (Sections S7 and S21).

### 2.5. Performance Metrics and Statistical Analysis

Accuracy is summarised by R^2^, the root-mean-square error of prediction (RMSEP), the mean absolute error (MAE) and the ratio of performance to deviation (RPD). For tahini we additionally score the binary decision by the F1 score (the harmonic mean of precision and recall) and accuracy, with the threshold on the predicted tahini fraction fixed inside the training cross-validation (chosen to maximise the cross-validated F1 of PLS and then applied to every model). In leave-one-lot-out validation the threshold is re-chosen inside each training set, which gives the false-positive rate on each withheld authentic lot. The limits of detection (LOD) and quantification (LOQ) follow the calibration-slope convention, LOD = 3.3σ/S and LOQ = 10σ/S, in which σ is the SD of the predicted adulterant fraction on authentic spectra and S is the slope of the regression of predicted on nominal composition.

The reported LOD is out of fold, with each authentic lot serving as the blank while it is withheld: predictions are pooled over three grouped five-fold partitions of all 55 samples (Section 3.2) and, separately, over the nested leave-one-lot-out folds. The LOD from the single authentic lot in the test partition is given for comparison only.

All 95% intervals come from a cluster bootstrap (1000 draws unless stated otherwise) whose resampling unit is the physical sample (17 test samples) or the population (18 test populations), never the individual scan; treating replicate scans as exchangeable would give intervals several times too narrow. For the detection limit both σ and the slope are re-estimated inside every draw (the five lots for σ and the 55 samples for S; 2000 draws), and where two limits are compared the difference is read off the same draw for both models (Table S15). In addition, a two-stage bootstrap [44] resamples lots and then samples within lots for R^2^, the LOD and paired model differences (Section S17). With five lots and four seasons, the upper resampling level is coarse; lot and season results are therefore also reported fold by fold.

Pairwise comparisons raise a second dependence problem, because cross-validation training sets overlap and a naive paired *t*-test on fold scores understates the variance of their differences. We use the corrected resampled *t*-test of Nadeau and Bengio [45] and control the family-wise error rate within each family by Holm–Bonferroni [46]; Section S12 gives the variance correction each design implies and tabulates every test behind Section 3.3, Section 3.4, Section 3.5 and Section 3.6 except those of the parameter-matched comparison, for which a simple paired *t*-test over seeds is reported alongside the corrected test, both Holm-adjusted over the twelve comparisons (Section S24). The combination is deliberately conservative, and with three seeds or five folds it has limited power, so a non-significant difference is interpreted as an absence of evidence rather than as evidence of equivalence. For the headline comparison, the corrected test is applied to 30 repeated grouped hold-outs with nested re-tuning, the design for which it was derived: the variance of the mean paired difference is multiplied by (1/J + n_test_/n_train_) with J = 30, on J − 1 degrees of freedom. On the same corrected variance, equivalence was tested by two one-sided tests (TOST) [47] at margins of ±0.01 and ±0.02 R^2^, so that a pair can be classed as different, equivalent within a margin, or undetermined. A non-significant difference without equivalence support is reported as a descriptive ranking, not as a tie.

Repeated random seeds on a fixed partition measure only training randomness; they cannot increase the information about new material, which is limited by the five lots and four seasons. Repeated random splits do not remove this limit either: the corrected test inflates the variance by a term for the overlap between training sets that does not shrink as the number of splits grows, so the 30 repeated hold-outs of Section S23 are used to estimate the spread of each model and to test equivalence, not to create significance. Both the symbolic conversion and early stopping can turn very small numerical differences, for example in the order of floating-point summation, into markedly different results. Identical runs were therefore repeated within one process, at different thread counts and across seeds, and the three spreads are reported separately (Sections S8 and S9). Where a single equation is quoted, the seed that produced it is named and its distribution given alongside.

### 2.6. Extracting the Closed-Form Equation

In the pruned network, each surviving edge is replaced by the best-fitting member of a small library of elementary functions—identity, square, cube, exponential, logarithm, square root, hyperbolic tangent, sine and reciprocal—so that the whole model collapses into one expression. Two properties of the library’s default implementation account for most of the accuracy lost in this step; both are corrected here, and the published default is reported alongside so that the effect of the correction is visible (Section 3.3). Each edge function is fitted to its elementary form using the whole calibration set, rather than the most recently drawn mini-batch; and the converted network is refitted, for 200 further steps with the sparsity penalty off, so that the parameters retained by the substituted functions are fitted jointly rather than edge by edge. The default protocol (P0), the whole-set fit alone (P1), the refit alone (P2) and both (P3) were compared by grouped inner cross-validation, and P0 and P3 were also compared at ten seeds on each external test set (Section 3.3).

Because a conversion of this kind is not reproducible from run to run, no equation is reported without its distribution (Section S9). Alongside accuracy we report fidelity: the R^2^ of the printed equation against the predictions of the network it came from, which separates “the equation is a poor calibration” from “the equation is a poor copy of a good calibration”. Expression length is reported as the number of terms, counting additive components at every level of nesting; thus, a sine of a linear combination of five variables contributes six terms rather than one.

### 2.7. Named Bands and Exact Edge Bases

Two further analyses address the limits of the latent-variable equation. First, an equation in PLS latent variables still requires the loading matrix to evaluate, so the model is simulatable but not decomposable in Lipton’s sense [25]. We therefore also fit the linear controls, the additive glass-boxes and the KAN on a small set of individually selected channels, so that the expression reads directly in named wavenumbers. Channels are ranked inside each training fold by PLS variable importance in projection (VIP), subject to a minimum separation (15 cm^−1^ for tahini, about eight channels, and 9 nm for mango), and their number is selected per model family by grouped inner cross-validation; a ranking by mutual information was also offered to this inner search and was selected for no model. A chemically anchored variant, with the channels fixed in advance at tabulated band positions [48,49,50], is reported alongside, and ordinary least squares (OLS) on the same channels serves as the control for determining whether the non-linearity contributes to prediction. The tahini variables are SNV-corrected absorbances, so evaluating the equation requires the recorded spectrum for the SNV step, but no loading matrix. Whether the number of channels is sufficient, and whether selecting single channels is sensitive to band overlap, were tested with OLS on 2 to 40 channels, as single channels and as ±7.5 cm^−1^ band averages (Section S15).

Second, a cubic B-spline is not an elementary function, so replacing it always introduces an approximation. We therefore also train KAN layers whose edge functions are expanded in a basis that is *already* elementary—Chebyshev polynomials, a truncated Fourier series, Gaussian radial-basis functions or raw powers—in which case printing the trained network is a change of notation rather than a fit. Substituting the spline basis is not in itself new: Chebyshev [28], radial-basis [29] and wavelet [30] edge functions have all been proposed as alternatives to it. This study tests a consequence that, to our knowledge, has not previously been evaluated: an already-elementary basis removes the conversion step altogether, so that the printed expression and the trained network are the same object. Every elementary basis is defined on [−1, 1]: inputs are min–max scaled to this interval on the training partition, and any new value outside the training range is clipped to the nearest bound before the network or the printed equation is evaluated, so that no edge function is evaluated outside the interval on which it was fitted: a polynomial term cannot diverge, nor a periodic Fourier term repeat itself, under extrapolation. A clipped value marks a sample outside the calibration domain, whose prediction should be treated as an extrapolation (Section S25). We refer to the conversion of a B-spline network as the converted route, and to training in an elementary basis as the exact route. A wavelet basis was not among those trained here. Fidelity is measured, not assumed: the printed expression is compiled and evaluated on the held-out rows. Fidelity was also measured on the withheld-lot and withheld-season folds (Section 3.5), where both routes were scored on the primary output alone, without the sum-to-100% rescaling, because a printed equation predicts a single output.

### 2.8. Chemical Reading, Artefact Controls and Software

Each learned univariate function is drawn on the same axes as the straight line a linear model would have fitted for that variable, so that any departure indicates where non-linearity is used. The loading vectors of the variables in the equation are read against tabulated band assignments [48,49,50]; channels outside all tabulated bands are reported as unassigned rather than assigned by proximity (Section S10). The claim that the network intrinsically provides the information that a black-box model yields only through an external explanation method was tested by comparison with permutation importance and SHAP [22], on both the latent and the wavenumber axes (Section S11).

Whether the selected tahini channels reflect composition rather than instrument drift, baseline differences, atmospheric water vapour or noise was tested directly on the released spectra (Section S14): the dose–response of each channel along each dilution series, its replication within each production lot, its co-variation with a per-scan water-vapour index, the composition effect after adjustment for water vapour and lot, the frequency with which each channel is selected under shuffled labels and across leave-one-lot-out training sets, and the accuracy lost when the vapour-affected channels are removed.

Analyses used Python 3.13 with scikit-learn [51], PyTorch 2.10.0 [52], pykan 0.2.8, SymPy and the SHAP package [22]. The additional analyses used Python 3.11 with scikit-learn 1.8.0, PyTorch 2.10.0, pykan 0.2.8, NumPy 2.4.3 and statsmodels 0.15.0, and reproduce the benchmark of Table 2 exactly for PLS, SVR, RF and the KAN and to within 0.004 R^2^ for the MLP and CNN. Every result below can be regenerated from the released code with the seeds it records, within the run-to-run variation that is itself reported for the symbolic conversion and for early-stopped training (Section 3.3, Sections S8 and S9). In addition, the initial weights of the first elementary-basis network fitted in each run, including the network of Equation (3), were drawn before the seed was set; such a network is therefore reproduced only to within the variation caused by its initialisation (for Equation (3), external R^2^ 0.9834–0.9846 over five initialisations, against 0.9841 published; Section S25). A generative artificial intelligence (AI) assistant was used to write and check analysis code and for language editing (see Acknowledgments).

## 3. Results and Discussion

### 3.1. Spectral Characteristics and Chemistry

**Figure 2:**
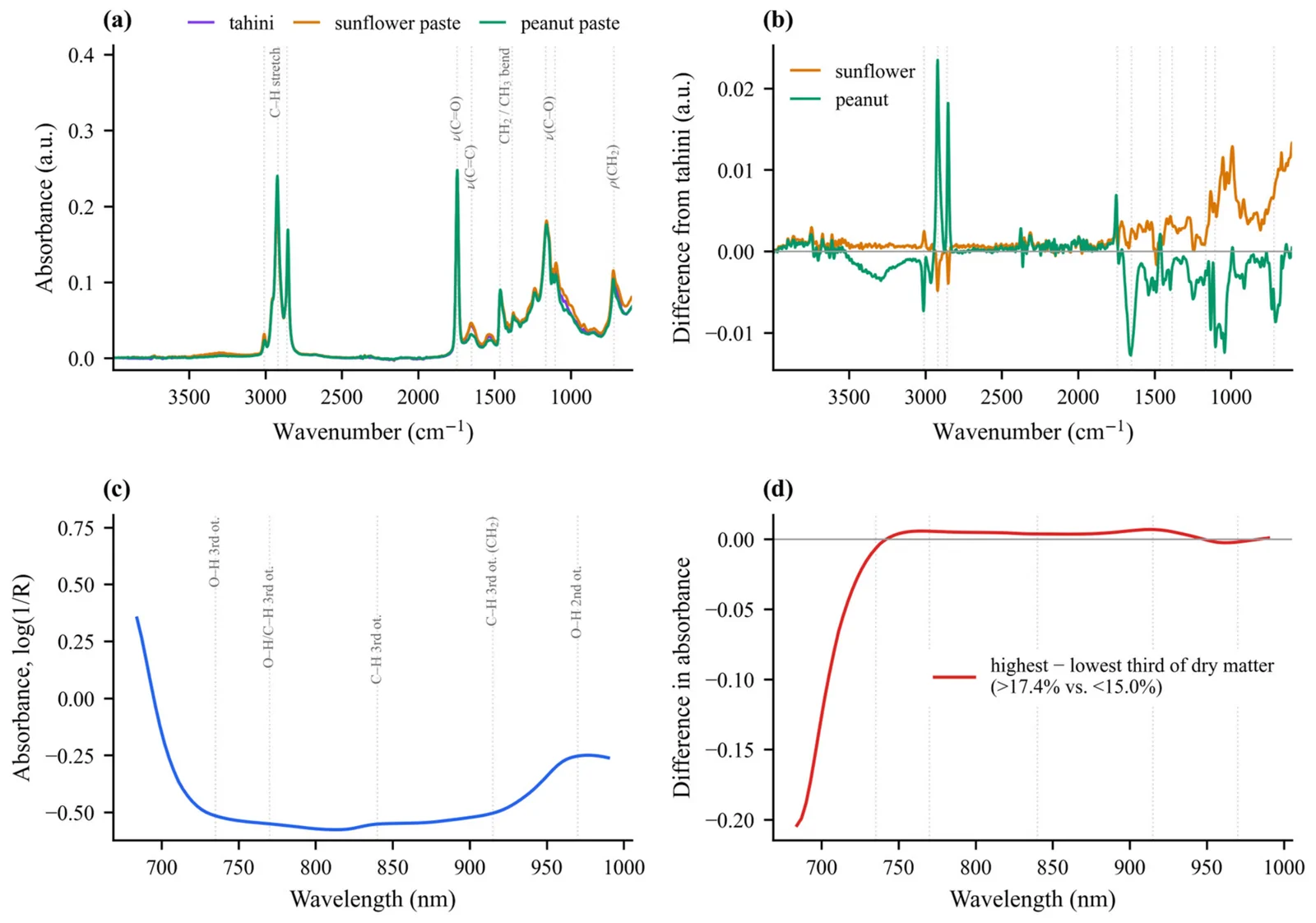
Mean spectra and difference spectra of the two datasets. (**a**) Mean ATR–FTIR spectra of authentic tahini, sunflower paste and peanut paste; (**b**) mean difference spectra of each adulterant relative to tahini; (**c**) mean NIR spectrum of intact mango fruit (all 11,691 spectra); (**d**) mean difference between the highest and lowest thirds of dry-matter content. The vertical scales of the mean and difference panels differ.

The tahini spectra (Figure 2a) carry the triglyceride signature common to all three pastes: the *cis* = C–H stretch near 3010 cm^−1^, asymmetric and symmetric CH_2_ stretching at about 2920 and 2851 cm^−1^, the ester carbonyl near 1745 cm^−1^, CH_2_ scissoring near 1465 cm^−1^, the two C–O stretching bands between 1090 and 1180 cm^−1^, and CH_2_ rocking near 720 cm^−1^ [48,53]. The spectra of the three pure components are almost superimposed, and this constitutes the analytical challenge: adulteration perturbs the intensities of shared bands rather than introducing new ones.

The difference spectra (Figure 2b) show where the usable information lies. Sunflower and peanut paste depart from tahini in opposite directions over much of the fingerprint region, and the sense of that departure reverses in the C–H stretching region. Below about 1200 cm^−1^, sunflower paste absorbs more strongly than tahini at all 311 channels in that region and peanut paste less strongly at 95.5% of them; at the two dominant CH_2_ stretching bands, by contrast, peanut paste absorbs more strongly, by the largest margin in either difference spectrum (+0.0235 at 2920 cm^−1^). The region of strongest absorption is not where the three pastes differ: the tallest band is the ester carbonyl at 1744 cm^−1^, whose differences are among the smallest in the panel. The two adulterants are therefore not dilutions of a common “non-tahini” spectrum but chemically distinguishable perturbations. Because each adulterant is a single commercial product, this conclusion applies only to these materials. This distinction between the adulterants is exploited in Section S2, where the same calibration is used to identify the adulterant. The magnitude of these differences is of order 0.01 absorbance units against a 0.25-unit peak.

The mango spectra (Figure 2c) are dominated by the short-wave NIR envelope of intact fruit, and the difference between the highest and lowest thirds of dry matter (Figure 2d) is again small and structured, with a secondary feature where O–H and C–H overtones of water, sugars and starch overlap [49]. Because water and dry matter are complementary fractions, the dry-matter signal is carried indirectly by the water bands on a scattering background. This indirect relation may introduce non-linearity; this possibility was not tested here, but it was the reason for choosing mango as the contrast to the near-linear tahini matrix (Table 1). Fruit size and skin are not recorded, so their contribution could not be examined. In the present comparison, the KAN differed significantly only from RF on mango, not from PLS (Section 3.2). The benchmark includes, rather than controls, variation in fruit condition, cultivar, temperature and harvest season; the dependence of prediction errors on these factors is reported in Section S18.

### 3.2. Benchmarks, Transfer to New Lots and Seasons, and Detection

**Figure 3:**
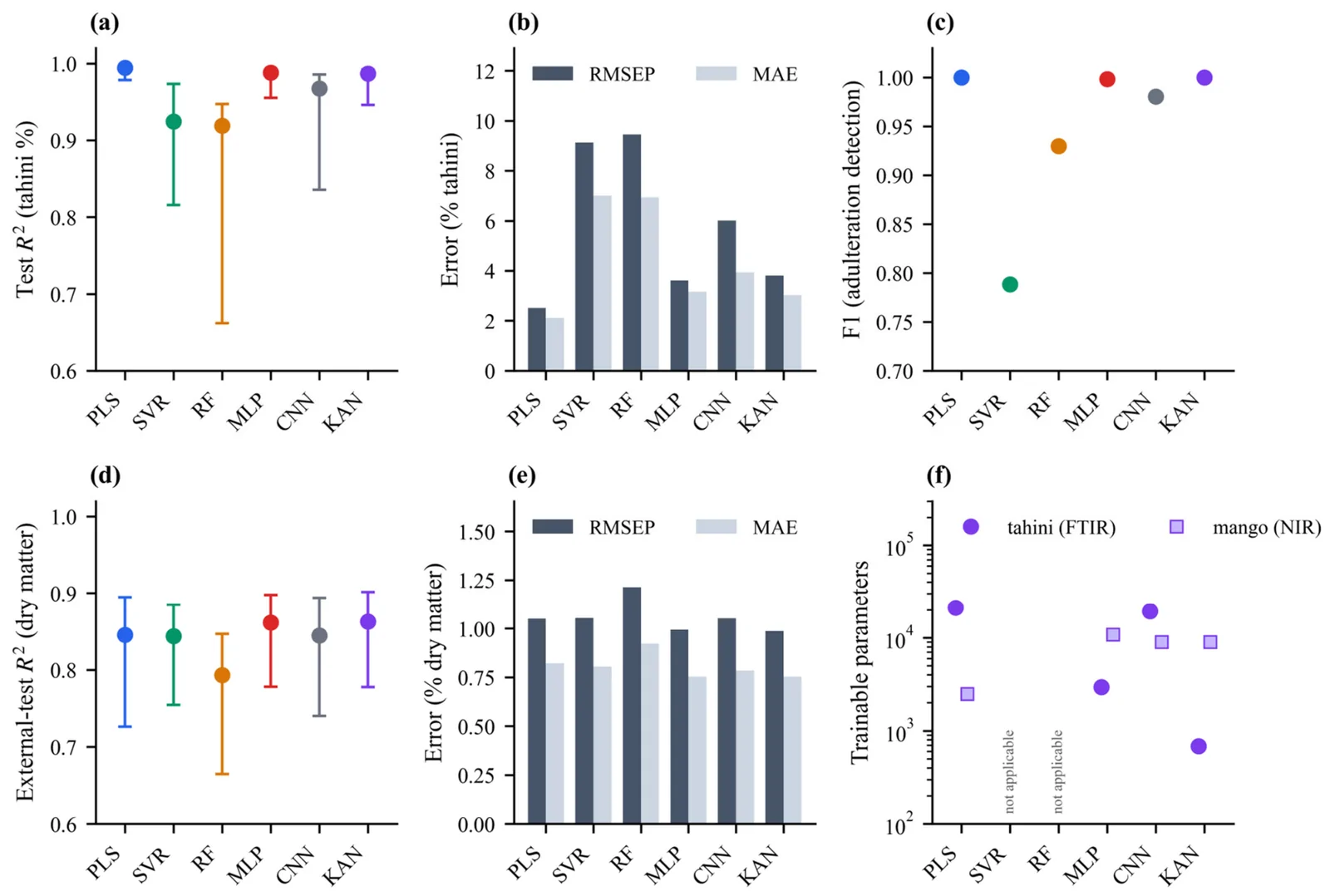
Benchmark comparison of the six calibration models. (**a**–**c**) Tahini: Coefficient of determination, errors of prediction (RMSEP and MAE) and detection F1 on the group-independent test set; (**d**,**e**) mango: coefficient of determination and errors of prediction (RMSEP and MAE) on the across-season external test set; (**f**) trainable parameters, on a logarithmic scale (not applicable to SVR and RF). In (**a**,**d**), points are estimates and error bars cluster-bootstrap 95% intervals.

Tahini: A near-linear task on which PLS ranked highest. On the group-independent test set, the six models ranked, by point estimate, in the order PLS, the MLP, the KAN, the CNN, SVR and RF (Table 2, Figure 3a–c; per-spectrum predictions in Section S1); the intervals of PLS, the MLP and the KAN overlapped throughout, and those of SVR and RF were markedly wider. The KAN reached R^2^ = 0.987 against 0.988 for the MLP and 0.994 for PLS, and in the corrected tests on the cross-validation folds of the training partition, none of the five differences from the KAN reached significance after adjustment (smallest adjusted *p* = 0.36, against PLS; Section S12). Over 30 repeated grouped hold-outs with the selection repeated inside each, the mean R^2^ was 0.990 for PLS, 0.984 for the MLP, 0.958 for the CNN, 0.956 for the KAN, 0.884 for RF and 0.859 for SVR. The KAN therefore ranked fourth, below the CNN, and PLS had a higher R^2^ than the KAN in all 30 splits; after correction for the overlap between training sets, however, no pair of models differed significantly (smallest adjusted *p* = 0.26), and no pair was shown to be equivalent within ±0.01 or ±0.02 R^2^ (Section S23).

Detection saturated at F1 = 1.00 for all three leading models, because the test partition contains no blend below 16% adulterant and a single authentic lot; authentic lots withheld in leave-one-lot-out validation did produce false positives (below). The same calibration identifies the adulterant correctly in every adulterated spectrum for every model except RF (Section S2). Because each production lot carries its own adulteration levels, leave-one-lot-out validation predicts every level of both adulterants out of fold, including 4, 8 and 12% (Section S16). With the selection nested inside each fold, the adulterant was still identified correctly in 80% (KAN), 86% (PLS) and 96% (MLP) of the spectra of blends at 4–12%, but these levels lie below or near the detection limits (below), and their predicted adulterant fractions (for example, 6–19% for the 4% blends with PLS, the MLP and the KAN) do not quantify them. At these levels, a positive result cannot be distinguished from the false positives produced by authentic tahini from an unseen lot (below). The method therefore supports identification of the adulterant in material above the detection limit, and not detection or quantification of low-level adulteration.

Mango: Point estimates on an unseen harvest season. On the published across-season external test (season 4, whose 18 populations were absent from training), the KAN attained the highest point estimate of the six, R^2^ = 0.863 against 0.861 for the MLP, with the CNN, PLS and SVR clustered near 0.845 and RF last (Table 3, Figure 3d,e). The leading position is not stable: rerunning the released code selected a different MLP configuration from a near-tie in the inner cross-validation (mean R^2^ 0.866 against 0.864 for the published configuration), and that configuration reached 0.871 on the external test, above the KAN (Section S8). This difference lies well within the intervals, so the leading models cannot be distinguished; in the corrected tests on the cross-validation folds of the training partition, the KAN differed significantly only from RF (adjusted *p* = 0.04; Section S12), and the ordering of the other five models is therefore a ranking of point estimates. This ranking is subject to one qualification: the six models shared a single training protocol, which makes them comparable but does not ensure that each is individually optimised. The parameter-matched MLP of Section S7 reached 0.897 on the same external test at a matched parameter count; the KAN’s leading estimate therefore holds only *under this protocol* and does not indicate the best performance an MLP can achieve.

Transfer to new production lots and harvest seasons. When each tahini production lot was withheld in turn, with the compression and hyperparameters selected inside each fold and the preprocessing fixed to that of the primary partition, the mean R^2^ over the five lots was 0.968 for the KAN (worst lot 0.948), 0.959 for the MLP (0.896), 0.950 for PLS (0.837), 0.941 for the CNN (0.857), 0.865 for RF (0.590) and 0.786 for SVR (0.307) (Section S4, Table S3). In the control design (Section 2.3), in which the configuration selected on the primary partition, and therefore with samples of every lot, was carried into each fold, the corresponding values were 0.940 (0.841) for the KAN and 0.975 (0.952) for PLS: nesting the selection raised the KAN’s worst-lot R^2^ by about 0.1 and lowered that of PLS by about 0.1. With five lots, these differences between models were not significant (lot-nested bootstrap, adjusted *p* = 1.00 for the KAN against PLS, the MLP and the CNN; Section S17), so the ordering is a ranking of point estimates. With a harvest season withheld and the selection nested in the same way, the mean R^2^ over the four seasons was 0.829 for the CNN (worst season 0.804), 0.827 for the MLP (0.752), 0.805 for PLS (0.719), 0.803 for the KAN (0.752), 0.779 for RF (0.742) and 0.772 for SVR (0.609). These season results were not tested and are a ranking of point estimates; nesting raised the KAN’s worst-season R^2^ from 0.640 to 0.752. When the preprocessing was also selected inside each tahini fold, the KAN reached a mean of 0.976 (worst lot 0.953), SVR and RF rose to 0.964 and 0.966, respectively, and PLS did not improve (0.948; Section S4). Every model lost accuracy on a withheld lot or season relative to the grouped hold-out, and five lots and four seasons cannot establish that any of these calibrations transfers to new production. We therefore present them as screening calibrations for material resembling the calibration population.

Limit of detection. Computed from pooled out-of-fold predictions, with each authentic lot serving as the blank while it is withheld, the detection limit is 8.93% adulterant for PLS, 10.32% for the MLP, 13.11% for RF, 14.27% for the KAN, 14.88% for the CNN and 21.59% for SVR (Table 4, Section S13), a ranking of point estimates (below). Estimated from the single authentic lot in the test partition, the limits were 3.3–3.5% for the three leading models and 19.6% and 22.4% for SVR and RF (Table 4). This estimate is optimistic and is given for comparison only; it also has a structural limitation that its intervals do not reflect: exactly one of the five authentic lots falls on the test side of the split, so the blank dispersion is the scatter of a single lot. Pooled over three grouped five-fold partitions of all 55 samples, so that each lot serves as a blank while it is held out, the limits roughly triple and the ordering changes: for the four accurate models the pooled limits are 2.4 to 4.1 times higher, while the limit for SVR changes little and that for RF improves (Table 4). A bootstrap that resamples production lots and then samples within lots gives the same conclusion with wider intervals (PLS 3.3–11.4%, KAN 2.3–19.6%; Section S17). For accuracy, however, the same bootstrap separates the leading models: the R^2^ of PLS was significantly higher than those of the KAN (+0.017, 95% interval 0.005–0.034, adjusted *p* < 0.001), the CNN and RF (Table S20). In the nested leave-one-lot-out validation, the out-of-fold detection limit was 10.3% for the KAN, 17.5% for the MLP and 19.5% for PLS, again without a significant difference between these three models (Sections S16 and S17).

The increase does not reflect a loss of accuracy: out of fold, five of the six models met or exceeded their cross-validated R^2^. Authentic tahini varies more between lots than within a single lot, and a blank drawn from one lot cannot capture this variation. Wherever a calibration is accurate enough for the blank scatter to be dominated by lot-to-lot variation rather than by its own prediction error, a limit derived from a single blank population is therefore optimistic. That condition holds for the four accurate models and not for the two weakest, and the effect was largest for the KAN. No ranking is drawn from the new ordering either: of the fifteen pairs tested, five differ after adjustment and every one involves SVR (Table S15); under the bootstrap that resamples lots and then samples within them, four pairs differ, all of them SVR against a model other than RF (Section S17). The PLS–KAN pair is not among them under either test, and no pair among PLS, the MLP, the CNN, RF and the KAN differs. The pooled estimate therefore changes the magnitude of the limits, but the change in their ordering is not significant; the relevant screening limits are approximately 9% for PLS and 14% for the KAN. Averaged over all five authentic lots out of fold, every model predicted some adulterant in authentic tahini: from 3.1 percentage points for PLS and 4.3 for the KAN to 11.3 for SVR (Section S16, Table S19); with the selection nested inside each withheld lot, the figures were 6.9, 4.3 and 15.6 (Section S16). The bias is not uniform across lots. When a lot was withheld entirely, the mean prediction for authentic lots T1 and T5 fell below the threshold fixed in the training folds for PLS and the MLP (92.7–100% of their spectra flagged), as did that for lot T5 with the KAN (72%), lot T2 with the CNN, lots T1 and T4 with RF, and four of the five lots with SVR (Section S16); the false-positive rate of every model on each withheld lot is given in Table 5. The false positives therefore arise mainly from authentic lots that differ from the calibration lots, and reflect the same lot-to-lot variation that raises the out-of-fold detection limit. These detection limits and false-positive rates apply only to samples similar to the calibration population, that is, authentic tahini resembling the five calibration lots, adulterated with sunflower or peanut paste and measured on the same instrument; they cannot be transferred to other products, adulterants or instruments without recalibration. In practice, a positive screen must be confirmed by a targeted method (Section 3.7), and the authentic reference set must include the range of lots the method will meet; no regulatory threshold for tahini adulteration exists against which a screening result could be judged, because tahini is defined as a product of sesame alone [4,5].

### 3.3. From Network to Equation: Corrected Conversion and Exact Edge Bases

**Figure 4:**
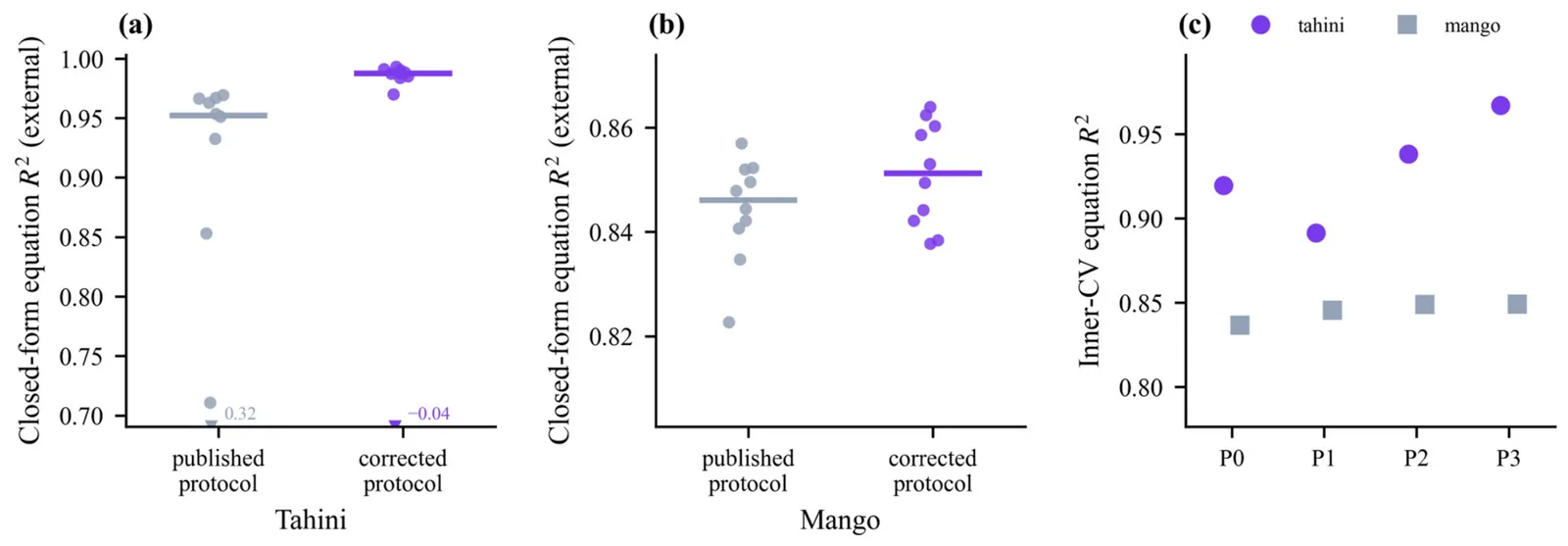
The symbolic-extraction protocol. (**a**,**b**) External-test R^2^ of the converted-route equation at ten random seeds, under the published protocol (P0) and the corrected one (P3), for tahini and mango; horizontal bars are medians, and a run below the plotted range is drawn as a triangle at the lower edge with its value printed. (**c**) Mean equation R^2^ over the grouped inner cross-validation for all four protocols. P1 applies the whole-set fit alone, P2 the post-conversion refit alone, P3 both. Each point is a mean over fifteen runs except P2 on tahini, which is over the thirteen that returned an equation. No corrected test was applied between protocols.

The property that distinguishes this architecture is that a trained network can be converted into one closed-form expression. In one run on tahini, the library’s default conversion reduced R^2^ from 0.983 for the spline network to 0.948. Two causes were identified. First, the conversion fits each substituted elementary form to the activations of the network’s most recent forward pass, a single randomly drawn batch of 128 spectra, so the outcome depends on the state of the random number generator. Second, the converted network is not refitted: each substituted function retains four trainable parameters that the documented workflow fits jointly after conversion, and the published pipeline stopped before that step. We compared four protocols, with all selection performed inside the training partition and the external test set used once: Figure 4c reports inner cross-validation over fifteen runs, and Table 6 reports the published (P0) and corrected (P3) protocols on the external test set at ten seeds. The protocols were not compared by a corrected test.

Both corrections together gave the highest mean inner cross-validation R^2^ of the four protocols, although neither correction alone reached it. On the tahini inner cross-validation the published protocol gave R^2^ 0.920 ± 0.054 against 0.967 ± 0.024 for the corrected one; the refit alone reached 0.938 (over the thirteen of fifteen runs that returned an equation) and the whole-set fit alone (P1) 0.891, *below* the published protocol. On tahini, the whole-set fit therefore improved the result only in combination with the refit; this interaction was not tested and did not reproduce on mango, where it is therefore not claimed. On the external test, the correction brought the equation to the accuracy of its source network: the median external R^2^ rose from 0.952 to 0.988 on tahini and from 0.846 to 0.851 on mango, against network medians of 0.977 and 0.821, respectively. The median fidelity changed from 0.952 to 0.985 on tahini and from 0.972 to 0.969 on mango. The means differed little (tahini: 0.859 ± 0.205 under the published protocol and 0.884 ± 0.324 under the corrected one, whose worst run scored −0.038); on mango the mean shift (0.844 to 0.851) is smaller than the seed SD (0.010) and is descriptive. On both matrices the median equation is *better* than its network (tahini 0.988 against 0.977, mango 0.851 against 0.821). The refit is run with the sparsity penalty off on an already-pruned model and may act as a mild regulariser. The difference between equation and network was not tested, and no equation–network comparison in Table S14 is significant.

Equations (1) and (2) are the converted-route equations this procedure produces, each a single draw from the distribution above rather than a best case, with its seed named as Section 2.5 requires; they are not the calibrations we recommend reporting (see below). Equation (1) is the tahini equation at seed 7: external R^2^ 0.9889 against 0.9735 for the spline network it came from, at a fidelity of 0.9846, in 21 terms over seven of the eight latent variables. Equation (2) is the mango dry-matter (DM) equation at seed 999: 0.8570 against 0.8486, at a fidelity of 0.9774, in 15 terms over eleven of the twelve variables.

(1) $${\hat{y}}_{\mathrm{tahini}}\approx 0.282x_{1}-0.450x_{2}-0.111x_{3}+0.173x_{7}-0.033x_{8}\;-0.176\sin(1.324x_{4}+3.043)\;+0.650\sin(0.460x_{1}-2.298x_{2}+1.187x_{4}-0.701x_{5}-0.155x_{8}+0.462)\;+0.734\sin(2.285x_{1}+0.678x_{2}-1.410x_{3}-0.054x_{4}+0.617x_{5}+0.276x_{8}+0.867)\;-1.014$$

(2) $${\hat{y}}_{\mathrm{DM}}\approx 1.414x_{1}+0.163x_{10}+0.272x_{11}-1.499x_{2}+0.739x_{3}-1.453x_{4}\;+0.389x_{5}+0.378x_{6}-0.271x_{7}-0.548x_{8}-0.413x_{9}\;-0.103\sin\left(2.539x_{3}-2.797\right)+1.866-1.002e^{-0.819x_{1}}$$

One extraction in ten failed, and the failure was not determined by the seed. Under each protocol one of the ten tahini seeds produced an unusable equation. Rerunning the same nominal extraction six times from the same network at seed 777, whose own external R^2^ reproduces to within 2 × 10^−7^, returned a usable equation in only four of the six runs; the six runs scored −0.755, −0.038, 0.950, 0.951, 0.953 and 0.958. The conversion is not reproducible between runs: the range of the equation R^2^ across repeated runs is 1.7 × 10^−3^ at the published seed, 0.14 at a second seed and 1.71 at this one, so naming the seed is necessary but not sufficient. Fidelity detects catastrophic failures but not smaller deviations: pooled over this study’s 288 extractions, a fidelity filter at 0.5 removes all four runs scoring below R^2^ 0.5 at the cost of one sound equation, yet an equation can still fall 0.10 R^2^ below its own network at a fidelity of 0.975 (Section S9). Four failures are too few to establish a general detection rate. The same variation was found at one and at four compute threads, so it is not explained by thread count (Table S9). By contrast, the exact route introduced below returned the same equation in all twelve repeated runs (range 0.000000; fidelity 0.999995–0.999996; Table S9).

**Figure 5:**
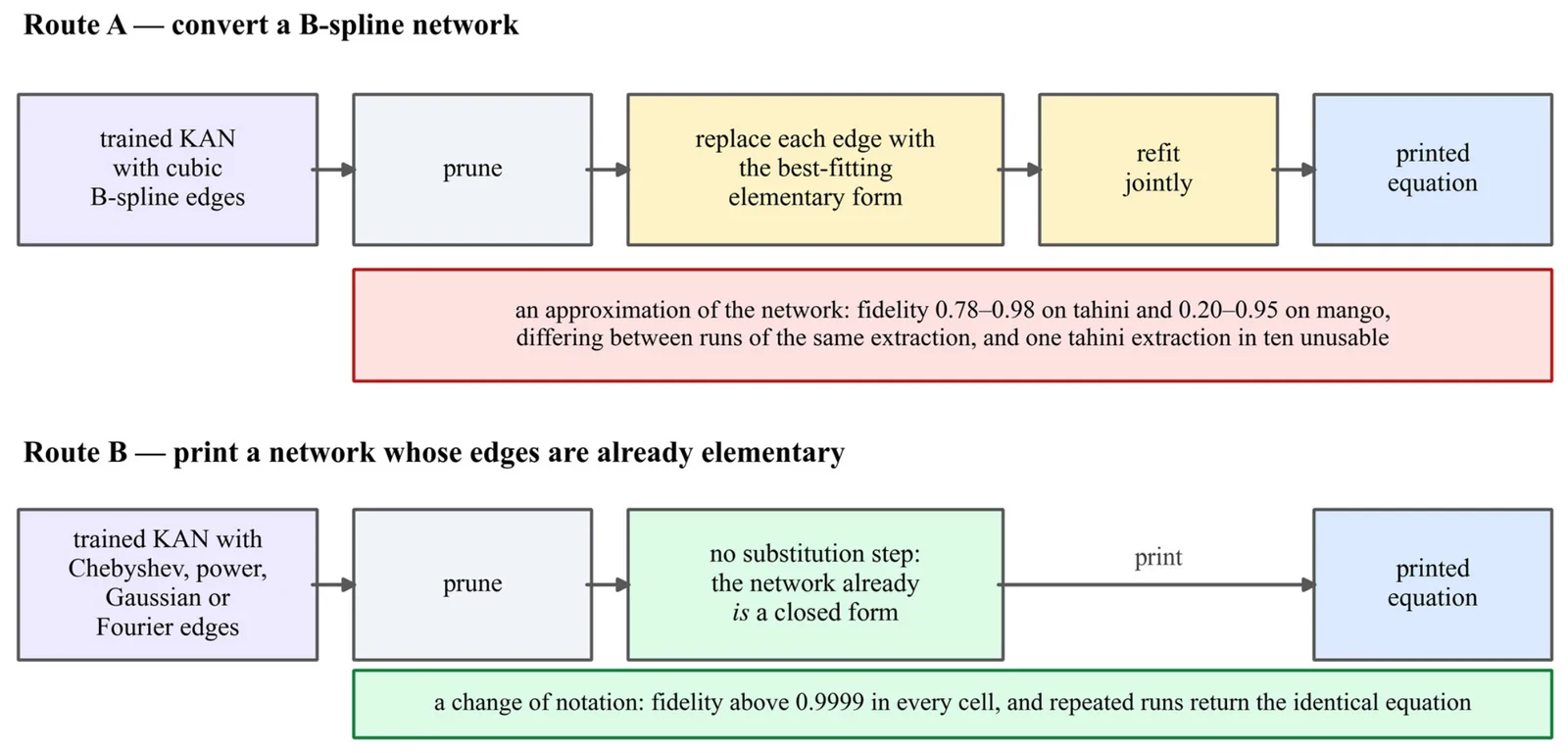
Two routes from a trained network to a printed equation. Route A (converted route) converts a B-spline network: each surviving edge is replaced by the best-fitting elementary form and the substituted network is refitted, so the printed formula approximates the network, by an amount that differs between repeated runs of the same extraction. Route B (exact route) trains a network whose edge functions are already elementary, so printing is a change of notation and the formula and the network are the same object; the repeated runs that return the identical equation are repeats from the same initialisation (Section 2.8, Table S9). All values shown are reported in Table 7 and in the text of this section.

Correcting the conversion does not eliminate this failure mode, whereas avoiding the conversion does (Figure 5). A residual fidelity of 0.985 still means the printed formula is an approximation. If edge functions are expanded in an already-elementary basis—Chebyshev polynomials [28], a truncated Fourier series, Gaussian radial functions [29] or plain powers—the trained network is itself a closed-form expression, and writing it out is a change of notation rather than a fit. The equation is then exact. Its fidelity departs from exactly 1 only because the printed coefficients are rounded to three significant figures, terms below 10^−3^ of the largest coefficient are omitted and the network computes in single precision; printed at full double precision, the equation of a refit of the same configuration reproduces its own network to within 10^−13^ in R^2^ (Section S25). With the basis chosen by inner cross-validation, no accuracy difference from the spline control or from the corrected conversion was detected (Table S14); this does not show that the accuracies are equal. On tahini’s latent scores the exact route reaches R^2^ = 0.986 ± 0.007 at a fidelity of 0.999996, against 0.987 ± 0.003 at 0.983 for the corrected conversion on identical inputs: the accuracy differs by 0.001 (adjusted *p* = 1.000), while the fidelity differs by four orders of magnitude in the residual, which follows from the construction of the exact route; with five seeds, even that difference is not significant (adjusted *p* = 0.094; Table S14). On mango’s latent scores it reaches 0.862 ± 0.006 at 0.999997 against 0.844 ± 0.007 at 0.953; neither the accuracy difference (adjusted *p* = 0.163) nor the fidelity difference (adjusted *p* = 0.071) is significant. The fitted model is also smaller (168 parameters against 234 for the identically trained spline control and 684 for the converted network on tahini; Table 7), although, following Section 3.6, no weight is attached to this difference.

The conversion’s fidelity is lowest and least stable for the equation of most practical use, namely the one expressed in named absorbance bands. On tahini its fidelity falls from 0.983 on latent scores to 0.779 on named bands (worst case 0.474). On mango, fidelity is very low: the converted equation averages R^2^ 0.107 ± 1.186 at a mean fidelity of 0.195, against a network scoring 0.772 ± 0.021 and an exact power basis reaching 0.815 ± 0.022; one of its five seeds returns an equation worse than the mean of the material it predicts (R^2^ −1.995, fidelity −2.478) and a second explains only about a third of that variance. Because of this spread, the 0.665 R^2^ gap between the Chebyshev basis selected by inner cross-validation (0.772 ± 0.042) and the conversion is not significant (adjusted *p* = 1.000; Table S14). Every elementary basis stays above 0.9999 fidelity, returning an equation faithful to its network at every seed on both matrices. Named absorbances are the recommended representation, and it is precisely in this representation that the standard conversion is least reliable.

None of the four fidelity gaps between the routes is significant (adjusted *p* = 0.094, 0.570, 0.071 and 1.000), and a comparison of means is not informative here, because the conversion’s fidelity is *unstable*, with an SD across seeds of 0.006, 0.172, 0.021 and 1.50 against at most 2.5 × 10^−5^ for any elementary basis (Table S14). This contrast in dispersion is reported descriptively and was not tested.

Advantages and limitations of the exact route. The printed equation is the model rather than an approximation of it, so the accuracy reported for the network is the accuracy of the formula, repeated runs return the same equation, and no seed here produced an unusable equation in either representation on either matrix; for these reasons, we recommend the exact route for reporting a calibration. The basis must still be chosen on held-out data like any other hyperparameter: a Fourier expansion is exact in the same sense but considerably less accurate, and it accounts for both of the differences that remain significant after adjustment (tahini named bands, by 0.075, adjusted *p* = 0.022; mango latent scores, by 0.071, adjusted *p* = 0.006; Table S14). The exact route offers no accuracy advantage: no gain over the corrected conversion was demonstrated in any of the four settings (adjusted *p* = 0.163 to 1.000; Table S14), and this null result is not evidence that the two routes are equally accurate. Its advantage is that the reported accuracy is the accuracy of the printed formula, which the conversion route cannot guarantee and, on named bands, does not achieve (mean fidelity 0.779 on tahini and 0.195 on mango; differences not significant; Table S14).

### 3.4. An Equation in Named Absorbance Bands

**Figure 6:**
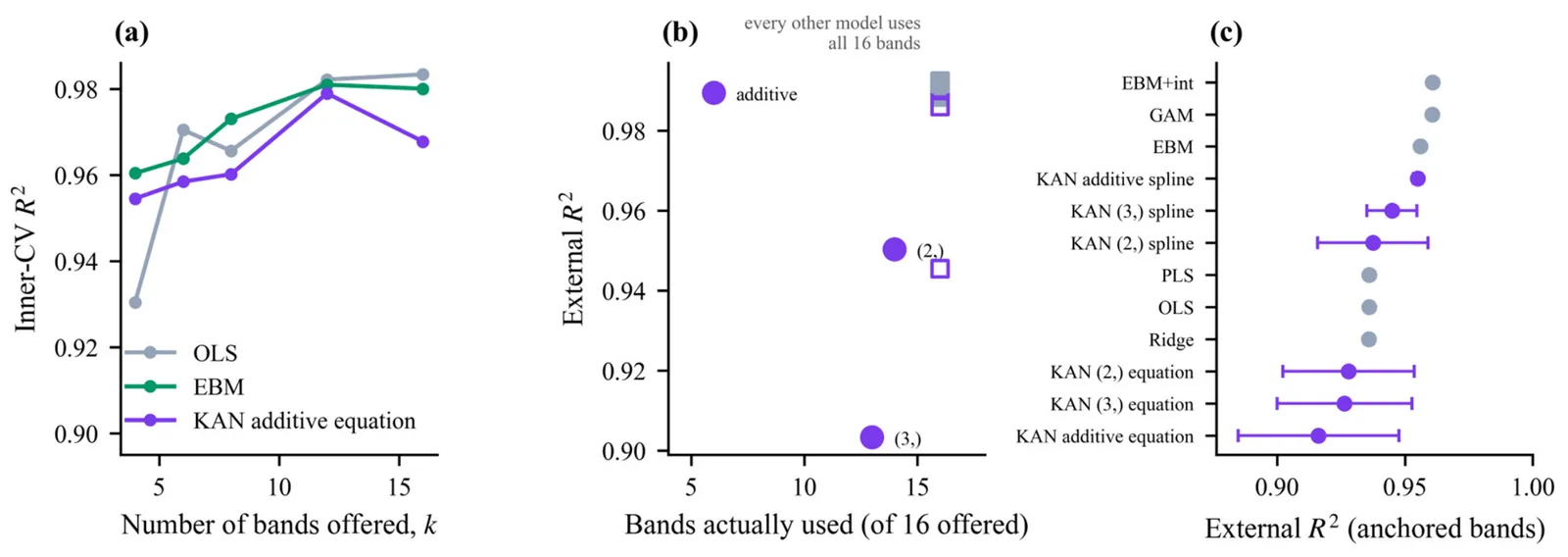
Calibration from named absorbance bands. (**a**) Inner-CV performance against the number of VIP-ranked bands offered, k, for the linear control (OLS), an EBM and the additive KAN equation. (**b**) External performance against the number of bands each model actually uses, with sixteen offered; only the printed KAN equations (filled circles) are labelled, because every other model uses all sixteen (open squares, the KAN spline networks; grey squares, the other models); R^2^ values are means and band counts medians over five seeds. (**c**) External performance when the eight bands are fixed in advance from the literature instead of selected from the data; here the non-linear networks and the additive glass-boxes have higher point estimates than the linear models, whereas all three printed KAN equations fall below OLS (differences not tested); points are means and error bars standard deviations over five seeds.

An equation in PLS latent variables cannot be evaluated directly: each variable is a dense combination of 1762 channels and therefore still requires the loading matrix. We therefore addressed this limitation by refitting the linear controls, the additive glass-boxes and the KAN on a small number of individually selected channels so that the variables are absorbances at named wavenumbers (Table 8, Figure 6). The tahini variables are SNV-corrected absorbances: evaluating the equation still requires the recorded spectrum (for the SNV step) but no longer a loading matrix. Channels were ranked inside each training fold by PLS VIP, and their number was chosen per model family by grouped inner cross-validation: sixteen for the linear controls and the spline additive model, twelve for the KAN and the explainable boosting machines (EBMs). With a linear control, accuracy saturates at 8–16 channels (external R^2^ 0.9923 with sixteen channels against 0.9943 for the full spectrum); with eight or more channels, averaging each channel over ±7.5 cm^−1^ raises R^2^ by at most 0.005 (Section S15).

Of the sixteen channels, two fall inside bands tabulated by the cited sources: 3005 cm^−1^, the =C–H stretch of *cis* unsaturation, and 1472 cm^−1^, the CH_2_ scissor. Nine lie inside a region that those sources describe but do not tabulate as a discrete band, and five (3022, 1502, 1487, 1447 and 1140 cm^−1^) fall outside every tabulated range; these are reported as unassigned rather than attributed to the nearest named vibration. Four of those five are among the eight channels on which Equation (3) is fitted, whereas neither of the two tabulated bands is. The printed calibration is therefore expressed entirely in named wavenumbers, but only partly in named vibrations (Section S10).

We tested the unassigned channels against instrumental explanations (Section S14, Table S16). Each varies monotonically with composition (|ρ| = 0.81–0.99 in the more responsive series, permutation *p* ≤ 0.0005), with the same sign in all five production lots in that series, and the association keeps the same sign and a similar size after adjustment for water vapour and lot. Under shuffled composition labels, 1502 cm^−1^ was never selected, but 1140 cm^−1^ was selected in 37% of 200 runs, and the regionally described channel 1286 cm^−1^ in 25%; for these two channels, therefore, the evidence against selection by chance is weak. The two leading attribution windows (Section S11), near 1490 and 1235 cm^−1^, are among the most composition-dependent channels (at 1235 cm^−1^, ρ = −0.98 for both adulterants). These channels are therefore composition-dependent but not vibrationally assigned. The amide III region of proteins (about 1230–1300 cm^−1^) [50] is a plausible contributor to the channels between 1235 and 1286 cm^−1^, but we do not claim an assignment.

The equation comes within 0.003 R^2^ of a linear model on the same bands (not tested) while using fewer than half of them. With sixteen bands offered, OLS reached R^2^ = 0.9923 and a spline generalised additive model (GAM) 0.9924, against 0.9895 ± 0.0009 for the additive KAN’s closed-form equation. The more relevant difference lies in the number of bands: the linear model requires all sixteen bands and seventeen coefficients, whereas the pruned equation retained a median of six of the sixteen bands in seven terms over five seeds, at a mean fidelity of 0.994 (with the twelve bands selected by inner cross-validation for the KAN, 0.9888 ± 0.0011 in ten terms over eight bands; Table 8). This parsimony claim differs from the one withdrawn in Section 3.6: it is not that the architecture uses fewer parameters (at matched capacity it does not), but that the *printed calibration* requires fewer measured channels. This parsimony is specific to the converted route; the exact route of Equation (3) retains every band it is offered.

Equation (3) is a calibration of that kind, printed from the exact route of Section 3.3 so that no conversion stands between the model and the formula. It is not the same fit as the converted equation described in the preceding paragraph: the exact route was offered eight of those sixteen bands (marked in Table S11) and retained all eight, so the relevant comparison is fidelity rather than band count. At training seed 42 (its initial weights were not seeded; Section 2.8) it reaches external R^2^ = 0.9841 over those eight bands in 31 terms, at a fidelity of one to six decimal places. Its variables are the SNV-corrected absorbances at the named wavenumbers, min–max scaled to [−1, 1] using their training ranges and clipped to that interval, and its output is the standardised tahini fraction: tahini (%) ≈ 76.16 + 32.73 ŷ, without the sum-to-100% rescaling that the network applies to its three outputs and that is included in the R^2^ above (Section S25). These constants are given in Table S29, so that the equation can be evaluated from a recorded spectrum without the training data. We show the *additive* variant rather than the one-hidden-layer arm selected by inner cross-validation because, with one univariate function per band, it can be read term by term as a per-band response.

(3) $${\hat{y}}_{\mathrm{tahini}}\approx 0.326A_{1140}^{4}-0.0423A_{1140}^{3}-0.305A_{1140}^{2}+0.573A_{1140}\;-0.305A_{1180}^{3}+0.437A_{1180}^{2}+0.471A_{1180}-0.293A_{1242}^{4}\;+0.0871A_{1242}^{3}+0.266A_{1242}^{2}+0.781A_{1242}-0.0927A_{1286}^{4}\;-0.113A_{1286}^{3}-0.0585A_{1286}^{2}-0.572A_{1286}+0.239A_{1447}^{4}\;-0.0424A_{1447}^{3}-0.274A_{1447}^{2}+0.349A_{1447}-0.126A_{1487}^{4}\;-0.0381A_{1487}^{2}+0.591A_{1487}+0.169A_{1502}^{4}+0.00801A_{1502}^{3}\;+0.131A_{1502}^{2}+0.0693A_{1502}-0.287A_{989}^{4}+0.558A_{989}^{3}\;-0.177A_{989}^{2}-0.0203A_{989}-0.555$$

Adding depth lowered the mean R^2^ and increased its seed-to-seed variation; on data-selected bands, the additive form therefore has both the highest mean R^2^ and, by a wide margin, the smallest seed-to-seed SD, although these differences were not tested (Section S13). Readability comes at a cost in length: an elementary basis prints every basis function it uses, so a degree-four expansion over eight bands prints 31 terms, against seven terms over six bands for the conversion. Compared like with like, however, the difference disappears, so the additional length is attributable to the additive form printed for legibility rather than to exactness itself.

All of the above uses data-selected bands, which favours a linear model because the selection criterion is a linear-projection importance. Repeating the comparison on eight bands fixed *in advance* from the literature [48,53] reduced the accuracy of every model but inverted the ordering of point estimates: the additive glass-boxes led, at 0.9549 to 0.9608, and the linear models trailed by about 0.02. When the variables are dictated by chemistry rather than optimised for a linear fit, the point estimates of the non-linear models are approximately 0.02 R^2^ above those of the linear models. This difference, however, is retained by the *networks* and not by the printed equations: on those bands the conversion costs the additive KAN 0.039 R^2^, leaving the equation 0.020 *below* OLS rather than 0.019 above it. This is the largest loss the additive form incurs anywhere in this study (Section S13), and it is the only result in this section that is unfavourable to the proposed approach. None of the differences in this paragraph was tested.

### 3.5. The Printed Equation Under a Distribution Shift

**Figure 7:**
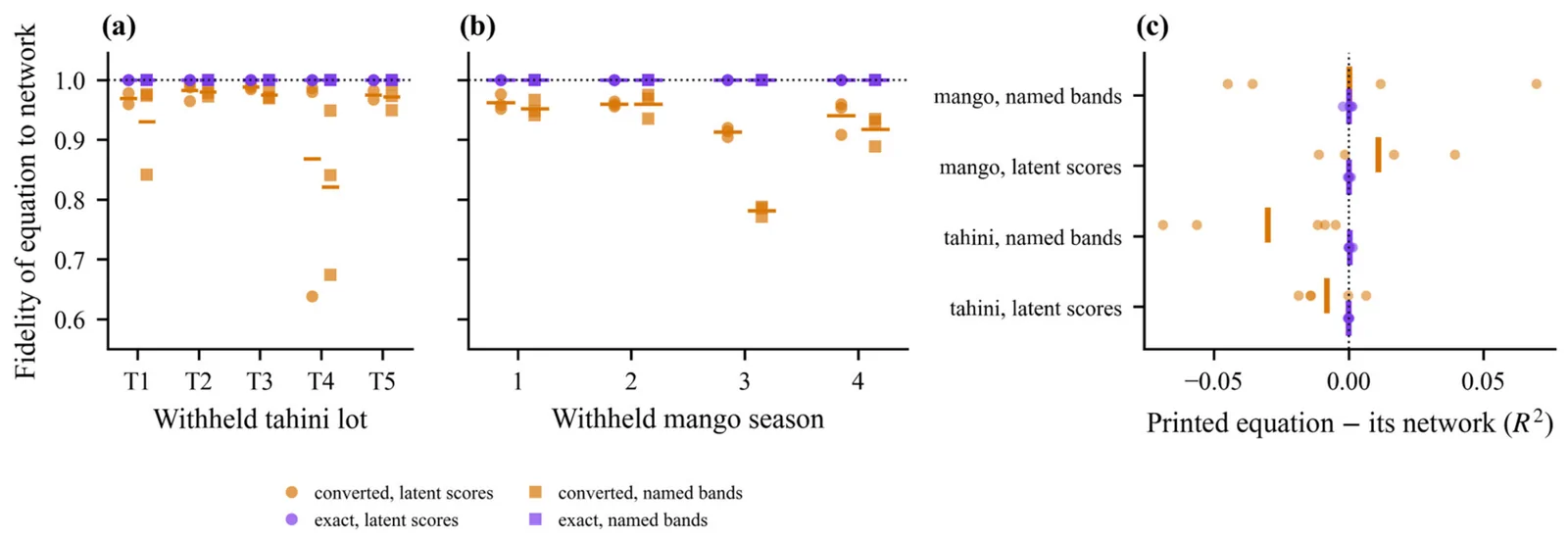
The printed equation under a distribution shift. (**a**,**b**) Fidelity of the printed equation to the network it was extracted from, on each withheld tahini production lot and each withheld mango harvest season; one point per seed, horizontal bars are fold means, and the dotted line marks exact agreement. (**c**) Accuracy of the printed equation minus that of its own network, for both routes and both representations; points are folds (averaged over seeds) and vertical bars the means over folds. The converted route is displaced from zero in both directions and by different amounts on the two matrices; the exact route sits on zero everywhere. No difference is significant after Holm adjustment (Table S14).

Everything in Section 3.3 is measured on held-out material from the same pool as the calibration. This leaves open the question most relevant to a printed calibration, since a published formula is more likely than a model in a repository to be applied to data outside the calibration distribution. The two routes lead to different expectations in this setting: for an elementary basis the equation and the network are the same function, so their agreement is independent of where they are evaluated. We tested this on the folds of Section S4, recording the accuracy of the network, the accuracy of the printed equation and the fidelity between them on the withheld material (Table 9, Figure 7). These folds use the model configuration carried over from the primary partition (the control design of Section 2.3), so their absolute accuracies are optimistic. The quantities compared here, an equation against its own network and the fidelity between them, are computed within one fitted configuration, so this selection leak affects both routes equally. Accuracies from the nested design are reported in Section S4.

The converted equation departs from its network on both matrices and with both representations. The fold-mean fidelity of the converted equation is 0.956 and 0.936 on tahini and 0.944 and 0.903 on mango, with worst folds of 0.868, 0.821, 0.913 and 0.781 and single runs as low as 0.638 (Figure 7a,b). On named bands, this low fidelity is not caused by the shift: the same route’s fidelity on the external test sets was already 0.779 on tahini and 0.195 on mango (Table 7). The exact route never falls below 0.9997 in any single run. Descriptively, this held in all four settings: when applied to material outside the training data, the converted equation no longer reproduced its network. The fold-level difference between the routes does not survive Holm adjustment in any setting (adjusted *p* = 0.052 to 0.263, Table S14).

The consequence for accuracy was *not* consistent across settings. On tahini named bands the two networks agreed to three decimal places (0.962 against 0.962; adjusted *p* = 1.00), but their equations did not: the converted one reaches 0.931, 0.030 below the model it was extracted from (adjusted *p* = 1.00), whereas the exact equation reproduces its network to a mean absolute difference of 0.0004 and at most 0.0015 in any fold. On mango named bands the same infidelity changes mean R^2^ by at most 0.0002 (adjusted *p* = 1.00). The four setting means ranged from −0.030 to +0.011, and none differed significantly from zero (adjusted *p* = 1.00, Table S14). A converted equation applied off-distribution may therefore lose or gain up to 0.07 R^2^ in a single fold, unpredictably (Figure 7c); this is a single-fold extreme rather than a typical value, and nothing identifies such cases in advance. Because such an error cannot be predicted, it cannot be corrected for, and this unpredictability, rather than a constant loss, is the argument for the exact route. Across all four combinations the exact route tracks its network to a mean absolute difference of 0.0005 and at most 0.0023 in its worst fold (Figure 7c).

None of these differences is significant under the corrected test: with four or five withheld folds, no comparison in this family survives Holm adjustment (Table S14). This reflects limited statistical power rather than evidence that the routes behave alike, and the argument is therefore based on unpredictability rather than on a mean penalty. Descriptively, the dispersion differs: the converted route’s fidelity varies across folds with SDs of 0.050, 0.067, 0.023 and 0.083, against 1 × 10^−6^ to 3.4 × 10^−5^ for the exact route; this contrast was not itself tested.

On the named bands of mango, the matrix expected to be non-linear, the exact non-linear equation also had the highest accuracy by point estimate. Averaged over the four withheld mango seasons, it reaches 0.763 against 0.757 for OLS on the same bands and 0.732 for the B-spline network. Section 3.4 anticipated that a non-linear equation would be most useful on a non-linear matrix, a prediction that tahini could not test. The margin is 0.006 with a fold-to-fold SD of 0.052 (adjusted *p* = 1.00), so it is an ordering of point estimates and not a demonstration; it is reported because it is in the direction the argument predicts, and because reporting only significant comparisons would omit the one setting in which the prediction could be tested.

Two qualifications apply to this interpretation. First, a low fidelity does not automatically mean a worse prediction: the conversion’s worst run has a fidelity of 0.638 while its equation trails its network by only 0.008 on average, because fidelity measures whether the equation *is* the model, not whether it is accurate. Second, on the latent scores of both matrices the elementary network itself transferred less well, with point estimates below those of OLS (0.813 against 0.817 on mango, 0.912 against 0.939 on tahini; adjusted *p* = 1.00), so the leading result above is specific to named bands and not to either matrix as a whole. Every route degraded under transfer except the mango named-band conversion, which had already performed poorly on its external test (0.107; Table 7); none of these results should be read as evidence that any route generalises well.

### 3.6. Accuracy, Stability and Contribution of the Equation

Three claims commonly made in interpretability studies are not supported by this study.

The KAN does not provide parameter efficiency. The KAN uses fewer trainable parameters than the deep baselines (Figure 3f), but matching a larger, differently tuned model shows only that the task did not need the larger model’s capacity; the test that separates the two explanations is a baseline built to the *same* parameter count. This test was run in twelve configurations (three capacities, two training rules, and the KAN at both its published configuration and the one selected by its own inner search), and the parameter-matched MLP had the higher mean R^2^ in every one of them, by margins from 0.0005 to 0.061 R^2^ (Table S6). Because both architectures were trained with the same five seeds and scored on the same external test set, the differences were tested in pairs by seed (Section S24, Table S28). By a simple paired *t*-test, none of the four tahini differences was significant (Holm-adjusted *p* ≥ 0.41), whereas on mango the MLP was significantly more accurate in five of the eight comparisons (adjusted *p* ≤ 0.029); with the Nadeau–Bengio correction used for the other seed-repeated comparisons (Section 2.5), no difference was significant on either dataset (smallest adjusted *p* = 0.066), and on the corrected variance equivalence within ±0.02 R^2^ could not be shown for any pair. As every seed shares one test set, these tests measure training randomness rather than generalisation to new material (Section 2.5). The two architectures are therefore comparable in accuracy, the MLP being ahead on every point estimate, and no KAN advantage was found. We accordingly withdraw parameter efficiency as an argument, although our companion study made this claim [42], and we claim no accuracy advantage in any setting. This is consistent with the controlled architecture literature, which finds MLPs at least as good as KANs at matched parameter counts on all task families except symbolic formula representation [54]. The comparison is not an artefact of the training budget (Section S21): trained for the MLP’s number of updates, the KAN scored lower than at its own budget on both datasets (tahini 0.962 against 0.980; mango 0.854 against 0.860), and the MLP trained for the KAN’s number of updates still scored higher than the KAN at its own budget (0.986 and 0.883).

Nor does the KAN provide stability. Across five seeds on tahini the KAN’s SD (not tested) is 0.0061 under the published recipe against the MLP’s 0.0027, and 0.0408 against 0.0032 under an early-stopping training procedure (Table S6, block 3) that improves the MLP while costing the KAN 0.021. Its own inner cross-validation also selected a configuration that performed worse externally than the published one, so the architecture is not reliably self-tuning at this size. The same sensitivity appears wherever the model extrapolates beyond its calibration pool. It is the more variable model when a whole lot or season is withheld with the configuration carried over from the primary partition, although with selection nested inside each fold it is the least variable over tahini lots (SD 0.017, against 0.065 for PLS; Section S4); over withheld mango seasons its SD is 0.051, against 0.062 for PLS and 0.025 for the CNN. Its detection limit also increased the most when every authentic lot, rather than one, was used as the blank (Section 3.2), and over 30 repeated grouped hold-outs with nested selection it had the largest spread of the four leading models (SD 0.048; Section S23). In the frozen design, part of the KAN’s weaker transfer across production lots appears to be a property of the published configuration rather than of the architecture; with selection nested inside each fold, the published configuration had the highest mean over withheld lots (Section 3.2; not significant). With the favourable single factors of Section S5 combined (SNV with first-derivative SG preprocessing, grid 3, spline order 4, width 5), the KAN reaches R^2^ = 0.992 ± 0.004 on the external test set over five seeds, against 0.980 ± 0.012 for the published configuration in the same run. These seeds and this training code differ from those of Table S6, which is why the published configuration scores 0.980 ± 0.012 here and 0.986 ± 0.006 there, a difference of the order of the seed-to-seed variation (Table S25). Averaged over withheld production lots, with each configuration held fixed rather than re-selected inside each fold, the combined configuration reaches R^2^ = 0.975, with a worst lot of 0.937, against 0.940 (worst 0.841) for the published configuration and 0.975 (worst 0.952) for PLS; none of these differences was tested (Section S22). Because the combination was chosen from the ablation of Section S5, by cross-validation on the training partition, and then held fixed, these withheld-lot values are optimistic relative to a nested design, in which the published KAN reached 0.968 (worst 0.948) and PLS 0.950 (worst 0.837; Section 3.2). The published configuration was therefore probably not the best one available for lot transfer, and the KAN’s sensitivity to the random seed at this sample size remains a cost. The flexible models are also data-limited: retrained on subsets of the 38 training samples, the KAN reached R^2^ = 0.813 ± 0.232 with 10 samples, against 0.990 for PLS, rising to 0.982 ± 0.010 with all 38 (Section S20).

**Figure 8:**
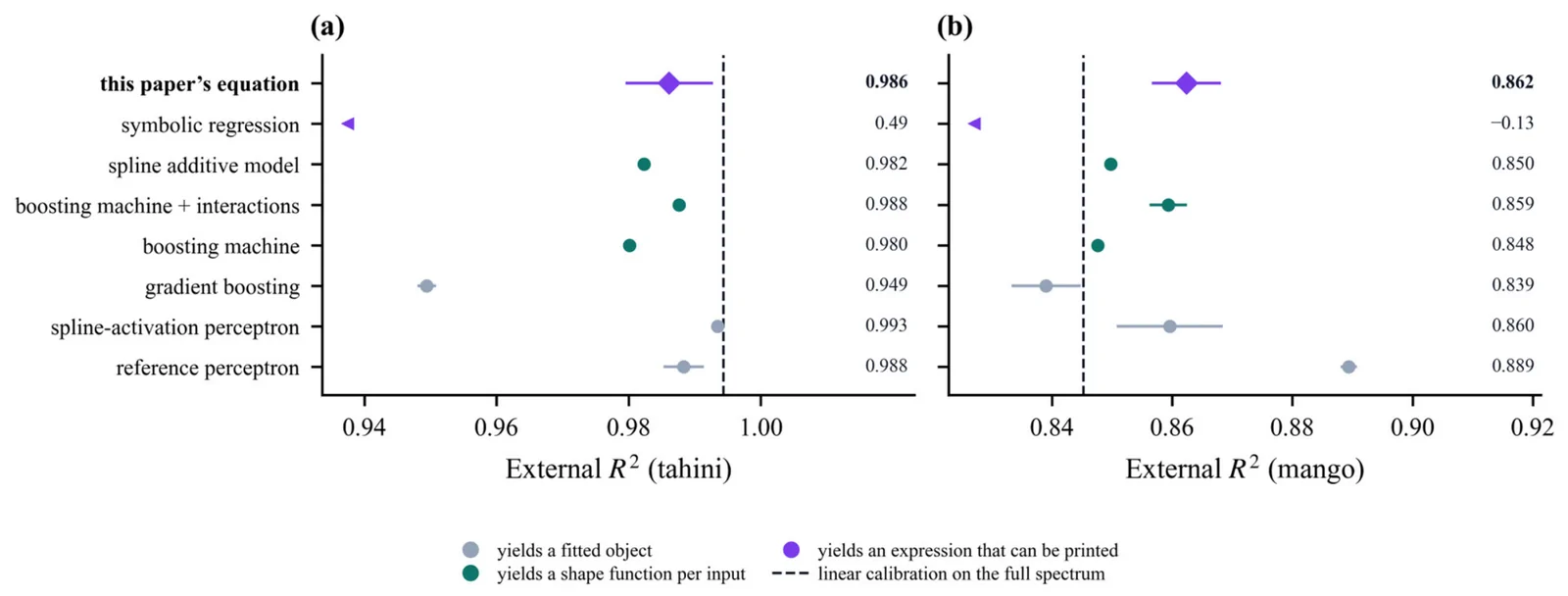
External accuracy against the form of the fitted model’s output. (**a**) Tahini; (**b**) mango. Models are grouped by whether they yield only a fitted object, a shape function per input that can be plotted, or an expression that can be printed; the order of the groups is not a ranking of quality. Error bars are standard deviations over three seeds (five for “this paper’s equation”, the exact-route equation of Section 3.3 on the same latent scores, with its basis selected by inner cross-validation: Chebyshev on tahini, Gaussian on mango); a model whose score falls outside the plotted range is drawn at the left edge with its value given. The dashed line is the linear calibration on the full spectrum, which is an equation only in the sense that it comes with an unpublished loading matrix.

Nor does the KAN provide an accuracy advantage over existing glass-box models, since a depth-one network is a GAM. On tahini the EBM [55] reaches R^2^ = 0.980 ± 0.000, the spline GAM [56] 0.982 ± 0.000 and the EBM with pairwise interactions 0.988 ± 0.001, against 0.986 ± 0.007 for the Chebyshev equation of Section 3.3 on identical inputs; on mango the same three reach 0.848, 0.850 and 0.859, respectively (Table 10, Figure 8). The paired tests were run over the three seeds these families share, on which the equation averages 0.9892 rather than the 0.9861 of its own five seeds, so their margins are not the difference between the two means given above. The equation’s margins over the plain EBM (+0.009) and over gradient boosting (+0.040) survive adjustment (*p* = 0.0497 and 0.004), whereas its differences from the other five do not. Little weight is placed on the two significant margins: the test compares three training seeds on one fixed test set, so it shows that the margins exceed run-to-run variation, not that they would hold on new material (Section 2.5), and the EBM and GAM scores vary by less than 0.002 across seeds (Section S12). The KAN therefore offers no meaningful accuracy advantage over models that have been available for a decade, and the case for it rests on the form of its output.

The models differ in the form of their output. The additive glass-boxes each return a *shape function* per input, as a plot or a lookup table. Such output is not a compact equation: a spline shape function or a lookup table can be tabulated, but not written as a short expression of elementary functions that can be evaluated by hand or substituted into a downstream expression.

The model with the highest mean R^2^ on tahini, an MLP with spline activations, cannot be printed as an equation, for a structural reason. With learnable B-splines on the nodes rather than the edges, an MLP reaches 0.993 ± 0.001 on tahini—the highest mean on these inputs, the KAN included—and 0.860 ± 0.009 on mango, reproducing on food spectra the controlled architecture literature’s finding that the advantage attributed to the KAN structure belongs substantially to the spline basis [54]. Because the activation is placed on the node, however, it is applied after the layer has mixed its inputs, so there is no closed form in the original variables to extract: the model is more accurate but cannot be written as an equation. Symbolic regression, the most direct alternative, reaches R^2^ = 0.490 ± 0.333 on tahini and −0.134 ± 0.464 on mango, worse than its own training mean; it is about 0.5 R^2^ below the equation, a difference that is *not* significant (adjusted *p* = 0.64) because its seed-to-seed SD is 0.33–0.46 R^2^.

Figure 8 summarises the comparison. Of the models that reach the accuracy of this calibration, the MLPs and the boosting machines yield no equation; of the models that yield an equation, symbolic regression is unusable and the rest are linear. It cannot be concluded that the KAN equation is the most accurate writable model on these data: on tahini, OLS on the same sixteen named bands reaches 0.9923 against the equation’s 0.9895, and the full-spectrum linear calibration reaches 0.9943 (not tested). For a matrix that follows Beer–Lambert mixing, a linear equation is the appropriate model. The remaining claim is narrower but still useful: among the non-linear models that reach this accuracy, the KAN equation is the only one that can be written down as a compact closed-form expression.

### 3.7. Comparison with Previously Published Models

Mango dry matter. This benchmark is unusually well documented in the literature because the same external partition has been used repeatedly: the originating study reported a global PLS calibration at RMSEP = 1.014% dry matter [7], a follow-up reached 0.881% with a locally optimised variance regression and 0.839% with an ensemble [57], chemometric preprocessing with a CNN brought it to 0.79% [21], and the current best result, 0.77%, was set by a one-dimensional CNN [43]. Our PLS baseline reaches 1.050%, within 4% of the published global PLS figure, which establishes that our pipeline reproduces the published linear benchmark. Relative to this reference, the KAN’s 0.988% is nominally 0.026 percentage points lower, but this margin is smaller than the 0.036 percentage points by which our PLS differs from the published PLS, so the result indicates agreement with the published linear benchmark rather than an improvement on it. The KAN does not approach the published non-linear results, and no such claim is made: those results were obtained with ensembling, local modelling, data augmentation and much larger networks, none of which yields a calibration a reader can write down.

Tahini adulteration. No published calibration exists for this dataset, which is released with this paper. ATR–FTIR with chemometrics has been used on cold-pressed sesame *oil* [3] and NIR on sunflower kernel paste [9], but neither addresses sesame *paste* adulterated across the complete mixing range with two adulterants. The internal comparator is the companion MIR study, whose three matrices gave cross-validated R^2^ of 0.864, 0.968 and 0.774 [42]. Tahini’s R^2^ is higher than any of them, but this comparison is of limited value: those calibrations span 0–30% or 0–50% adulterant against tahini’s full 0–100%, and R^2^ rises with the width of the calibration range. The tahini dataset has the widest range and the cleanest two-component chemistry; it is not demonstrably an easier problem.

Detection limits and the role of the method. Table 11 compares the detection capability of the present screen with published methods for related matrices. The out-of-fold detection limits obtained here (8.9% for PLS, 14.3% for the KAN and 21.6% for SVR) bracket the 10.78% authenticity threshold reported for ATR–FTIR with a one-class model on tahini spiked with peanut or sunflower oil [10]; only the PLS and MLP limits (8.9 and 10.3%) are lower. That threshold comes from one-class authentication of oil-adulterated tahini, whereas ours are regression-based limits for whole pastes, so the two are of the same order but not directly comparable. With the selection nested inside each withheld lot, the limits were 10.3% for the KAN, 17.5% for the MLP and 19.5% for PLS (Section 3.2). These limits are higher than the approximately 4% reached by proton nuclear magnetic resonance (^1^H NMR) spectroscopy with a marker compound in nut powders [58] and the 5% reached by gas chromatography with chemometrics for vegetable oils in sesame oil [11]. Targeted confirmatory methods work at trace level: real-time polymerase chain reaction (PCR) detects sesame at 0.005% [12], real-time PCR and enzyme-linked immunosorbent assay (ELISA) quantify hazelnut in nut paste at 2 and 1 mg/kg [13], and liquid chromatography–tandem mass spectrometry (LC–MS/MS) quantifies peanut at 10 mg/kg [14]. These trace limits are not directly comparable with the bulk limits of the screen. The method is intended as the first tier of a two-tier strategy, as in published screen-then-confirm workflows [59,60]. A positive screen requires confirmation by a targeted method. A negative screen cannot exclude trace peanut, an allergen whose presence must be declared [61] and which targeted methods detect at mg/kg levels. The screen addresses percent-level, economically motivated substitution, in which a cheaper paste replaces a substantial fraction of the sesame. The levels at which such substitution occurs in commercial tahini are not documented, and substitution below the detection limit would go undetected.

On the KAN literature. KANs have already been applied to food and agricultural spectroscopy by several groups, as the Introduction records, so we make no priority claim for the application area. In every such study that we could access, the network is used to obtain an accuracy figure and the trained calibration is not reported as an equation. Reference [37] is excluded from this statement because its full text could not be obtained and its abstract is neither distributed by the publisher nor deposited with Crossref, OpenAlex or Semantic Scholar. The distinction on which this study rests is therefore not that a KAN can be applied to food spectra, which has already been shown, but that the trained calibration can be reported as a closed-form equation, at a cost that is measured here.

### 3.8. Limitations

The architecture provided no accuracy advantage, and this is the principal limitation of the study. At a matched number of trainable parameters, an MLP was comparable in accuracy and had the higher mean R^2^ in all twelve configurations, significantly so in five mango configurations by a simple paired test but in none after variance correction (Section 3.6 and Section S24), and the additive glass-boxes reached accuracy similar to that of the printed equation on both matrices. The contribution of this work lies in the form of the calibration, not in its accuracy.

The tahini blends are laboratory mixtures, and five authentic lots constitute a narrow base. Composition labels are exact and mixing is uniform, whereas neither holds for commercial adulteration, so every tahini figure reported here is an upper bound on what the method would achieve on retail material. With the selection nested inside each fold, withholding a single lot lowers the KAN’s R^2^ from 0.987 to a five-fold mean of 0.968, with a worst fold of 0.948, and every model lost accuracy on a withheld lot or season (Section 3.2 and Section S4). On mango, the prediction errors of PLS and the KAN were associated with cultivar (only PLS after Bonferroni correction), and the two cultivars with the highest dry matter were underpredicted by every model. The calibration and test seasons also differed in maturity (89% against 39% of spectra from hard-green fruit), and fruit size and skin properties were not recorded, so their effect could not be examined (Section S18). On nested subsets of the 38 training samples, PLS reached R^2^ 0.990 with 10 samples, whereas the MLP, the CNN and the KAN reached 0.917, 0.888 and 0.813, respectively, and improved steeply up to 38 samples (Section S20). With fewer training samples than were available here, a linear calibration is therefore the appropriate choice. Five lots and four seasons are too few to establish transfer to new production. A calibration intended for deployment would require a far broader authentic base before the detection threshold is fixed. Neither dataset tests transfer to a second instrument, which is the usual obstacle to deployment.

The detection limit is a screening figure and is less favourable than the hold-out estimate suggests. The limit of 3.3–3.5% estimated for the three leading models from a test partition containing one authentic lot rises to 8.9% adulterant for PLS and 14.3% for the KAN when all five lots serve as blanks in turn, and to 10.3–19.5% for the leading models in the nested leave-one-lot-out validation (Section 3.2). These out-of-fold figures, not the hold-out value, are the ones that should be quoted. These levels do not constitute trace detection, and they apply only to samples similar to the calibration population. At 4–12% the adulterant type was usually identified, but these levels lie below or near the detection limits and were not quantified. Moreover, authentic tahini from an unseen lot produced false positives, so a positive screen requires confirmation by a targeted method. Both detection-limit estimates are conditional on the nominal mixing ratios being accurate.

The equation is an empirical calibration and does not describe a mechanism. It reproduces the trained network, but it is fitted to these data and its coefficients are not transferable constants. Five of the sixteen named channels are not assigned to a tabulated vibration; they vary with composition consistently across lots and after adjustment for water vapour (Section S14), but their chemical origin is not established. The water-vapour level also correlated with the sunflower fraction, so measurement conditions were not fully independent of composition, although this does not explain the channel selection (Section S14). The reliability of the mapping from model variables to chemistry depends on the matrix. On tahini, the attribution accounts of the six models agreed on the wavenumber axis at a mean Spearman correlation of 0.61; on mango, the mean was 0.22, with three of the twenty-one pairs correlating *negatively* and the network’s intrinsic attribution correlating at 0.05 with a block-permutation attribution of the same fitted network (Section S11). Where many weightings of a collinear spectrum give similar predictions, attribution is not identifiable. The exact route guarantees that the printed equation *is* the model, but not that its variables identify the chemistry. The named-band equation also still requires the full recorded spectrum, because its inputs are SNV-corrected.

An equation obtained by converting a B-spline network is not a reproducible calibration, and several comparisons are underpowered. At the published seed, the run-to-run range was 1.7 × 10^−3^, and at another seed it was 1.71 (Section 3.3 and Section S9); any single quoted equation is therefore one draw from a distribution, which is reported alongside it. The extraction results were also obtained with the published KAN configuration, which was not the best available for lot transfer in the frozen design (Section S22). The corrected resampled *t*-test with Holm adjustment is deliberately conservative, and with three seeds or five folds it has very little power (Sections S12 and S23); a non-significant result is therefore not interpreted as equivalence, which was tested separately only where stated.

The standard conversion should not be used to produce an equation intended for use outside the laboratory in which it was calibrated. Although this is a recommendation rather than a limitation, it follows directly from Section 3.3 and Section 3.5: an equation extracted in this way and applied off-distribution is not the model that was validated, and the formula itself gives no indication of the discrepancy. Where the calibration is to be printed and used, an edge basis that is already elementary avoids the problem, provided that the basis is itself selected on held-out data; no accuracy difference was detected on the external test sets (an underpowered comparison). None of these results makes the method deployment-ready, as stated explicitly in the companion study [42]. The study provides a calibration whose functional form can be inspected. It was evaluated against strong baselines on two matrices with different physics, under validation in which model selection was confined to the training data and nested inside each withheld lot or season (except for the equation-transfer analysis of Section 3.5, the out-of-fold detection limits of Table 4 and the combined configuration of Section S22, which use carried-over configurations).

## 4. Conclusions

We evaluated a KAN, a calibration model that can be written as a closed-form equation, on two contrasting food-spectroscopy problems: tahini adulteration by ATR–FTIR, on a dataset released with this paper, and dry matter in intact mango by NIR absorbance, on a public benchmark with a published across-season external test. The KAN was compared against five established models under one grouped protocol, with model selection confined to the training data and, for withheld lots and seasons, nested inside each fold.

No accuracy advantage is claimed. In twelve configurations comparing the KAN with an MLP matched to its trainable-parameter budget, the MLP had the higher mean R^2^ in every one, by margins from 0.0005 to 0.061 R^2^, the smallest margin occurring in the pairing most favourable to the KAN. In tests paired by seed, the two architectures were comparable in accuracy, and no KAN advantage was found: no difference was significant on tahini, and on mango the MLP was significantly more accurate in five of the eight comparisons by a simple paired *t*-test, but in none after the variance correction used for the other seed-repeated comparisons. Parameter efficiency, an argument made in our companion study, is therefore withdrawn, and no superior accuracy is claimed in any setting. When whole production lots were withheld, the KAN was among the leading models, but with five lots no difference between the KAN, PLS, the MLP and the CNN was significant.

The contribution is that the calibration can be reported as a closed-form equation, and that doing so need not entail a detectable loss of accuracy on the external test sets, although on withheld tahini lots the exact latent-score network scored below the B-spline network (0.912 against 0.948, with the configuration carried over from the primary partition, Table 9; not significant). On tahini, most of the accuracy lost in converting a trained network to closed form was attributable to the standard conversion rather than to the architecture: in the standard conversion, each edge is fitted against a single random mini-batch and the converted model is never refitted. Correcting both deficiencies raised the median external R^2^ of the tahini equation from 0.952 to 0.988 and of the mango equation from 0.846 to 0.851. The conversion can also be dispensed with altogether. With edge functions expanded in a basis that is already elementary, printing the trained network is a change of notation rather than a fit, and the printed equation reproduces the network it came from to between four and six decimal places on the external test sets (at least three on withheld lots and seasons), and to six decimal places for Equation (3). In addition, the equation can be written in individually named absorbance bands rather than latent variables, so that it can be evaluated from a printed formula and a small number of channel readings, with no loading matrix, although its SNV-corrected inputs still require the recorded spectrum.

This distinction has practical consequences. On material from a lot or season that the model had not seen, the converted equation no longer reproduced the model from which it was extracted: with the configuration carried over from the primary partition, fidelity fell to 0.78 in the worst fold and to 0.64 in the worst single run; an exact equation cannot diverge in this way, because it *is* the same model written differently. The effect of this loss of fidelity on accuracy was unpredictable: across the four dataset–representation combinations it ranged from a loss of 0.030 R^2^ (tahini, named bands) to a gain of 0.011 (mango, latent scores), none of which was significant. The conversion was also not reproducible, even at a fixed seed: from one trained network, whose own accuracy reproduces to within 2 × 10^−7^, repeated runs produced equations scoring from −0.755 to 0.958. Neither failure is visible in the formula itself. We therefore recommend computing the agreement of the printed equation with its own network, treating a low value as decisive and a high one as weak reassurance, and preferring a route in which no approximation is introduced at all.

Three findings concern how such work should be reported. A detection limit estimated from the single authentic lot in our test partition is 3.3–3.5% adulterant for the three leading models; estimated when all five lots serve as blanks in turn, it is 8.9% for PLS and 14.3% for the KAN (10.3–19.5% for the three leading models with the selection nested inside each withheld lot). A detection limit is therefore a property of the blank population as much as of the calibration. Separately, a discrepancy of 0.02 R^2^ between two runs of one comparison was caused by an inconsistently applied scoring convention, not by floating-point non-determinism, which contributed at most 3.5 × 10^−4^ (Sections S7 and S8). Finally, results must be reported for the range of adulteration the validation actually covers: at 4–12%, the adulterant type was usually identified, but these levels lie below or near the detection limits and were not quantified, and authentic lots withheld from the calibration set produced false positives, at rates that differed widely between lots (Table 5).

The interpretable non-linear model is not advantageous in every setting. On tahini, a lipid-in-lipid dilution that follows Beer–Lambert mixing closely, OLS on the same named bands had a higher point estimate on a same-pool hold-out (0.9923 against 0.9895; not tested). Whether interpretable non-linearity improves accuracy on a non-linear matrix was not demonstrated here (on mango the KAN differed significantly only from RF). The architecture also provided no accuracy advantage over the additive glass-box models available for the past decade; its advantage is that its contents can be printed in full. None of the models generalised well beyond its calibration pool. That the model can be printed does not mean that its variables identify the chemistry: on mango, where attribution methods disagree, they do not.

The practical conclusion of this work is limited but useful: a calibration for a food-authenticity or quality problem can be obtained as an exact equation in eight named bands (31 terms; tahini external R^2^ 0.984, against 0.992 for OLS on sixteen bands; not tested), with no accuracy advantage over an MLP of the same size. This holds only if the equation is extracted correctly, checked against the model it came from, and reported with the distribution it was drawn from rather than as a single number. The resulting calibration is a screening tool for material resembling its calibration population, to be confirmed by targeted analysis, not a deployment-ready method.

## Figures and Tables

**Table 2:** Tahini: Performance on the group-independent test set (17 physical samples, 408 spectra), for the tahini fraction. Intervals are cluster bootstraps that resample whole physical samples. CI, confidence interval; CV, grouped inner cross-validation on the training partition; SD, standard deviation.

Model	CV R^2^ (Mean ± SD)	Test R^2^	95% CI	RMSEP (% Tahini)	MAE (% Tahini)	RPD	Detection F1	Trainable Parameters	What It Yields
PLS	0.992 ± 0.005	0.994	0.978–0.995	2.50	2.11	13.27	1.00	21,180	Linear coefficients
SVR	0.895 ± 0.054	0.925	0.816–0.973	9.13	7.00	3.64	0.79	—	No closed form
Random forest	0.887 ± 0.028	0.919	0.662–0.947	9.46	6.94	3.51	0.93	—	Impurity importance
MLP	0.980 ± 0.006	0.988	0.955–0.992	3.61	3.16	9.21	1.00	2947	No closed form
1-D CNN	0.946 ± 0.019	0.967	0.836–0.986	6.00	3.93	5.54	0.98	19,363	No closed form
KAN	0.942 ± 0.031	0.987	0.946–0.992	3.81	3.03	8.73	1.00	684	Closed-form equation

**Table 3:** Mango: performance on the published across-season external test set (season 4; 1448 spectra, 18 populations, none seen in training).

Model	CV R^2^ (Mean ± SD)	Test R^2^	95% CI	RMSEP (% Dry Matter)	MAE (% Dry Matter)	RPD	Trainable Parameters	What It Yields
PLS	0.854 ± 0.040	0.845	0.726–0.895	1.050	0.822	2.54	2496	Linear coefficients
SVR	0.822 ± 0.023	0.844	0.755–0.885	1.054	0.805	2.53	—	No closed form
Random forest	0.804 ± 0.035	0.793	0.664–0.847	1.213	0.923	2.20	—	Impurity importance
MLP	0.861 ± 0.018	0.861	0.778–0.898	0.993	0.753	2.69	10,881	No closed form
1-D CNN	0.835 ± 0.039	0.845	0.740–0.894	1.052	0.784	2.54	9041	No closed form
KAN	0.840 ± 0.030	0.863	0.777–0.901	0.988	0.753	2.70	8996	Closed-form equation

**Table 4:** The detection limit estimated two ways, by the calibration-slope convention LOD = 3.3σ/S. The hold-out columns take the blank from the single authentic lot in the test partition and are given for comparison only. The out-of-fold columns average each spectrum’s predictions over three independent grouped five-fold partitions, in which each of the five lots supplies the blank while it is held out; the bootstrap resamples whole lots for the blank dispersion and whole samples for the calibration slope. The ratio is the factor by which the estimate changes. The final column gives the limit obtained from each of the three partitions separately. Configurations are those selected on the primary partition; the nested leave-one-lot-out limits are given in Section 3.2 and Section S17. These limits apply only to samples similar to the calibration population (Section 3.2). Differences between models are tested in Table S15; only those involving SVR are significant. The MLP and CNN rows are from the original run; the released code reproduces the other rows exactly and these to within 0.1 percentage point.

Model	Out-of-Fold R^2^	LOD, Hold-Out (%)	LOD 95% CI, Hold-Out	LOD, Out-Of-Fold (%)	LOD 95% CI, Out-of-Fold	Ratio	LOQ, Out-of-Fold (%)	LOD per Repeat (%)
PLS	0.990	3.46	3.41–3.56	8.93	4.57–11.13	2.6×	27.06	10.31, 7.53, 9.59
SVR	0.918	19.61	18.93–21.29	21.59	16.27–25.37	1.1×	65.43	23.71, 22.45, 23.92
Random forest	0.941	22.40	21.33–23.33	13.11	9.18–15.63	0.6×	39.73	14.09, 16.79, 17.01
MLP	0.985	3.29	3.23–3.35	10.32	3.60–13.96	3.1×	31.28	10.43, 9.99, 11.79
1-D CNN	0.966	6.12	5.98–6.27	14.88	7.15–19.51	2.4×	45.09	13.48, 16.55, 16.26
KAN	0.973	3.44	3.35–3.55	14.27	5.04–18.22	4.1×	43.24	27.02, 11.93, 12.56

**Table 5:** False-positive rate on authentic tahini for each production lot, in the nested leave-one-lot-out validation (Section S16). Each lot (T1–T5) was withheld in turn together with every blend prepared from it; the model configuration and the detection threshold were chosen without that lot (threshold 92.5, 89.0, 91.5, 92.5 and 93.0% predicted tahini for T1–T5). Columns T1–T5 give the percentage of that lot’s authentic spectra (150 for T1, 151 for the others) predicted below the threshold; “All lots” pools the 754 authentic spectra. “Lots flagged” counts the lots whose replicate-mean prediction fell below the threshold, that is, sample-level false positives. “Mean adulterant” is the mean predicted adulterant fraction on authentic spectra. Each lot is a single authentic sample, so its replicate spectra are not independent observations and the lot-level counts are coarse; differences between models were not tested (Section S16). These rates apply only to authentic material similar to the five calibration lots.

Model	T1 (%)	T2 (%)	T3 (%)	T4 (%)	T5 (%)	All Lots (%)	Lots Flagged	Mean Adulterant (%)
PLS	100.0	0.0	0.0	0.0	97.4	39.4	2 (T1, T5)	6.9
SVR	100.0	55.6	30.5	86.8	88.7	72.3	4 (T1, T2, T4, T5)	15.6
Random forest	100.0	4.0	0.0	46.4	36.4	37.3	2 (T1, T4)	7.4
MLP	99.3	0.0	0.0	0.0	92.7	38.3	2 (T1, T5)	5.5
1-D CNN	20.0	51.0	0.0	0.0	22.5	18.7	1 (T2)	5.0
KAN	10.0	0.0	0.7	0.0	71.5	16.4	1 (T5)	4.3

**Table 6:** Effect of the symbolic-extraction protocol on the converted-route equation, at ten random seeds on each external test set; every row is over those ten. “P0 published” fits each edge against the last mini-batch drawn and does not refit; “P3 corrected” fits each edge against the whole calibration set and then refits the converted network for 200 steps with the sparsity penalty off. The spline network is identical in both rows of a dataset; only the conversion differs. Medians are given first because on tahini a single failed extraction moves the mean by more than the median difference between protocols. The protocols were not compared by a corrected test, so the differences are descriptive. Fidelity is the R^2^ of the printed equation against the predictions of the network it was extracted from. The mango network is the compact single-hidden-layer KAN on 12 PLS scores from which the equation is read, not the 24-score accuracy variant of Table 3.

Dataset	Protocol	Network R^2^ (Med.)	Equation R^2^ (Med.)	Equation R^2^ (Mean ± SD)	Equation R^2^ Range	Runs Below 0.5	Fidelity (Med.)	Terms (Med.)
tahini	P0 published	0.977	0.952	0.859 ± 0.205	0.324 to 0.969	1	0.952	16
tahini	P3 corrected	0.977	0.988	0.884 ± 0.324	−0.038 to 0.993	1	0.985	16
mango	P0 published	0.821	0.846	0.844 ± 0.010	0.823 to 0.857	0	0.972	18
mango	P3 corrected	0.821	0.851	0.851 ± 0.010	0.838 to 0.864	0	0.969	16

**Table 7:** Edge bases that give an exact closed form (exact route), compared with the B-spline network and the corrected conversion (converted route), on both external test sets at five seeds each. Basis and regularisation were selected by grouped cross-validation inside each training partition; depth and degree were selected on tahini and carried over to mango, which is a limitation of the mango arm. “Fidelity” is the R^2^ of the printed equation, compiled and evaluated, against the predictions of the network it came from; “worst-case” is the minimum over the five seeds. “B-spline, converted” is the corrected pykan conversion run on the same inputs. The plain B-spline row is the control: it is trained identically but has no elementary closed form. Pairwise tests are in Table S14.

Dataset	Inputs	Edge Basis/Method	External R^2^ (Mean ± SD)	Equation Fidelity to the Network	Worst-Case Fidelity	Closed Form	Median Terms	Parameters
tahini	PLS scores	B-spline (control)	0.983 ± 0.008	—	—	No closed form	—	234
tahini	PLS scores	Chebyshev	0.986 ± 0.007	0.999996	0.999990	Exact	16	168
tahini	PLS scores	Fourier	0.940 ± 0.007	0.999998	0.999995	Exact	16	168
tahini	PLS scores	Powers	0.983 ± 0.006	0.999997	0.999993	Exact	16	168
tahini	PLS scores	B-spline, converted	0.987 ± 0.003	0.983045	0.972049	Approximate	14	684
tahini	PLS scores	Gaussian	0.983 ± 0.006	0.999999	0.999998	Exact	19	168
tahini	selected bands	B-spline (control)	0.984 ± 0.002	—	—	No closed form	—	234
tahini	selected bands	Chebyshev	0.975 ± 0.007	0.999998	0.999994	Exact	16	168
tahini	selected bands	Fourier	0.908 ± 0.016	0.999996	0.999991	Exact	16	168
tahini	selected bands	Powers	0.982 ± 0.003	0.999999	0.999998	Exact	16	168
tahini	selected bands	B-spline, converted	0.981 ± 0.005	0.779119	0.474373	Approximate	17	684
tahini	selected bands	Gaussian	0.984 ± 0.002	1.000000	0.999999	Exact	19	168
mango	PLS scores	B-spline (control)	0.859 ± 0.009	—	—	No closed form	—	274
mango	PLS scores	Chebyshev	0.860 ± 0.012	0.999992	0.999984	Exact	16	196
mango	PLS scores	Fourier	0.791 ± 0.014	0.999984	0.999964	Exact	16	196
mango	PLS scores	Powers	0.854 ± 0.007	0.999996	0.999989	Exact	16	196
mango	PLS scores	B-spline, converted	0.844 ± 0.007	0.953281	0.918161	Approximate	18	820
mango	PLS scores	Gaussian	0.862 ± 0.006	0.999997	0.999995	Exact	19	196
mango	selected bands	B-spline (control)	0.772 ± 0.021	—	—	No closed form	—	190
mango	selected bands	Chebyshev	0.772 ± 0.042	0.999966	0.999930	Exact	16	136
mango	selected bands	Fourier	0.684 ± 0.029	0.999977	0.999951	Exact	16	136
mango	selected bands	Powers	0.815 ± 0.022	0.999961	0.999923	Exact	16	136
mango	selected bands	B-spline, converted	0.107 ± 1.186	0.195366	−2.477754	Approximate	20	580
mango	selected bands	Gaussian	0.775 ± 0.043	0.999985	0.999976	Exact	19	136

**Table 8:** Calibrations fitted on individually selected absorbance bands (SNV-corrected) rather than on latent variables, on tahini. “Offered” is the number of channels supplied by the selection; “bands used” and “terms” are medians over the five seeds, because the retained set is not the same in every run. The upper block (“inner CV (VIP)”) uses bands ranked by PLS VIP inside the training folds; the lower block (“anchored”) uses eight bands fixed in advance from tabulated assignments. EBM is the explainable boosting machine and EBM+int the same model with pairwise interactions; GAM is a spline generalised additive model; “KAN (2,)” and “KAN (3,)” denote a hidden layer of width two and three, and “additive” the form with one univariate function per band and no hidden layer. Differences between rows were not tested.

Band Set	Offered	Model	External R^2^ (Mean ± SD, 5 Seeds)	Bands Used	Terms	Fidelity (Mean)
inner CV (VIP)	16	GAM	0.9924 ± 0.0000	16	—	—
inner CV (VIP)	16	Ridge	0.9923 ± 0.0000	16	—	—
inner CV (VIP)	16	OLS	0.9923 ± 0.0000	16	—	—
inner CV (VIP)	16	PLS	0.9918 ± 0.0000	16	—	—
inner CV (VIP)	12	Ridge	0.9910 ± 0.0000	12	—	—
inner CV (VIP)	12	PLS	0.9910 ± 0.0000	12	—	—
inner CV (VIP)	12	OLS	0.9910 ± 0.0000	12	—	—
inner CV (VIP)	16	KAN additive spline	0.9904 ± 0.0013	16	—	—
inner CV (VIP)	16	KAN additive equation	0.9895 ± 0.0009	6	7	0.9936
inner CV (VIP)	16	EBM+int	0.9891 ± 0.0005	16	—	—
inner CV (VIP)	12	KAN additive equation	0.9888 ± 0.0011	8	10	0.9924
inner CV (VIP)	16	EBM	0.9884 ± 0.0003	16	—	—
inner CV (VIP)	12	EBM+int	0.9868 ± 0.0004	12	—	—
inner CV (VIP)	16	KAN (3,) spline	0.9860 ± 0.0054	16	—	—
inner CV (VIP)	12	KAN additive spline	0.9856 ± 0.0009	12	—	—
inner CV (VIP)	12	GAM	0.9854 ± 0.0000	12	—	—
inner CV (VIP)	12	EBM	0.9844 ± 0.0002	12	—	—
inner CV (VIP)	12	KAN (2,) equation	0.9828 ± 0.0035	10	20	0.9254
inner CV (VIP)	12	KAN (3,) equation	0.9812 ± 0.0112	10	20	0.8928
inner CV (VIP)	12	KAN (2,) spline	0.9776 ± 0.0132	12	—	—
inner CV (VIP)	12	KAN (3,) spline	0.9769 ± 0.0169	12	—	—
inner CV (VIP)	16	KAN (2,) equation	0.9503 ± 0.0584	14	21	0.9241
inner CV (VIP)	16	KAN (2,) spline	0.9455 ± 0.0571	16	—	—
inner CV (VIP)	16	KAN (3,) equation	0.9034 ± 0.1836	13	26	0.8454
anchored	8	EBM+int	0.9608 ± 0.0012	8	—	—
anchored	8	GAM	0.9607 ± 0.0000	8	—	—
anchored	8	EBM	0.9560 ± 0.0011	8	—	—
anchored	8	KAN additive spline	0.9549 ± 0.0016	8	—	—
anchored	8	KAN (3,) spline	0.9449 ± 0.0098	8	—	—
anchored	8	KAN (2,) spline	0.9375 ± 0.0216	8	—	—
anchored	8	OLS	0.9360 ± 0.0000	8	—	—
anchored	8	PLS	0.9360 ± 0.0000	8	—	—
anchored	8	Ridge	0.9358 ± 0.0000	8	—	—
anchored	8	KAN (2,) equation	0.9279 ± 0.0257	7	12	0.9112
anchored	8	KAN (3,) equation	0.9263 ± 0.0264	7	17	0.9484
anchored	8	KAN additive equation	0.9162 ± 0.0314	7	11	0.9210

**Table 9:** Network, printed equation and the fidelity between them on material the model never saw. “Converted” is a B-spline network put through the corrected conversion of Section 3.3; “exact” is a KAN layer whose edge functions are already elementary, in the basis named; “OLS” is ordinary least squares on the same inputs, carried as the linear control. Each tahini production lot and each mango harvest season is withheld in turn as a fold, with three seeds for the stochastic routes; the model configuration is carried over from the primary partition (control design, Section 2.3), so absolute accuracies are optimistic (nested accuracies in Section S4). Every column is computed at the fold level—averaged over seeds inside a fold, then over folds—so that the “worst fold” columns are fold means and not single runs; the conversion returned no equation in two of the fifteen tahini latent-score runs, and a run without an equation contributes to neither column, so within a fold the network and the equation are always averaged over the same seeds. “Equation—network” is the difference in R^2^ between the printed formula and the network it represents when the inputs lie outside the calibration set. Scores are on the primary output alone and are not sum-to-100 normalised, because a printed equation yields one number; absolute values are therefore not directly comparable with Section S4, but both routes are scored identically, so the comparison between them is unaffected. No difference in this table is significant after Holm adjustment (Table S14).

Dataset	Inputs	Route	Folds	Network R^2^ (Mean)	Equation R^2^ (Mean)	Equation—Network	Worst Fold, Equation R^2^	Fidelity (Mean)	Fidelity (Worst Fold)
mango	PLS scores	converted	4	0.807	0.818	+0.011	0.771	0.943689	0.912835
mango	PLS scores	OLS	4	0.817	0.817	+0.000	0.773	1.000000	1.000000
mango	PLS scores	exact (Chebyshev)	4	0.813	0.813	+0.000	0.735	0.999994	0.999993
mango	selected bands	converted	4	0.732	0.732	+0.000	0.722	0.902573	0.781243
mango	selected bands	OLS	4	0.757	0.757	+0.000	0.704	1.000000	1.000000
mango	selected bands	exact (powers)	4	0.763	0.763	+0.000	0.739	0.999958	0.999913
tahini	PLS scores	converted	5	0.948	0.939	−0.008	0.840	0.956495	0.868291
tahini	PLS scores	OLS	5	0.939	0.939	+0.000	0.837	1.000000	1.000000
tahini	PLS scores	exact (Chebyshev)	5	0.912	0.912	−0.000	0.708	0.999994	0.999991
tahini	selected bands	converted	5	0.962	0.931	−0.030	0.844	0.935751	0.821166
tahini	selected bands	OLS	5	0.924	0.924	+0.000	0.865	1.000000	1.000000
tahini	selected bands	exact (powers)	5	0.962	0.962	+0.000	0.900	0.999992	0.999978

**Table 10:** Additional baselines on the published splits and the same latent inputs, three seeds each. “Reference perceptron” is a fixed two-hidden-layer network with batch normalisation and dropout, fitted on the same latent width as the KAN; it is a reference architecture for this comparison, not the tuned benchmark MLP of Table 3, which is why its mango score differs from that table. The transparency column states what each model yields: an additive glass-box returns a shape function per input, which can be plotted but not printed as a formula; the spline-activation perceptron applies its splines after the inputs have been mixed and so yields no closed form in the original variables. Fit times are medians over the three seeds, measured on one machine under a fixed thread cap, and are given because they differ by two orders of magnitude; only their ratios are meaningful. The comparison uses the latent scores; the same models on the full 1762-channel spectrum were not computable within this study’s computational budget and are not reported.

Dataset	Inputs	Model	Transparency	External R^2^ (Mean ± SD, 3 Seeds)	Median Fit Time (s)
tahini	PLS scores	spline-activation perceptron	Opaque (spline activations on nodes)	0.993 ± 0.001	251
tahini	PLS scores	reference perceptron	Opaque	0.988 ± 0.003	42
tahini	PLS scores	boosting machine + interactions	Additive glass-box with interactions	0.988 ± 0.001	661
tahini	PLS scores	spline additive model	Additive glass-box	0.982 ± 0.000	3
tahini	PLS scores	boosting machine	Additive glass-box	0.980 ± 0.000	68
tahini	PLS scores	gradient boosting	Opaque ensemble	0.949 ± 0.001	3
tahini	PLS scores	symbolic regression	Closed-form (genetic programming)	0.490 ± 0.333	98
mango	PLS scores	reference perceptron	Opaque	0.889 ± 0.001	995
mango	PLS scores	spline-activation perceptron	Opaque (spline activations on nodes)	0.860 ± 0.009	3916
mango	PLS scores	boosting machine + interactions	Additive glass-box with interactions	0.859 ± 0.003	605
mango	PLS scores	spline additive model	Additive glass-box	0.850 ± 0.000	76
mango	PLS scores	boosting machine	Additive glass-box	0.848 ± 0.001	72
mango	PLS scores	gradient boosting	Opaque ensemble	0.839 ± 0.006	19
mango	PLS scores	symbolic regression	Closed-form (genetic programming)	−0.134 ± 0.464	632

**Table 11:** Detection capability of this study’s spectroscopic screen compared with published confirmatory and spectroscopic methods for related matrices. Limits are as reported by each study; units differ between trace-level (mg/kg) and bulk (% *w*/*w* or *v*/*v*) methods. The limits of this study apply only to samples similar to its calibration population (Section 3.2).

Method	Analyte and Matrix	Detection or Quantification Limit	Reference
ATR–FTIR with six calibration models (this study, out of fold)	Peanut or sunflower paste in tahini	LOD 8.9% (PLS) to 21.6% (SVR); KAN 14.3%; nested leave-one-lot-out 10.3–19.5% (three leading models)	This study
ATR–FTIR with one-class model	Peanut or sunflower oil in tahini	Authenticity threshold 10.78%	[10]
^1^H NMR (marker compound)	Peanut in nut powders	≈4%	[58]
Gas chromatography with chemometrics	Vegetable oils in sesame oil	5%	[11]
Real-time PCR	Sesame in foods	0.005%	[12]
Real-time PCR; ELISA	Hazelnut in nut paste	LOQ 2 mg/kg; LOQ 1 mg/kg	[13]
LC–MS/MS (signature peptides)	Peanut in foods	LOQ 10 mg/kg	[14]

## Data Availability

The tahini ATR–FTIR dataset collected for this work is released with the paper. It comprises the raw spectra, the per-scan composition labels and the physical-sample identifiers on which the grouped validation depends, and it is distributed exactly as recorded, with no correction applied. The code that produces every result, figure and table in this article and its Supplementary Materials is released alongside it, including the supplementary analyses (folders revision/and paper1_rigor/, with the script named in each supplementary section and the result files it produces). Both are deposited at https://github.com/abdulhamidbatayhi123/kanfood-tahini-mango- (15 August 2026), the code under the MIT Licence and the data under the Creative Commons Attribution 4.0 (CC-BY-4.0) Licence, and archived at Zenodo under the concept DOI https://doi.org/10.5281/zenodo.21446757, which resolves to the most recent version. The NIR mango measurements are the public benchmark of Anderson et al. [7], obtained from Mendeley Data (https://doi.org/10.17632/46htwnp833.1), used without modification, and partitioned exactly as distributed.

## Supplementary Materials

The following supporting information can be downloaded at https://www.mdpi.com/article/10.3390/foods15203639/s1, Section S1, predicted against reference values for every model on both datasets (Figures S1 and S2); Section S2, identification of the adulterant in addition to its quantification (Table S1); Section S3, the ranking over ten independent grouped hold-outs (Table S2, Figure S3); Section S4, generalisation with a whole production lot or harvest season withheld, with nested and frozen selection (Table S3); Section S5, the single-factor ablation of every architectural and preprocessing choice (Table S4, Figure S4); Section S6, the widened preprocessing search (Table S5); Section S7, the parameter-matched capacity comparison in the three forms it was run (Table S6); Section S8, run-to-run reproducibility of the pipeline (Table S7); Section S9, the stability of the extracted equation and whether the extraction is reproducible at a fixed seed (Tables S8–S10); Section S10, the learned response curves, the band assignments and the chemical reading (Figures S5–S7, Table S11); Section S11, the network’s intrinsic variable importance against permutation importance and SHAP (Tables S12 and S13, Figure S8); Section S12, every corrected significance test in full (Tables S14 and S15); Section S13, extended methods and the detection-limit re-estimation in full; Section S14, controls for instrumental artefacts at the unassigned channels (Table S16); Section S15, the number of channels and band integration (Table S17); Section S16, low-level identification and false positives on authentic tahini (Tables S18 and S19); Section S17, the two-stage lot and sample bootstrap (Table S20); Section S18, mango residuals by ripening treatment, cultivar, temperature label and region (Table S21); Section S19, alternative input representations for mango (Table S22); Section S20, the learning curve (Table S23, Figure S9); Section S21, the training budget (Table S24); Section S22, the KAN with the favourable single factors combined (Table S25); Section S23, thirty repeated grouped hold-outs with equivalence tests (Tables S26 and S27); Section S24, paired tests of the parameter-matched comparison (Table S28); Section S25, numerical precision of the exact route, input clipping and the constants of Equation (3) (Table S29). References [42,45,46,48,49,50] of this article and four further references ([62,63,64,65]) are cited in the Supplementary Materials.
