# Supplementary material for: Interpretable Machine Learning in Food Analysis: Closed-Form Chemometric Calibration of Mid- and Near-Infrared Spectra Using Kolmogorov–Arnold Networks
Source: Foods. 2026 Oct 13;15(20):3639. doi: 10.3390/foods15203639 (PMC13704426; doi:10.3390/foods15203639)
Supplement: Supplementary file 1 [file foods-15-03639-s001.zip › foods-4561619-supplementary.pdf]

Supplementary Materials — Interpretable Machine Learning in Food Analysis: Closed-Form Chemometric Calibration of Mid- and Near-Infrared Spectra Using Kolmogorov–Arnold Networks

Abdulhamid Batayhi, Muhammed Özgölet, Hasan Cankurt, Muhammed Arici

This document contains the supporting analyses cited in the article. Each section is cited in the article at the point where its result is used, and each is reported to the same standard: every number is taken directly from a result file released with the code, no value is transcribed from a log, and the intervals and tests follow the conventions of Section 2.5 of the article. Sections, tables and figures in this document carry an S prefix and are numbered independently of the article. Numbered citations refer to the reference list of the article; citations [62]–[65] refer to the list at the end of this document.

Section S1 shows the per-spectrum predictions behind the benchmark tables. Sections S2–S5 report the validation work that establishes how far the article's headline comparison can be trusted: which adulterant each model identifies, how the ranking behaves over ten independent splits, what happens when a whole production lot or harvest season is withheld, and which design choices the result actually depends on. Section S6 widens the preprocessing search. Section S7 gives the parameter-matched capacity comparison in all three forms in which it was run, including the form that gave an incorrect result. Sections S8 and S9 measure what a repeated run and a repeated extraction actually return. Section S10 gives the learned response curves, the band assignments and the chemical reading of the equation. Section S11 compares the network's intrinsic importances with those of post-hoc explainers. Section S12 collects the corrected significance tests for every comparison introduced after Section 3.2 of the article except the parameter-matched comparison, which is tested in Section S24, and Section S13 gives extended methods. Sections S14–S23 report the analyses added in revision: controls for instrumental artefacts at the selected channels (S14), the number of channels needed (S15), low-level identification and false positives on authentic tahini (S16), a two-stage lot and sample bootstrap (S17), the mango errors by cultivar, ripening treatment and region (S18), alternative input representations for mango (S19), a learning curve (S20), the training budget (S21), the KAN with its favourable design choices combined (S22) and thirty repeated grouped hold-outs (S23). Sections S24 and S25 report paired tests of the parameter-matched comparison (S24) and the numerical precision, input clipping and constants of the exact-route equation (S25).

S1. Predicted Against Reference Values

A benchmark table reports one number per model and does not show where a model errs. Figures S1 and S2 plot the per-spectrum predictions behind Tables 2 and 3 of the article against the identity line, one panel per model.

Two features of the tahini panels (Figure S1) do not appear in the summary statistics. The high-accuracy models track the diagonal over the whole range with no systematic curvature, which is the visual counterpart of the near-linearity on which the article's argument rests: a mixing law departing from Beer–Lambert behaviour would appear as a curved point cloud rather than a straight one. Support-vector regression and the random forest instead overpredict at the low-tahini end and underpredict the authentic samples at 100%, the usual signature of a model whose predictions shrink towards its training mean. Their scatter on those authentic samples is by far the widest of the six, and that

 https://doi.org/10.3390/foods15203639

scatter is exactly the quantity from which the blank dispersion σ is estimated. This is why their hold-out detection limits are five to seven times those of PLS, the MLP and the KAN, and more than three times that of the CNN (Section 3.2); out of fold, only SVR remains separated from the other models (Table S15). The limit depends on the blank dispersion and the calibration slope, not on any offset, so the difference is driven by their scatter rather than by their underprediction. For support-vector regression, the offset is nonetheless large in its own right: on average, the model reports 12.7 percentage points of adulterant in tahini that contains none (Section S2).

The mango panels (Figure S2) show the same shrinkage acting on every model at once. All six overpredict the lowest dry-matter fruit and underpredict the highest, so the point cloud is rotated slightly relative to the identity line rather than merely scattered about it, and the effect is as visible for PLS as for the KAN. That is a distribution-shift signature rather than a defect of any one architecture: season 4 has a higher mean dry matter than the calibration seasons (17.02% against 16.18%), and a calibration fitted on the latter pulls the predicted extremes of the former towards the calibration mean. This shift explains why PLS and the random forest score below their within-calibration cross-validated values (0.854 to 0.845 and 0.804 to 0.793), whereas the other four models are unchanged or slightly higher. The shift rotates every model's cloud without necessarily reducing its R^2, because R^2 is also sensitive to the width of the target range, which differs between the two evaluations. Section 3.5 of the article directly measures what the shift costs a printed equation, as opposed to the model from which it was derived.

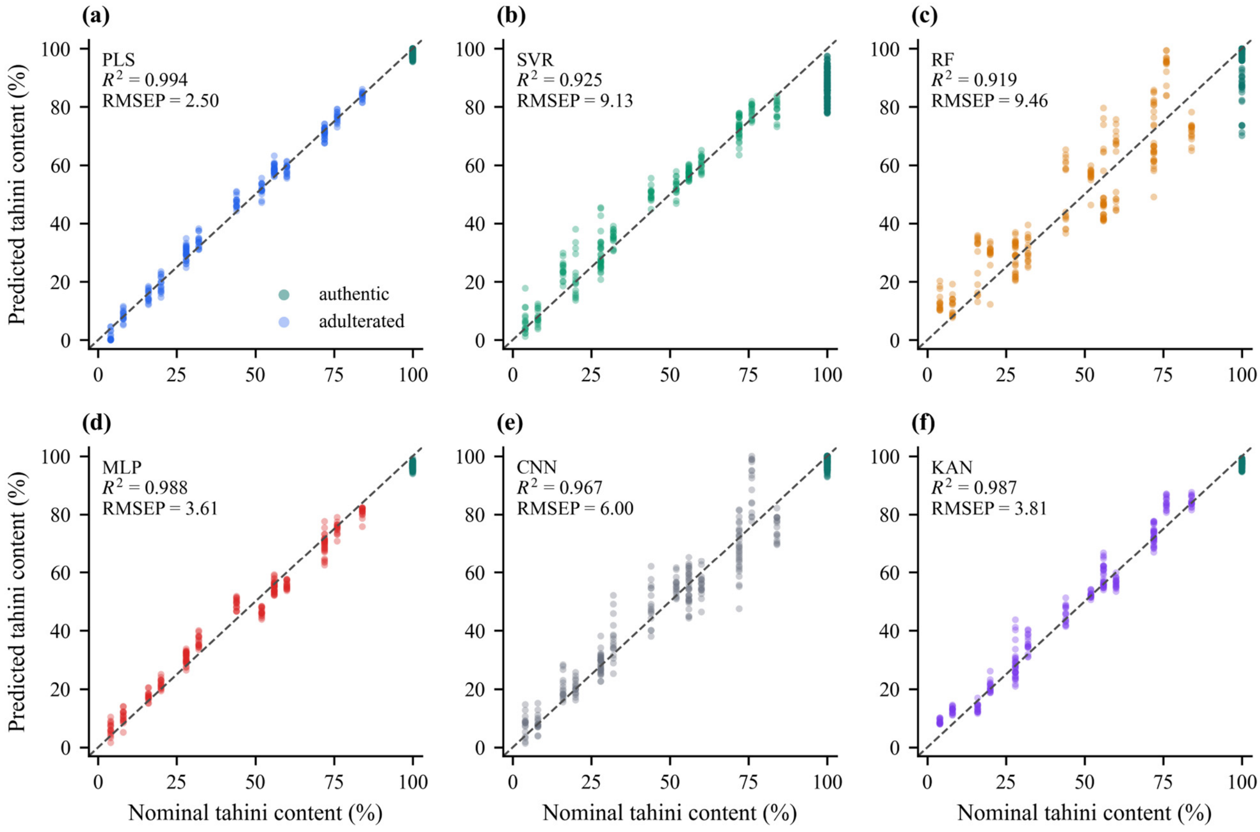

Figure S1. Tahini: predicted against nominal tahini fraction on the group-independent test set, one panel per model. The diagonal is the identity line; each point is one spectrum. Authentic samples sit at 100%.

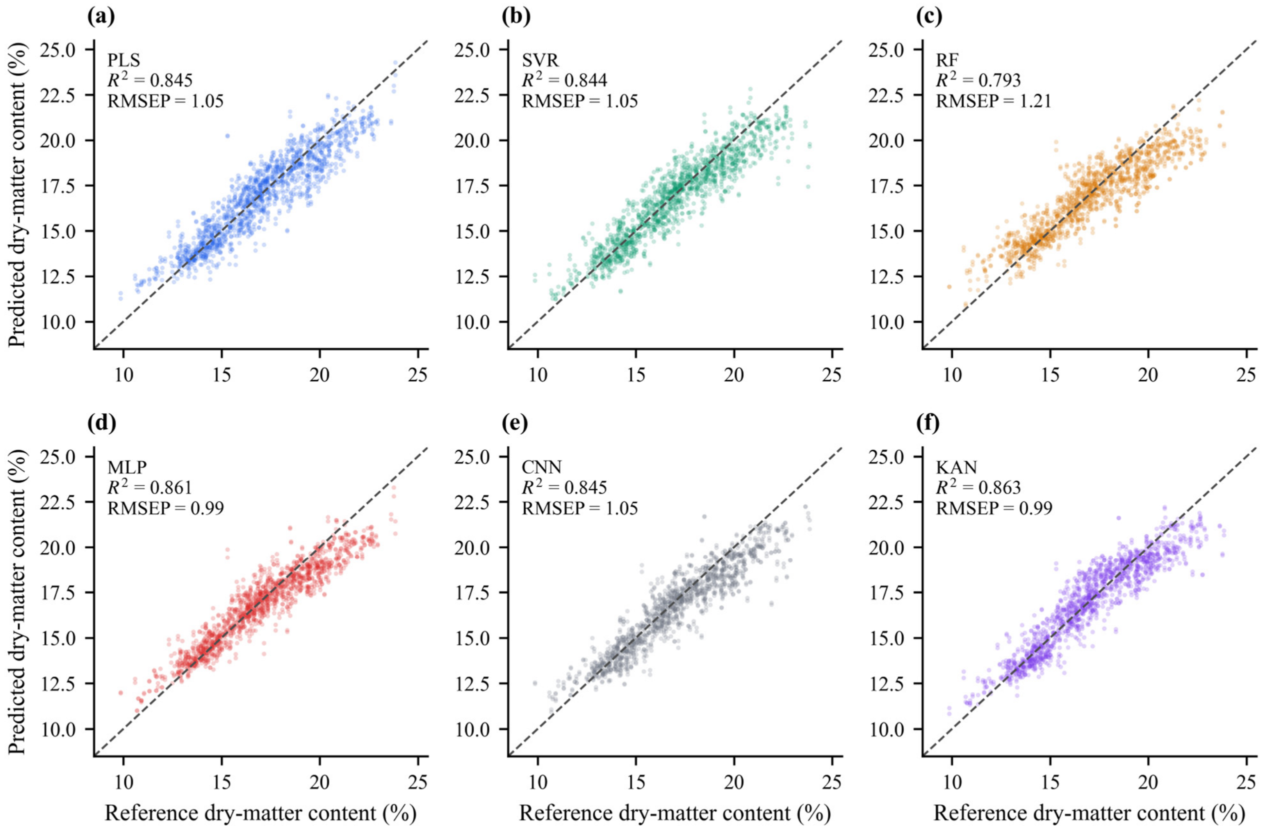

Figure S2. Mango: predicted against reference dry-matter content on the published across-season external test set, one panel per model.

S2. Identification of the Adulterant

The tahini design carries two chemically distinct adulterants, and every model predicts the full three-component composition rather than a single adulteration percentage. The same calibration can therefore identify which adulterant is present as well as quantify it, without a second model and without a classification stage (Table S1).

On the 257 adulterated spectra of the group-independent test set the KAN recovered the peanut fraction at $R^2 = 0.993$ (MAE 1.50 percentage points) and the sunflower fraction at $R^2 = 0.982$ (MAE 3.37), and identified the adulterant correctly in every spectrum (accuracy 1.00, cluster-bootstrap interval 1.00–1.00). PLS was the most accurate on the two adulterant fractions (0.998 and 0.995, MAE 1.01 and 1.66), with the MLP (0.996, 0.989) and the CNN (0.988, 0.965) close behind; the random forest was the weakest on both (0.947 and 0.932). Identification was perfect for every model except the random forest, which misassigned 12 of 257 spectra (accuracy 0.953, interval 0.852–1.000, macro-F1 0.953).

A second quantity, not captured by the fractions above, is the adulterant fraction each model reports in authentic tahini. Averaged over the 151 authentic spectra of the same partition, the value ranges from +1.44 percentage points for the convolutional network and +2.18 for PLS to +12.67 for support-vector regression; that is, SVR assigns about one-eighth of an authentic sample to adulterant. This is the same weakness that gives SVR a detection limit of 19.6% in Section 3.2 of the article, and it is invisible in an accuracy figure computed on adulterated material alone. The KAN reports +2.51.

Identification held across the whole concentration range present in the test partition: at each of the thirteen adulteration levels represented there (16–96% adulterant), the KAN assigned the correct adulterant to every spectrum. The qualification is that the grouped hold-out contains no blend below 16% adulterant, so this result concerns identification at moderate-to-gross adulteration and does not address low adulteration levels. That end of

the range is addressed by the detection limit in Section 3.2 of the article, and Section S16 tests 4–12% blends directly.

The basis for this capability is already visible in Figure 2b of the article, where the two adulterants perturb the fingerprint region in opposite directions. What matters here is that this information is retained through the whole chain (preprocessing, projection and calibration) and remains sufficient to distinguish the two adulterants in an individual spectrum. The companion mid-infrared study could not demonstrate this capability, because each of its three matrices contained a single adulterant [42].

Table S1. Identifying the adulterant as well as quantifying it: accuracy of the peanut and sunflower fractions, and of the resulting identification, on the 257 adulterated spectra of the group-independent tahini test set. The column headed "Adulterant predicted on authentic (pp)" is scored on the 151 authentic spectra of the same partition instead: it is the total adulterant fraction that each model reports in material containing none, so its true value is zero. pp, percentage points.

Model	R^2 peanut fraction	R^2 sunflower fraction	MAE peanut (pp)	MAE sunflower (pp)	Adulterant predicted on authentic (pp)	Identification accuracy [95% CI]	Macro-F1
PLS	0.998	0.995	1.01	1.66	+2.18	1.000 [1.000, 1.000]	1.000
SVR	0.973	0.985	2.93	3.09	+12.67	1.000 [1.000, 1.000]	1.000
Random forest	0.947	0.932	4.43	5.70	+3.23	0.953 [0.852, 1.000]	0.953
MLP	0.996	0.989	1.58	2.63	+3.31	1.000 [1.000, 1.000]	1.000
1-D CNN	0.988	0.965	1.95	3.76	+1.44	1.000 [1.000, 1.000]	1.000
KAN	0.993	0.982	1.50	3.37	+2.51	1.000 [1.000, 1.000]	1.000

S3. Sensitivity of the Ranking to the Split (Configuration Carried Over)

A single grouped hold-out can favour or penalise a model by chance, particularly when only 55 physical samples are available to split. The entire tahini comparison was therefore repeated over ten independent grouped hold-outs, each drawn with a different random partition of the samples (Table S2, Figure S3a). One element was held fixed: preprocessing and the PLS projection were refitted inside each split, but every model was refitted at the hyperparameter configuration selected on the primary training partition rather than re-tuned for each split. Between eight and fifteen of each split's seventeen test samples lie inside that primary training partition. This section therefore measures the sensitivity of the comparison to the split, not to the tuning, and its absolute values should not be read as clean held-out scores.

The ordering of the headline table was preserved, except for one exchange between adjacent models. PLS was both the most accurate and the most stable (R^2 = 0.989 ± 0.006 across the ten splits, range 0.980–0.997), followed by the KAN (0.967 ± 0.028, range 0.898–0.991), the MLP (0.959 ± 0.053, 0.826–0.993), the CNN (0.943 ± 0.025, 0.886–0.973), support-vector regression (0.916 ± 0.066, 0.777–0.970) and the random forest (0.863 ± 0.083, 0.711–0.952). The exchange is between the KAN and the perceptron, which the published hold-out separates by 0.001 in the perceptron's favour: over the ten splits the perceptron is the higher of the two in six, and the KAN's higher mean rests on two splits at which the perceptron fell to 0.826 and 0.907. By median, the published order is restored (0.981 against 0.977). No conclusion is drawn from this exchange: the pair was not tested over splits and both differences are far below the split-to-split spread.

https://doi.org/10.3390/foods15203639

Two observations follow. First, the near-linearity of the tahini calibration is confirmed from a different direction: the linear model is not merely the most accurate but also, by a wide margin, the least variable, which is what one expects when the underlying mixing law is close to linear and the remaining variation is sampling noise. Second, the KAN was less variable than the MLP across splits (SD 0.028 against 0.053) at a comparable mean (0.967 against 0.959), and its worst split (0.898) was better than the MLP's worst (0.826). We report this as an observation rather than a claim: the two distributions overlap heavily, ten splits is a small sample for comparing dispersions, and no test of the variance difference was performed. With the model selection repeated inside each split (Section S23), the KAN ranked fourth and had the largest spread of the four leading models, so this observation does not hold under nesting.

Table S2. Tahini: distribution of test performance over ten independent grouped hold-outs.

Model	Mean R^2 over ten grouped hold-outs	SD	Minimum	Maximum
PLS	0.989	0.006	0.980	0.997
SVR	0.916	0.066	0.777	0.970
Random forest	0.863	0.083	0.711	0.952
MLP	0.959	0.053	0.826	0.993
1-D CNN	0.943	0.025	0.886	0.973
KAN	0.967	0.028	0.898	0.991

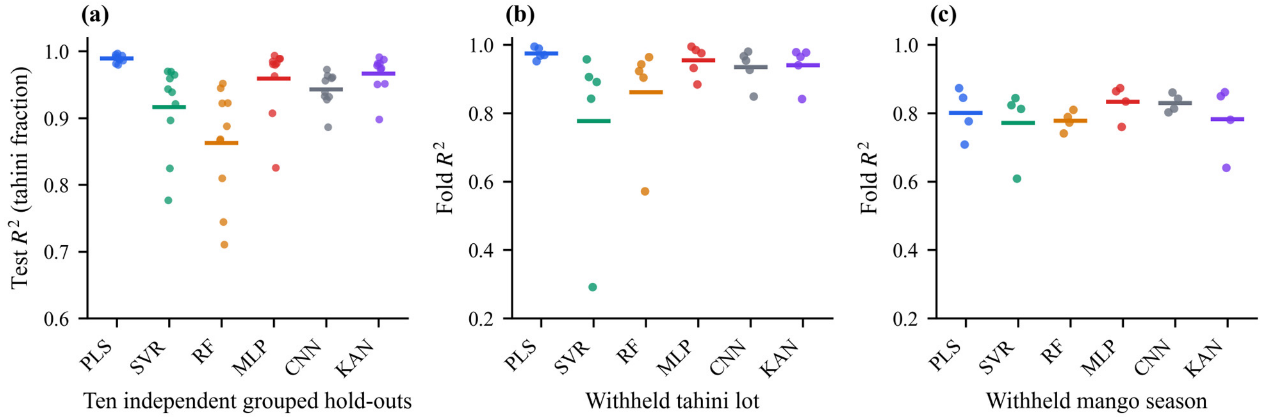

Figure S3. Validation robustness. (a) Test performance of each model over ten independent grouped hold-outs of the tahini samples; (b) performance per fold when a whole tahini production lot is withheld; (c) performance per fold when a whole mango harvest season is withheld. In all three panels the configuration selected on the primary partition is carried over rather than re-selected (frozen design; the nested results are in Table S3), and the mango SVR folds are those of the rerun (Section S8). Points are individual splits or folds; horizontal bars are means. Panel (a) is discussed in this section, and panels (b) and (c) in Section S4.

S4. Generalisation Beyond the Split: A Whole Lot, and a Whole Season, Withheld

Grouped hold-outs test whether a model generalises to unseen *samples* drawn from the same pool. They do not test whether it generalises to a source of variation absent from that pool altogether. This limitation was the criticism most often raised against the companion study, and it is addressed here directly by withholding an entire tahini production lot, and an entire mango harvest season, at a time (Table S3, Figure S3b,c). Section 3.5 of the article uses the same folds to measure how the printed equation behaves off-distribution.

https://doi.org/10.3390/foods15203639

Tahini, leave-one-lot-out, frozen configuration. Each of the five authentic tahini lots was withheld in turn together with every blend prepared from it, so that the model was calibrated on four production lots and tested on a fifth it had never seen — roughly 310 spectra per fold. Performance fell for every model relative to the grouped hold-out, and the ordering changed. PLS was most robust (mean R^2 across the five folds 0.975, SD 0.017, worst fold 0.952), followed by the MLP (0.954, SD 0.046, worst 0.884), the KAN (0.940, SD 0.057, worst 0.841) and the CNN (0.935, SD 0.052, worst 0.848); the random forest (0.861, SD 0.163) and support-vector regression (0.778, SD 0.275, worst fold 0.291) degraded severely. One lot, T1, was the hardest fold for every non-linear model except the convolutional network, whose hardest lot was T2 (0.848). On T1, the KAN fell to 0.841 and the MLP to 0.884, whereas PLS held 0.969.

Mango, leave-one-season-out, frozen configuration. Rotating the withheld harvest season through all four seasons gives a harder and more variable test than the published season-4 split alone, because seasons 1–3 differ in size and in cultivar composition. The MLP was best on average (0.833, SD 0.052, worst 0.760), then the CNN (0.830, SD 0.026, worst 0.803), PLS (0.801, SD 0.074, worst 0.708), the KAN (0.783, SD 0.102, worst 0.640), the random forest (0.778, SD 0.029) and support-vector regression (0.772, SD 0.110, worst 0.609; rerun, Section S8).

These results are the least favourable in the study and are reported in full. They support three statements. First, transfer to an unseen production lot or harvest season costs every model accuracy, and no model in this comparison should be described as season- or lot-independent. Second, on both datasets the KAN's fold-to-fold variability under this test is larger than that of the best model — SD 0.057 against 0.017 for PLS on tahini, and 0.102 against 0.052 for the MLP on mango — so the interpretable model is not the most dependable one when the calibration is asked to extrapolate to a new source of material. Third, on tahini the two models that collapse are support-vector regression and the random forest, whose worst folds (0.291 and 0.572) place them outside a useful range. On mango, only support-vector regression falls outside a useful range (worst fold 0.609), whereas the random forest is intermediate there (mean 0.778, worst fold 0.741, the second-smallest fold-to-fold spread) and, together with the convolutional network, shows the smallest loss relative to its published season-4 score (0.015 each). As in Section S3, the hyperparameters here are carried over from the primary partitions rather than re-selected inside each fold. On mango, that partition comprises all of seasons 1–3, so for three of the four withheld seasons the configuration was selected with material from the withheld season visible. These folds test transfer at a fixed configuration, which is a weaker test than nested selection.

Nested selection. Because the frozen design uses a configuration selected with material from the withheld lot or season, all selection was repeated inside each fold (Table S3, rows labelled "nested"). Compression and hyperparameters were chosen by an inner leave-one-lot-out (tahini) or leave-one-season-out (mango) loop on the training lots or seasons only, with the published preprocessing; in a second tahini analysis the preprocessing (SNV, SNV followed by a first-derivative filter, the first-derivative filter alone, MSC, or no preprocessing) was selected in the same inner loop. On tahini, nesting raised the KAN's mean R^2 over the five lots from 0.940 to 0.968 and its worst lot from 0.841 to 0.948, and lowered those of PLS from 0.975 to 0.950 and from 0.952 to 0.837; the MLP (0.959, worst 0.896) and the CNN (0.941, worst 0.857) changed little. With the preprocessing also selected, the KAN reached 0.976 (worst 0.953), SVR 0.964 (0.935) and RF 0.966 (0.906); for these models, the inner loop chose the first-derivative filter in most folds, and it never selected unprocessed spectra for any model. PLS retained SNV in four of the five folds and did not gain (0.948, worst 0.837). On mango, nesting raised the KAN's mean from 0.783 to 0.803 and its worst season from 0.640 to 0.752, and left the mean R^2 of the other

https://doi.org/10.3390/foods15203639

models within 0.006 of the frozen design (worst season within 0.011); the CNN (0.829, worst 0.804) and the MLP (0.827, worst 0.752) had the highest means. The KAN's fold-to-fold variability under the frozen design is therefore largely a property of the carried-over configuration: with nested selection its SD over lots was 0.017 (PLS 0.065) and over seasons 0.051 (PLS 0.062). In the lot-level bootstrap of Section S17, the KAN did not differ significantly from PLS, the MLP or the CNN (p = 1.000), although it did differ from RF and SVR; the mango season results were not tested. The ordering of the leading models is therefore a ranking of point estimates, and every model still lost accuracy on a withheld lot or season relative to the grouped hold-out.

The practical reading is that a calibration of this kind is a screening tool for material resembling what it was trained on, and that recalibration or standardisation would be required before applying it to a new production lot or a new season. The companion study reached the same conclusion for brands [42].

Table S3. Generalisation beyond the split. For tahini each fold withholds one of the five authentic production lots together with every blend prepared from it; for mango each fold withholds one of the four harvest seasons, so the Fold 5 column is empty in the mango rows. Stochastic models are averaged over three seeds per fold, and "Worst" is the lowest fold. Rows labelled "tahini" and "mango" carry the configuration selected on the primary partition into every fold (frozen design, as originally reported; the mango SVR row is the rerun of Section S8). Rows labelled "nested" select compression and hyperparameters, and "nested + preprocessing" also the preprocessing, by an inner leave-one-lot-out or leave-one-season-out loop inside each training set (script revision/r01_nested_generalisation.py); the CNN was not included in the preprocessing search.

Dataset	Model	Fold 1	Fold 2	Fold 3	Fold 4	Fold 5	Mean	Median	Worst
tahini	PLS	0.969	0.970	0.994	0.989	0.952	0.975	0.970	0.952
tahini	SVR	0.291	0.892	0.957	0.842	0.906	0.778	0.892	0.291
tahini	Random forest	0.572	0.963	0.923	0.904	0.943	0.861	0.923	0.572
tahini	MLP	0.884	0.976	0.994	0.985	0.933	0.954	0.976	0.884
tahini	1-D CNN	0.927	0.848	0.966	0.980	0.953	0.935	0.953	0.848
tahini	KAN	0.841	0.977	0.979	0.964	0.941	0.940	0.964	0.841
mango	PLS	0.708	0.777	0.873	0.845		0.801	0.811	0.708
mango	SVR	0.609	0.813	0.823	0.844		0.772	0.818	0.609
mango	Random forest	0.773	0.810	0.741	0.789		0.778	0.781	0.741
mango	MLP	0.760	0.835	0.864	0.873		0.833	0.849	0.760
mango	1-D CNN	0.814	0.842	0.803	0.861		0.830	0.828	0.803
mango	KAN	0.640	0.781	0.849	0.862		0.783	0.815	0.640
tahini, nested	PLS	0.837	0.981	0.992	0.988	0.952	0.950	0.981	0.837
tahini, nested	SVR	0.307	0.880	0.958	0.869	0.916	0.786	0.880	0.307
tahini, nested	Random forest	0.590	0.963	0.922	0.907	0.942	0.865	0.922	0.590
tahini, nested	MLP	0.896	0.978	0.994	0.987	0.941	0.959	0.978	0.896
tahini, nested	1-D CNN	0.928	0.857	0.965	0.986	0.966	0.941	0.965	0.857
tahini, nested	KAN	0.953	0.977	0.986	0.977	0.948	0.968	0.977	0.948
tahini, nested + preprocessing	PLS	0.837	0.981	0.981	0.988	0.952	0.948	0.981	0.837

Dataset	Model	Fold 1	Fold 2	Fold 3	Fold 4	Fold 5	Mean	Median	Worst
tahini, nested + prepro-cessing	SVR	0.936	0.979	0.978	0.990	0.935	0.964	0.978	0.935
tahini, nested + prepro-cessing	Random forest	0.906	0.989	0.984	0.984	0.969	0.966	0.984	0.906
tahini, nested + prepro-cessing	MLP	0.896	0.977	0.992	0.988	0.943	0.959	0.977	0.896
tahini, nested + prepro-cessing	KAN	0.967	0.993	0.990	0.978	0.953	0.976	0.978	0.953
mango, nested	PLS	0.719	0.806	0.858	0.838		0.805	0.822	0.719
mango, nested	SVR	0.609	0.813	0.823	0.844		0.772	0.818	0.609
mango, nested	Random forest	0.772	0.809	0.742	0.793		0.779	0.783	0.742
mango, nested	MLP	0.752	0.837	0.858	0.860		0.827	0.847	0.752
mango, nested	1-D CNN	0.812	0.842	0.804	0.857		0.829	0.827	0.804
mango, nested	KAN	0.752	0.769	0.861	0.829		0.803	0.799	0.752

S5. Single-Factor Ablation

The grid, the spline order, the hidden width, the activation penalty and the preprocessing family were each varied one factor at a time from the published configuration, with the number of latent scores, the filter width, the optimiser settings, the step budget and the pruning thresholds held at their published values; each variant was scored by grouped five-fold cross-validation inside the training partition (Table S4, Figure S4). On tahini, the result is unfavourable to the published configuration and is reported as obtained.

The published tahini configuration is not optimal. Its cross-validated R^2 was 0.942, and eight single-factor variants exceeded it: SNV followed by a first-derivative Savitzky–Golay filter (+0.040), a three-interval spline grid (+0.034), a first derivative alone (+0.034), spline order four (+0.032), hidden width five (+0.032), hidden width four (+0.023), spline order two (+0.019) and removing the activation penalty altogether (+0.008). The configuration was not re-tuned, because the headline results were produced at the published configuration and are therefore, if anything, conservative. Section S6 reports the results obtained when the preprocessing search is properly widened and the selection is made inside the training partition.

Mango is insensitive to architecture. Every architectural variant lay within ±0.007 R^2 of the baseline 0.841 — grid size from 3 to 20, spline order 2 to 4, hidden width from (3,) to (32, 16). Only preprocessing and regularisation moved the result at all. With 94 calibration populations and 10,243 spectra, there is enough data to fit any of these configurations; this is precisely what distinguishes mango from tahini, which has 38 training samples.

The regularisation penalty is the most sensitive parameter, and its usable range is narrow. The activation penalty $\lambda = 10^{-3}$ used throughout lies close to the upper end of this range, which ends abruptly. On tahini, $\lambda = 0.01$ cost 0.021 R^2 and $\lambda = 0.1$ caused the model to fail ($R^2 = -0.234$, SD 0.352). On mango, the collapse occurred at a penalty ten times smaller: $\lambda = 0.01$ gave 0.502 (SD 0.416) and $\lambda = 0.1$ gave −0.011. This is the most important practical finding of the ablation for anyone reproducing this work.

A fine grid set at initialisation severely degraded the model on the small dataset but not on the large one. Initialising at grid 20 rather than 5 cost tahini 0.226 R^2 (0.716, SD 0.194) but mango only 0.004 (0.836). The difference reflects 38 training samples for tahini against 94 populations for mango: a twenty-interval spline on each of eight inputs has more free parameters than the tahini training set can constrain. This result concerns initialisation, not fine grids as such: the library's own route to a fine grid is progressive refinement from a coarse one, which we did not test, so the result should not be generalised to fine grids reached in that way.

Do the conclusions depend on the published configuration? Because eight single-factor variants exceeded the published tahini configuration, the favourable factors were combined (SNV followed by a first-derivative filter, grid 3, spline order 4, hidden width 5), and the combined configuration, held fixed, was evaluated on the external test set and with each production lot withheld (Section S22, Table S25). It reached a higher external R^2 than the published configuration and a higher mean and worst-lot R^2 with lots withheld, but not a higher external R^2 than PLS. The conclusion that the architecture offers no accuracy advantage therefore holds for the better configuration as well. Part of the lower stability of the published configuration across withheld lots in the frozen design appears to be a property of that configuration rather than of the architecture (differences not tested; the withheld-lot values are optimistic, Section S22). The equation-extraction results of the article were obtained at the published configuration and are unchanged.

Table S4. Single-factor ablation of the KAN's grid, spline order, hidden width and activation penalty, and of the preprocessing family, each varied alone from the published configuration and scored by grouped five-fold cross-validation inside the training partition. The number of latent scores, the filter width, the optimiser settings, the step budget and the pruning thresholds are held at their published values.

Dataset	Configuration	CV R^2 (mean ± SD)	Δ vs published
tahini	Published	0.942 ± 0.031	—
tahini	Grid G = 3	0.976 ± 0.021	+0.034
tahini	Grid G = 10	0.938 ± 0.031	−0.004
tahini	Grid G = 20	0.716 ± 0.194	−0.226
tahini	Spline order k = 2	0.961 ± 0.032	+0.019
tahini	Spline order k = 4	0.975 ± 0.008	+0.032
tahini	Hidden width (2,)	0.930 ± 0.069	−0.013
tahini	Hidden width (4,)	0.965 ± 0.018	+0.023
tahini	Hidden width (5,)	0.974 ± 0.010	+0.032
tahini	$\lambda = 0$	0.950 ± 0.032	+0.008
tahini	$\lambda = 0.01$	0.921 ± 0.023	−0.021
tahini	$\lambda = 0.1$	−0.234 ± 0.352	−1.176
tahini	SG first derivative	0.976 ± 0.026	+0.034
tahini	SNV + SG first derivative	0.982 ± 0.008	+0.040
tahini	MSC	0.936 ± 0.036	−0.006
mango	Published	0.841 ± 0.030	—
mango	Grid G = 3	0.841 ± 0.042	+0.000
mango	Grid G = 10	0.838 ± 0.033	−0.003
mango	Grid G = 20	0.836 ± 0.040	−0.004
mango	Spline order k = 2	0.841 ± 0.037	+0.000

Dataset	Configuration	CV R² (mean ± SD)	Δ vs published
mango	Spline order k = 4	0.845 ± 0.026	+0.004
mango	Hidden width (3,)	0.846 ± 0.039	+0.005
mango	Hidden width (8, 4)	0.842 ± 0.034	+0.002
mango	Hidden width (32, 16)	0.841 ± 0.036	+0.001
mango	λ = 0	0.843 ± 0.029	+0.003
mango	λ = 0.01	0.502 ± 0.416	−0.339
mango	λ = 0.1	−0.011 ± 0.009	−0.852
mango	SNV	0.845 ± 0.029	+0.004
mango	SNV + SG first derivative	0.848 ± 0.027	+0.007
mango	MSC	0.826 ± 0.043	−0.014

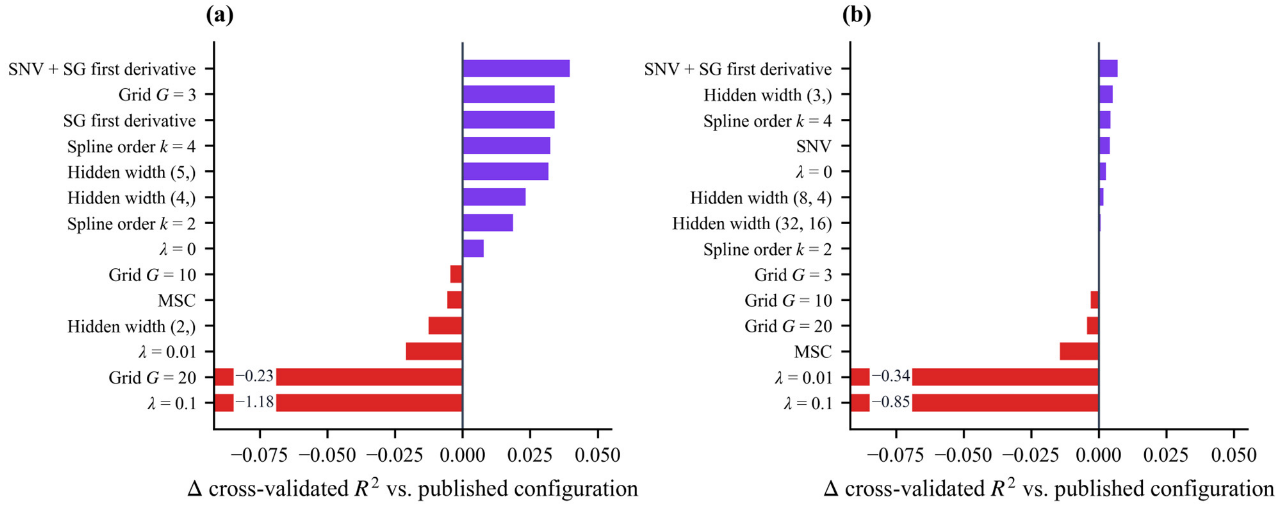

Figure S4. Single-factor ablation, expressed as the change in cross-validated performance relative to the published configuration. (a) Tahini; (b) mango. G is the number of spline-grid intervals, k the spline order and λ the activation penalty; bars extending beyond the axis are annotated with their true value.

S6. Preprocessing: Widening the Search

The preprocessing used for the headline results — the standard normal variate transformation for tahini, a first-derivative Savitzky–Golay filter of window 11 for mango — was chosen from a narrow grid that contained neither a second derivative nor any filter width other than eleven points. This section examines whether a better choice was available and whether the model comparison would be unchanged under it, by repeating the choice over a deliberately wide grid; the selection was made inside the training partition, and the external test set was used once (Table S5).

The grid. Eleven scatter-correction and derivative combinations — raw, SNV, MSC, first and second Savitzky–Golay derivatives, each derivative preceded by SNV or MSC, and each derivative followed by SNV — were considered, and the eight derivative forms were crossed with four filter widths (7, 11, 15 and 21 points) at second-order polynomial fitting, giving thirty-five configurations. Each was scored by grouped inner cross-validation on the training partition with PLS over nine latent dimensionalities (4–30). The best three configurations were then re-scored with the three expensive models, still inside the training partition, and one was carried forward to the external test for comparison with the published configuration. The configuration carried forward was chosen by the single highest inner-fold score across that set rather than by the highest fold-averaged score. This rule is a weak selector, and it is stated here because it changed which configuration was carried forward: by the cross-validated mean, the three shortlisted configurations rank in

the order MSC at window 11 (0.9931), SNV at window 11 (0.9929) and SNV + first derivative at window 21 (0.9887), so the fold-maximum rule carried the last of the three.

On tahini the published configuration is already near the top of the widened grid. Over the thirty-five configurations, ranked by their best cross-validated PLS score, the published SNV at window 11 comes second, 0.0002 behind MSC at the same width; the search finds no materially better transform. The configuration the fold-maximum rule carried to the external test, SNV followed by a first-derivative filter of width 21, changes the external results slightly and not in a consistent direction: PLS improves from 0.9943 to 0.9954, the multilayer perceptron from 0.9872 to 0.9901, and the KAN moves from 0.9835 to 0.9829 — that is, the widened search slightly *worsens* the model it was expected to help. The largest movement anywhere in the tahini comparison is 0.003 R^2 — twice the 0.001 that separates the perceptron and the KAN in Table 2 of the article, half the 0.006 that separates PLS from the perceptron, and far below the 0.02 to 0.07 that separates the leading group from the convolutional network, support-vector regression and the random forest. It is also not uniformly within the seed spread: the KAN's own movement, 0.0006, is well within both of its seed standard deviations, whereas the perceptron's 0.0029 is one to three times its own (0.0022 and 0.0010).

On mango, the search returns the published configuration, which is the cleaner outcome. Over the same eleven combinations and four widths, the configuration the search carries to the external stage is the published one — a first-derivative Savitzky–Golay filter of window 11 at second-order fitting — so the selected and published rows of Table S5 are identical by construction: PLS 0.8452, the KAN 0.8619 at 24 latent variables and 0.8287 at the 12 of the equation-bearing configuration, and the perceptron 0.8733. The selection rule identifies each model's own best configuration inside the training partition and then adopts the configuration of the best-scoring model, which here is the perceptron at the published filter (best inner fold 0.8791, fold mean 0.8648). The models did not agree on the preferred preprocessing: PLS and the compact KAN both scored highest on the *raw* spectrum, on the same best-inner-fold measure (PLS 0.8738 against 0.8723 filtered, the compact KAN 0.8665 against 0.8567), so, on this matrix, scatter correction and differentiation contribute very little, and the published choice is supported by the strongest model rather than by all of them. The published choice on the harder matrix is therefore not an artefact of the narrow grid, but neither does it have a large effect.

Three conclusions follow. The published preprocessing choice is not a weakness of the study, and no conclusion in the article depends on it: the model ordering is unchanged on both matrices and no magnitude moves by more than 0.003 R^2. On mango the search confirms the published choice outright. Finally, the selection step is the weakest element of this experiment, for two reasons: the rule we used selects on a single inner fold, and the configuration it selected is the better one externally for two of the three models and the worse one for the third. This is the same limited-power pattern reported in Section 3.6 of the article, where the KAN's own inner search also selected a configuration that scored worse on the external set than the published one. A preprocessing search of this kind is worth running to establish that no large improvement was missed, but it should not be treated as a tuning step whose output is to be adopted.

Table S5. External performance under the preprocessing configuration selected by a widened grid search inside the training partition, against the published configuration, for the three models carried to the external stage. Stochastic models are averaged over three seeds (42, 7 and 2024) and the standard deviation is computed over those seeds, a narrower budget than the five seeds of Table S6; PLS is deterministic. The mango rows are identical in the two blocks because the widened search re-selected the published configuration there; they are repeated rather than merged so that the two matrices are presented in the same way. "SG window" is the Savitzky–Golay filter width in points, at second-order polynomial fitting.

Dataset	Configuration	Preprocessing	SG window	Model	Latent variables	External R^2 (mean ± SD)
tahini	selected	SNV + SG first derivative	21	PLS	12	0.9954 ± 0.0000
tahini	selected	SNV + SG first derivative	21	KAN	8	0.9829 ± 0.0027
tahini	selected	SNV + SG first derivative	21	MLP	8	0.9901 ± 0.0022
tahini	published	SNV	11	PLS	12	0.9943 ± 0.0000
tahini	published	SNV	11	KAN	8	0.9835 ± 0.0056
tahini	published	SNV	11	MLP	8	0.9872 ± 0.0010
mango	selected	SG first derivative	11	PLS	24	0.8452 ± 0.0000
mango	selected	SG first derivative	11	KAN	24	0.8619 ± 0.0090
mango	selected	SG first derivative	11	KAN	12	0.8287 ± 0.0140
mango	selected	SG first derivative	11	MLP	16	0.8733 ± 0.0079
mango	published	SG first derivative	11	PLS	24	0.8452 ± 0.0000
mango	published	SG first derivative	11	KAN	24	0.8619 ± 0.0090
mango	published	SG first derivative	11	KAN	12	0.8287 ± 0.0140
mango	published	SG first derivative	11	MLP	16	0.8733 ± 0.0079

S7. The Parameter-Matched Comparison, in the Three Forms It Was Run

Section 3.6 of the article states the conclusion: at a matched parameter budget, the MLP had a higher mean R^2 than the KAN in all twelve configurations compared, by margins from 0.0005 to 0.061 R^2 on tahini and mango; paired by seed, the differences were not significant on tahini and were significant in five of the eight mango comparisons by a simple paired t-test, but in none after the Nadeau–Bengio correction (Section S24). This section reports the sequence of comparisons that produced it, including the one that gave an incorrect result, because the conclusion depends on the protocol (Table S6).

First attempt: matched parameters, unmatched training. When a multilayer perceptron was matched to the KAN's parameter count and both were trained with the published recipe, the KAN scored lower on both datasets (Table S6, block 1). That comparison, however, gave the perceptron 3600 gradient updates on tahini and 32,200 on mango against the KAN's 300 — twelve and one hundred and seven times more — together with batch normalisation, dropout and weight decay that the KAN never received, while the KAN was fixed at a configuration the ablation of Section S5 shows is not optimal. A difference measured under those conditions reflects the unequal training budgets.

Second attempt: matched parameters, matched convergence. We therefore matched the parameter count, trained *both* architectures to convergence by early stopping on a grouped validation split drawn from the training partition, searched both over exactly six configurations with selection by inner cross-validation on the training partition alone, and ran five seeds (Table S6, block 2). The training budgets became comparable — a median of 2000 updates for the KAN against 1600 for the perceptron on tahini — and the perceptron was still ahead on tahini (0.991 ± 0.004 against 0.949 ± 0.046) and on the compact mango configuration, and within 0.005 of the KAN on the larger mango configuration (0.876 against 0.871; not tested).

https://doi.org/10.3390/foods15203639

That conclusion was incorrect. Three things had been changed at once, not two: which configuration each architecture used, how it was trained, and — we discovered this only on re-examination — how its predictions were scored. The third turned out to be decisive on tahini, and it is described here because the error is easy to make and difficult to detect.

Every tahini model predicts a three-component composition, and the published pipeline normalises those three predictions to sum to 100% before scoring, inside the model wrapper itself. The second attempt bypassed that wrapper and scored the raw output. Training one network and scoring it both ways quantifies the cost of that omission: at seed 42, a single KAN trained for 1000 updates scores R^2 = 0.9525 unnormalised and 0.9702 normalised — reproducing, from the same fitted model, both the figure the second attempt reported and the figure the third attempt reports. The omission affects the two models unequally. Across the five seeds it moves the KAN by up to 0.039 and the perceptron by at most 0.006, because the KAN's three raw outputs depart further from summing to 100 than the perceptron's. The second attempt therefore compared the two architectures under a metric that penalised one of them, and the gap it found on tahini is largely attributable to that penalty.

Separating the three factors. Block 3 of Table S6 crosses the two configurations with the two training rules, so that the effect of each factor can be read separately. On tahini at 684 and 689 trainable parameters, the fixed-step recipe gives the KAN R^2 = 0.9859 ± 0.0061 at its published configuration and the perceptron 0.9864 ± 0.0027, while under the early-stopping harness the same KAN falls to 0.9653 ± 0.0408 and the perceptron rises to 0.9887 ± 0.0032. The harness therefore matters, and it affects the two models differently: it improves the perceptron by 0.002 and *costs* the KAN 0.021 while multiplying its seed-to-seed standard deviation by nearly seven. Its effect is nevertheless smaller than that of the scoring, and the tahini deficit reported by the second attempt does not persist under either correction.

The comparison is not symmetric, and the asymmetry favours the KAN. The KAN has a published configuration, taken from the benchmark of Section 3.2; the parameter-matched perceptron was constructed for this comparison and has none, so its only configuration is the one the six-way inner search selected. Pairing the KAN's published configuration with that selection gives the 0.0005 difference reported above, and this is the pairing most favourable to the KAN. The symmetric alternative is to compare both at their own inner-CV selections; the perceptron then leads on tahini by 0.012 under the fixed-step recipe (0.9745 ± 0.0092 against 0.9864 ± 0.0027) and by 0.020 under early stopping (0.9690 ± 0.0341 against 0.9887 ± 0.0032). The gap is not due to the architecture: it arises because the KAN's own inner search selects a configuration worse than its published one, which is the self-tuning weakness reported in Section 3.6. Either way, the conclusion is the same, and we state it in its stronger form: across all twelve cells of block 3 the perceptron had a higher mean R^2 than the KAN (paired tests in Section S24), by margins from 0.0005 to 0.061.

On mango, the perceptron is better at both capacities, but the margin depends on the harness. Under the fixed-step recipe it leads by 0.024 at the larger capacity (0.8969 ± 0.0073 against 0.8730 ± 0.0138) and 0.061 at the compact one (0.8732 ± 0.0047 against 0.8117 ± 0.0199); under early stopping, which equalises the training budget, those margins fall to 0.003 (0.8759 ± 0.0182 against 0.8729 ± 0.0167) and 0.044 (0.8752 ± 0.0079 against 0.8311 ± 0.0168). At the larger capacity, then, the perceptron's advantage narrows to 0.003 under early stopping; under the published recipe, the perceptron receives 32,200 updates at that capacity against the KAN's 300. The direct test of the training budget in Section S21, however, shows that the perceptron trained for the KAN's number of updates still exceeds the KAN (0.883 against 0.860), so the narrowing reflects the early-stopping procedure rather

than the budget itself. We report both because the difference between them is the confound examined in this section.

Source of the discrepancy. The two attempts were run in separate processes that also differed in the number of compute threads they were given, and our first explanation was that floating-point reductions had been reordered and that early stopping had converted the resulting difference into a different stopping point. That explanation was incorrect on two counts, and the data that refute it were already available. The number of optimisation steps used is identical in the two attempts at each of the five seeds — 1000, 2000, 3200, 1100 and 2400 — so the two attempts stopped at the same point for every seed. Moreover, repeating the identical configuration at two thread counts returns the same value (0.9702 at both, seed 42), so the machine was not the cause either. What remained, once those were excluded, was the scoring. Section S8 quantifies the genuine run-to-run variation in this pipeline, which is two orders of magnitude smaller than the discrepancy this section had to explain.

This correction is reported because the incorrect explanation was the more favourable one. Attributing a 0.02 discrepancy to numerical noise would have left the second attempt's conclusion standing as one unlucky draw; tracing it to a metric that we ourselves had applied inconsistently meant that the comparison had to be redone.

Table S6. The parameter-matched comparison, in the three forms it was run, on tahini and on the two mango configurations ("accuracy" and "compact"). Block 1 applies the published training recipe, which gives the perceptron between twelve and one hundred and seven times more gradient updates than the KAN. Block 2 equalises the parameter count, the size of the hyperparameter search and the convergence criterion, and records the updates each architecture actually used; its tahini figures are reproduced exactly as that comparison produced them, that is, without the composition normalisation the published pipeline applies before scoring, which is why they disagree with block 3 and why the comparison was redone. Block 3 separates the three factors block 2 changed together — which configuration, how it was trained, and how it was scored — and is the one on which the conclusions rest. In it, "Config." is the configuration each model was given: "published" is the one selected in the benchmark of Section 3.2, which exists only for the KAN, and "inner-CV" the one the six-way search of this comparison selected. "Harness" is the training rule: "fixed steps" is the published recipe's fixed step count, and "early stopping" halts training on the basis of a grouped validation split.

Block	Dataset	Model	Config.	Harness	Parameters	Updates (median)	Grouped CV R^2	External-test R^2
1	mango, accuracy	KAN	—	—	8996	—	0.850 ± 0.009	0.862 ± 0.011
1	mango, accuracy	MLP (1 layer)	—	—	8989	—	0.863 ± 0.008	0.890 ± 0.007
1	mango, accuracy	MLP (2 layers)	—	—	8965	—	0.865 ± 0.005	0.887 ± 0.005
1	mango, compact	KAN	—	—	820	—	0.841 ± 0.002	0.829 ± 0.017
1	mango, compact	MLP (1 layer)	—	—	817	—	0.866 ± 0.003	0.874 ± 0.008
1	mango, compact	MLP (2 layers)	—	—	809	—	0.860 ± 0.002	0.857 ± 0.017
1	tahini	KAN	—	—	684	—	0.969 ± 0.007	0.983 ± 0.007
1	tahini	MLP (1 layer)	—	—	689	—	0.986 ± 0.003	0.992 ± 0.002
1	tahini	MLP (2 layers)	—	—	705	—	0.981 ± 0.002	0.984 ± 0.004
2	mango, accuracy	KAN	—	—	8996	1700	—	0.871 ± 0.015

Block	Dataset	Model	Config.	Harness	Parameters	Updates (median)	Grouped CV R^2	External-test R^2
2	mango, accuracy	MLP (matched)	—	—	8989	1300	—	0.876 ± 0.018
2	mango, compact	KAN	—	—	820	1400	—	0.831 ± 0.017
2	mango, compact	MLP (matched)	—	—	817	2500	—	0.875 ± 0.008
2	tahini	KAN	—	—	684	2000	—	0.949 ± 0.046
2	tahini	MLP (matched)	—	—	689	1600	—	0.991 ± 0.004
3	mango, accuracy	KAN	inner-CV	early stopping	8996	1600	—	0.873 ± 0.017
3	mango, accuracy	KAN	inner-CV	fixed steps	8996	300	—	0.873 ± 0.014
3	mango, accuracy	KAN	published	early stopping	8996	1100	—	0.859 ± 0.018
3	mango, accuracy	KAN	published	fixed steps	8996	300	—	0.862 ± 0.008
3	mango, accuracy	MLP (matched)	inner-CV	early stopping	8989	1300	—	0.876 ± 0.018
3	mango, accuracy	MLP (matched)	inner-CV	fixed steps	8989	32,200	—	0.897 ± 0.007
3	mango, compact	KAN	inner-CV	early stopping	820	1400	—	0.831 ± 0.017
3	mango, compact	KAN	inner-CV	fixed steps	820	300	—	0.812 ± 0.020
3	mango, compact	KAN	published	early stopping	820	1400	—	0.836 ± 0.015
3	mango, compact	KAN	published	fixed steps	820	300	—	0.827 ± 0.013
3	mango, compact	MLP (matched)	inner-CV	early stopping	817	2500	—	0.875 ± 0.008
3	mango, compact	MLP (matched)	inner-CV	fixed steps	817	32,200	—	0.873 ± 0.005
3	tahini	KAN	inner-CV	early stopping	684	2000	—	0.969 ± 0.034
3	tahini	KAN	inner-CV	fixed steps	684	300	—	0.974 ± 0.009
3	tahini	KAN	published	early stopping	684	1700	—	0.965 ± 0.041
3	tahini	KAN	published	fixed steps	684	300	—	0.986 ± 0.006
3	tahini	MLP (matched)	inner-CV	early stopping	689	1600	—	0.989 ± 0.003
3	tahini	MLP (matched)	inner-CV	fixed steps	689	3600	—	0.986 ± 0.003

S8. Run-to-Run Reproducibility

Section S7 addresses a discrepancy of 0.02 R^2 between two of our own runs; our first explanation — that floating-point reductions had been reordered under a different thread count — proved to be wrong. The effect was therefore measured directly. Holding fixed everything a user controls, we repeated identical runs three ways: inside one process, across two thread counts, and across random seeds, for both architectures and both training rules (Table S7). Spreads are reported as the range over the runs rather than as a standard deviation, because with two or three runs a range is what the data supports.

The perceptron is bit-reproducible; the KAN is not, although its run-to-run variation exceeds floating-point round-off under only one training rule. Repeated in one process with the seed fixed, and repeated across thread counts, the multilayer perceptron returns the identical number under both training rules — a range of exactly zero in every cell. The KAN under the published recipe returns the same number to 3×10^{-8}, which is attributable to floating-point round-off. The KAN under the early-stopping harness does not: its result varies by up to 7.3×10^{-5} between repeats in a single process and by about 1.6×10^{-3} when only the thread count changes. The cause is neither the architecture alone nor the machine alone, but the early-stopping rule applied to this architecture, which turns a sub-tolerance numerical difference into a different set of weights.

At the configuration at which our two runs actually disagreed, the machine accounts for almost none of the discrepancy. The comparison in Section S7 diverged at the configuration its own inner cross-validation selected, not at the published one, so we repeated the measurement there. Across thread counts and repeated runs the spread is at most 3.5×10^{-4}, and in one of the five seeds it is exactly zero; under the published recipe at the same configuration it is 8.3×10^{-8}. Compared with a discrepancy of 3.9×10^{-2} in the worst seed, the largest of these spreads is two orders of magnitude too small to explain it. The machine was not the cause; Section S7 reports the actual cause.

All of these effects are small compared with the effect of the seed. Changing the random seed alone moves the KAN by 1.2×10^{-2} under the published recipe and 1.6×10^{-2} under the convergence harness, and the perceptron by 1.8×10^{-3} to 3.8×10^{-3}. Within one architecture and one harness, the seed effect exceeds the machine-level effect by a factor of between about ten and more than 10^5 — and by an unbounded factor for the perceptron, whose machine-level spread is exactly zero. Only one machine-level cell comes close to any seed effect at all: the KAN's thread-count spread under the convergence harness, 1.6×10^{-3}, is of the same size as the perceptron's seed spread. The KAN's seed spread is three to nine times the perceptron's. This is the same instability Section 3.6 of the article reports as the architecture's real cost, seen from a second direction.

Three practical consequences follow, and we state them as recommendations because they cost nothing.

Report the distribution, not a run. Every figure in this section and in Sections 3.3–3.6 of the article is an average over seeds with its spread given, and every quoted equation names the seed that produced it. The exception is the benchmark of Tables 2 and 3, which is one grouped hold-out at one seed, reported with a cluster-bootstrap interval over resampled test groups and a fold-wise cross-validated spread rather than a seed spread; the "Random seed" row above gives the appropriate scale against which to read those two tables, and for the KAN it is 1.2×10^{-2}. A single number from a single seed is reproducible to eight decimal places and uncertain in its second.

Prefer the fixed-budget recipe when a result has to be reproduced. The published recipe — a fixed number of optimisation steps on the whole training partition — is reproducible here to round-off, across processes and thread counts. Early stopping is a better use of a training budget and a worse basis for a published number, and the choice between them should be made deliberately.

Do not attribute a discrepancy to numerical noise without measuring it. We initially adopted this explanation because it was the more comfortable one: it would have let the earlier conclusion stand and attributed the discrepancy to bad luck. The measurement bounded the machine's contribution two orders of magnitude below the effect and prompted us to look for the real cause, which we found. The general point holds beyond this paper — a run-to-run difference large enough to change a conclusion is almost never a floating-point effect.

https://doi.org/10.3390/foods15203639

Reproduction of the benchmark with the released code. The benchmark of Tables 2 and 3 of the article was rerun with the released code in a different software environment (Section 2.8 of the article). PLS, SVR, RF and the KAN reproduced exactly on tahini, and the MLP and the CNN to within 0.004 R^2. On mango, the inner cross-validation selected a different MLP configuration, (256, 128) hidden units on 24 PLS scores rather than the published (128, 64) on 16, because the two were nearly tied (mean inner R^2 0.866 against 0.864; script revision/r14_mango_mlp_tie.py). That configuration reached R^2 = 0.871 on the external test (revision/results/benchmark_reproduction/results_mango/Table_mango_hold-out.csv in the code repository; the published benchmark files are in results_mango/), above the KAN (0.863), so the order of the two leading mango models depends on a near-tie in model selection. The frozen leave-one-season-out analysis of Section S4 reproduced the original values to within 0.005 for four of the six models and to within 0.011 for the KAN. For SVR, the first withheld season gave 0.609 rather than 0.248; because none of the four SVR configurations in the search reproduced the original value, the SVR results in Section S4 are those of the rerun.

Table S7. Run-to-run reproducibility of this pipeline on tahini, with everything the user controls held fixed. Each cell is the range over the runs in that condition, and 0 means the runs returned bit-identical predictions. The first three rows use the published configuration and vary one thing at a time; the last row repeats the thread-count measurement at the configuration selected by inner cross-validation, which is the one at which two of our own runs disagreed and which is markedly more volatile across seeds.

Source of variation	Held fixed	KAN, published recipe	KAN, convergence harness	MLP, published recipe	MLP, convergence harness
Repeated runs in one process	seed, threads, split, configuration	3×10^{-8}	7.3×10^{-5}	0	0
Number of compute threads	seed, split, configuration	3×10^{-8}	1.6×10^{-3}	0	0
Random seed	threads, split, configuration	1.2×10^{-2}	1.6×10^{-2}	3.8×10^{-3}	1.8×10^{-3}
Number of compute threads	seed, split; inner-CV-selected configuration	8.3×10^{-8}	3.5×10^{-4}	not run	not run

S9. Stability of the Extracted Equation and of Its Retained Variables

An equation that changes on refitting is not a usable calibration, and one whose variables change is not a stable explanation. Section S8 measured the reproducibility of the training run; this section measures the reproducibility of the object that comes out of it. The whole chain — preprocessing, projection, network, pruning, conversion — was repeated on both datasets under four sources of variation and both extraction protocols, and two quantities were recorded each time: the accuracy of the equation, and the agreement between the sets of variables successive equations retain (Table S8). The results below show that an equation obtained by converting a B-spline network is not a reproducible calibration, whereas the exact route, in which the edge functions are already elementary, returned the same equation in every repeated run (Table S9).

On tahini the corrected protocol is more accurate on every axis, and in this execution of the sweep also more tightly clustered. Across ten random seeds the published protocol gives equations averaging R^2 0.859 ± 0.205, ranging from 0.324 to 0.969; the corrected protocol gives 0.979 ± 0.018, ranging from 0.943 to 0.994. Changing the cross-validation fold moves the published equation over 0.856–0.964 and the corrected one over 0.936–0.986; changing the preprocessing moves them over 0.858–0.962 and 0.983–0.990, and there the corrected protocol is the only one of the two that stays above its own spline network.

Unlike the accuracy claim, the claim of tighter clustering needs a caveat, and that caveat follows from this section's own main finding. The ten-seed rows here are a re-execution of the sweep summarised in Table 6 of the article: same ten seeds, same networks, same code. Table 6's execution gives the corrected protocol 0.884 ± 0.324 with a minimum of −0.038 — a *larger* spread than the published protocol's 0.205, not a smaller one — because at seed 777 the conversion failed in that execution but returned 0.958 in this one. The two executions differ only because the extraction is not reproducible, as measured directly below and in Section 3.3. Both figures are reported and neither is canonical; the accuracy improvement is the part that reproduces across both executions. The fixed-seed repeats row below is a second exception to any blanket clustering claim, and runs the other way.

On mango, the corrected protocol is not uniformly better, and we report this rather than generalising from tahini. The corrected protocol leads across cross-validation folds (0.850 ± 0.048 against 0.838 ± 0.058) and across repeated runs (0.861 ± 0.002 against 0.842 ± 0.000), but across seeds the two are level and the corrected one is the *more* variable (0.845 ± 0.018 against 0.844 ± 0.010), and across preprocessing choices it is slightly worse (0.720 ± 0.087 against 0.728 ± 0.103) at a markedly lower fidelity. The correction is therefore worth making — it never costs much and on tahini it transforms the distribution — but it offers no guarantee, and a practitioner adopting it for a new matrix should verify its benefit there rather than assume it.

One source of variation has the opposite effect on both datasets, and it is the one least under the user's control. Repeated *identical* runs — same seed, same data, same configuration, differing only in what floating-point arithmetic does between them — leave the published protocol unchanged to four decimal places (0.954 to 0.954 on tahini; 0.842 to 0.842 on mango) while the corrected protocol varies from 0.989 to 0.990 and from 0.860 to 0.863. The correction that fixes the accuracy introduces non-determinism of its own, and the comparison localises its source: the published protocol, which has no refit, reproduces to about 5×10^{-5} at every seed we have measured, so the variation enters at or after the post-symbolic refit. We do not claim to have identified the mechanism beyond that.

That measurement is made at the published seed, and it flatters the corrected protocol. The five repeats above are all at seed 42, where the corrected protocol's run-to-run range is 1.7×10^{-3}. Repeating the same measurement at two other seeds gives a very different picture. The trained spline network reproduces to within 2×10^{-7} — external R^2 0.959643 at seed 777 and 0.982985 at seed 13, to six decimal places, in every one of the runs below — so the variation reported below arises entirely in the conversion, and the contrast is seven orders of magnitude: 2×10^{-7} in the network against 1.71 in the equation drawn from it. From that one network at seed 777, six runs of the same nominal protocol produced equations scoring −0.755, −0.038, 0.950, 0.951, 0.953 and 0.958; at seed 13, 0.845, 0.940, 0.943, 0.984, 0.985 and 0.985. The run-to-run range is therefore 1.7×10^{-3} at one seed, 0.14 at another and 1.71 at a third, and nothing available in advance says which kind of seed one is looking at. By contrast the published protocol — the one without the refit — reproduces to about 5×10^{-5} at every seed we have measured, so the irreproducibility belongs to the step that recovers the accuracy.

Where the variation does not arise, and what is free of it (Table S9). Both routes were repeated three times at each of two compute-thread settings and at each of the two seeds, with the random state of both the tensor library and the numerical library set identically before every run, to test the obvious explanation — that a threaded floating-point reduction reorders a comparison and flips a discrete choice inside the conversion. The explanation fails this test. These runs are separate from the six quoted above, which is why their values differ. At a *single* thread the converted equation ranges over 0.795 to 0.959 at seed 777 and over 0.815 to 0.985 at seed 13, ranges of 0.164 and 0.170, against 0.136 and 0.035 at

https://doi.org/10.3390/foods15203639

four threads — if anything the single-threaded runs are the more variable. Whatever the mechanism is, it is not the compute environment, and we do not claim to have identified it further.

The control is the informative half of that experiment. The elementary-basis route returns the same equation in every one of its twelve runs — 0.983753 at seed 777 and 0.985279 at seed 13, at fidelities of 0.999996 and 0.999995, with a range of exactly zero at both thread counts. It has no conversion step in which any of this can happen. It is also, at both seeds, the more accurate of the two routes.

Two consequences follow, and the second is the one we would want a reader to take away. A failed extraction is a property of a run, not of a seed. Section 3.3 of the article reports that under the corrected protocol one of ten seeds returned an unusable equation from a perfectly good network; that is what that run found, and rerunning the identical extraction from the identical network returns a usable equation four times in six. Quoting a seed alongside an equation, as we do throughout, is therefore necessary but not sufficient. Fidelity does, however, detect these particular failures. In the two unusable runs at seed 777 the fidelity of the printed equation to its own network was −0.956 and −0.246 — the formula is *anti-correlated* with the model it claims to represent — against 0.947 to 0.958 in the four usable ones, and 0.845 in the weakest run at seed 13. No held-out data are needed to detect them. How far this generalises is measured below; the answer is only half as reassuring as these two runs suggest.

The variables an equation retains are more stable than its score and do not depend on the protocol at all. On tahini the variables successive equations retain agree with a mean pairwise top-3 overlap of 0.81 across seeds, 0.87 across folds and 0.78 across preprocessing choices, with Spearman correlations of 0.81, 0.68 and 0.84; across repeated runs at one seed the agreement is exact. On mango the top-3 overlap is exactly 1.00 across seeds, folds and repeats, with Spearman 0.78, 0.76 and 1.00 — the equation there names the same leading variables every time. Those figures are *identical* for the two protocols, and necessarily so: the importance probe reads the trained spline network, which is the same object under both, and only the conversion differs. Correcting the conversion therefore improves the equation's score without changing what it identifies as important. This is worth stating because it means that the chemical reading of Section S10 does not depend on the protocol choice of Section 3.3 of the article.

The exception locates the real weakness. On mango, varying the preprocessing degrades both quantities at once: the top-3 overlap falls to 0.33, the lowest figure anywhere in this table, at the same time as the equation's accuracy falls to 0.72–0.73 and its fidelity to 0.73–0.85. Changing the scatter correction and derivative changes the latent variables themselves, so an equation written in them is not merely refitted but rewritten in a different basis. That is a limitation of writing a calibration in latent variables rather than of the extraction, and it is one more reason to prefer the named-band form of Section 3.4, whose variables are fixed absorbances that no preprocessing choice can redefine.

How well does checking fidelity actually work? Recommending this check is worthwhile only if it discriminates, so we measured how well it does. Pooling every extraction performed in this project — 288 of them, across the protocol comparison, this stability sweep, the probe and determinism tests and the distribution-shift folds — gives its operating characteristic as a filter (Table S10). The answer is sharply two-sided, and we report both halves.

Fidelity reliably detected catastrophic failures. Four of the 288 equations scored below R^2 0.5. Their fidelities were −0.956, −0.246, 0.439 and 0.439, and the lowest fidelity among the other 284 was 0.479. Discarding every equation with a fidelity below 0.5 therefore removes all four at the cost of one sound equation. A practitioner who extracts several times and discards any equation whose fidelity falls below one half will not print a broken formula.

Fidelity did not reliably detect moderate shortfalls. Twenty-six equations fell more than 0.05 R^2 below the network they were extracted from, and no threshold separates those cleanly: at 0.9 the filter still keeps nine of them while discarding twelve sound ones, and the highest fidelity among the twenty-six is 0.975. An equation can be a tenth of a unit of R^2 worse than its own model while still reproducing 97.5% of the variance in that model's predictions — the two quantities measure different things, and agreement in the middle of the distribution does not bound disagreement in the tails. Only a threshold of 0.98 removes all twenty-six, and it discards 137 of the 262 sound equations with them.

The check should therefore be made, but interpreted in one direction only: a low fidelity is strong evidence that an equation is unusable, and a high one is weak evidence that it is faithful. That is a weaker claim than we expected to be able to make, and it is one more reason to prefer a route in which the question does not arise.

The practical rule follows from the numbers rather than from caution. A single extraction is not a result. The seed alone moves the published tahini equation across two thirds of the available range and the corrected one across 0.05; the fold and the preprocessing each add further variation. Every equation quoted in this paper therefore names its seed and is reported beside the distribution it was drawn from, and a practitioner should extract several times, compare the retained variables, and publish the spread rather than the best draw.

Table S8. Stability of the extracted equation and of the variables it retains, under four sources of variation and both extraction protocols, on both datasets. "Top-3 overlap" and "Spearman ρ" are mean pairwise agreements between the variable importances of the equations obtained in that condition; they are identical for the two protocols because the importance probe reads the trained network, which the conversion does not alter. The spline-network column gives the accuracy of the network from which each equation was extracted and is likewise shared. The "10 random seeds" rows are a separate execution of the sweep summarised in Table 6 of the article, at the same seeds and from the same networks; the two executions differ because the extraction is not reproducible, and both are reported rather than one replacing the other.

Dataset	Protocol	Source of variation	Runs	Spline network R^2	Equation R^2	Equation R^2 range	Median retained variables	Mean pairwise top-3 overlap	Mean pairwise Spearman ρ
tahini	P0 published	10 random seeds	10	0.955 ± 0.065	0.859 ± 0.205	0.324–0.969	7	0.81	0.81
tahini	P0 published	5 repeats, fixed seed	5	0.983 ± 0.000	0.954 ± 0.000	0.954–0.954	7	1.00	1.00
tahini	P0 published	5 grouped CV folds	5	0.945 ± 0.026	0.923 ± 0.044	0.856–0.964	7	0.87	0.68
tahini	P0 published	3 alternative preprocessings	3	0.978 ± 0.011	0.916 ± 0.053	0.858–0.962	5	0.78	0.84
tahini	P3 corrected	10 random seeds	10	0.955 ± 0.065	0.979 ± 0.018	0.943–0.994	7	0.81	0.81
tahini	P3 corrected	5 repeats, fixed seed	5	0.983 ± 0.000	0.990 ± 0.001	0.989–0.990	7	1.00	1.00
tahini	P3 corrected	5 grouped CV folds	5	0.945 ± 0.026	0.973 ± 0.021	0.936–0.986	7	0.87	0.68
tahini	P3 corrected	3 alternative preprocessings	3	0.978 ± 0.011	0.987 ± 0.003	0.983–0.990	5	0.78	0.84
mango	P0 published	10 random seeds	10	0.825 ± 0.017	0.844 ± 0.010	0.823–0.857	11	1.00	0.78

Dataset	Protocol	Source of variation	Runs	Spline network R^2	Equation R^2	Equation R^2 range	Median retained variables	Mean pairwise top-3 overlap	Mean pairwise Spearman ϱ
mango	P0 published	5 repeats, fixed seed	5	0.818 ± 0.000	0.842 ± 0.000	0.842–0.842	11	1.00	1.00
mango	P0 published	5 grouped CV folds	5	0.847 ± 0.050	0.838 ± 0.058	0.744–0.885	12	1.00	0.76
mango	P0 published	3 alternative preprocessings	3	0.797 ± 0.021	0.728 ± 0.103	0.610–0.800	12	0.33	0.73
mango	P3 corrected	10 random seeds	10	0.825 ± 0.017	0.845 ± 0.018	0.798–0.861	11	1.00	0.78
mango	P3 corrected	5 repeats, fixed seed	5	0.818 ± 0.000	0.861 ± 0.002	0.860–0.863	11	1.00	1.00
mango	P3 corrected	5 grouped CV folds	5	0.847 ± 0.050	0.850 ± 0.048	0.766–0.885	12	1.00	0.76
mango	P3 corrected	3 alternative preprocessings	3	0.797 ± 0.021	0.720 ± 0.087	0.620–0.776	12	0.33	0.73

Table S9. Is the extraction reproducible, and does the exact route share the problem? Both routes were repeated three times at each of two compute-thread settings on the tahini training partition, with the random state of the tensor library and of the numerical library set identically before every run. "Network R^2" is the accuracy of the network from which each equation was extracted and is identical across every run in its block. A range of zero means the runs returned the same equation to six decimal places.

Route	Seed	Compute threads	Runs	Network R^2	Equation R^2, lowest	Equation R^2, highest	Range	Fidelity, lowest	Fidelity, highest
Corrected conversion	13	1	3	0.982985	0.814507	0.984631	0.170124	0.822177	0.980706
Corrected conversion	13	4	3	0.982985	0.925278	0.960230	0.034952	0.934358	0.966267
Elementary basis	13	1	3	0.985279	0.985279	0.985279	0.000000	0.999995	0.999995
Elementary basis	13	4	3	0.985279	0.985279	0.985279	0.000000	0.999995	0.999995
Corrected conversion	777	1	3	0.959643	0.795457	0.958967	0.163510	0.720299	0.951377
Corrected conversion	777	4	3	0.959643	0.821263	0.957093	0.135831	0.800407	0.954286
Elementary basis	777	1	3	0.983753	0.983753	0.983753	0.000000	0.999996	0.999996
Elementary basis	777	4	3	0.983753	0.983753	0.983753	0.000000	0.999996	0.999996

Table S10. How well fidelity works as a filter, pooled over all 288 extractions this study performed. "Failures kept" is the number of failed equations a filter at that threshold would let through; "sound equations discarded" is what it would throw away needlessly; "worst shortfall still kept" is the largest gap between an equation and its own network among the equations kept. The two blocks differ only in what counts as a failure, and they give opposite verdicts on the same filter.

Failure defined as	Fidelity threshold	Equations kept	Failures kept	Failures caught	Sound equations discarded	Worst shortfall still kept
catastrophic (equation $R^2 < 0.5$)	0.00	286	2	2	0	0.452

Failure defined as	Fidelity threshold	Equations kept	Failures kept	Failures caught	Sound equations discarded	Worst shortfall still kept
catastrophic (equation R^2 < 0.5)	0.50	283	0	4	1	0.249
catastrophic (equation R^2 < 0.5)	0.70	278	0	4	6	0.249
catastrophic (equation R^2 < 0.5)	0.80	270	0	4	14	0.248
catastrophic (equation R^2 < 0.5)	0.90	259	0	4	25	0.105
catastrophic (equation R^2 < 0.5)	0.95	235	0	4	49	0.105
catastrophic (equation R^2 < 0.5)	0.98	125	0	4	159	0.018
catastrophic (equation R^2 < 0.5)	0.99	93	0	4	191	0.003
material (falls > 0.05 R^2 below its network)	0.00	286	24	2	0	0.452
material (falls > 0.05 R^2 below its network)	0.50	283	21	5	0	0.249
material (falls > 0.05 R^2 below its network)	0.70	278	20	6	4	0.249
material (falls > 0.05 R^2 below its network)	0.80	270	17	9	9	0.248
material (falls > 0.05 R^2 below its network)	0.90	259	9	17	12	0.105
material (falls > 0.05 R^2 below its network)	0.95	235	3	23	30	0.105
material (falls > 0.05 R^2 below its network)	0.98	125	0	26	137	0.018
material (falls > 0.05 R^2 below its network)	0.99	93	0	26	169	0.003

S10. The Learned Response Curves, the Band Assignments, and the Chemical Reading

The article gives the calibration as an equation. This section gives the two things that make the equation readable rather than merely printable: the shape of each learned univariate function, and the spectroscopic meaning of the variables on which those functions act.

The learned functions, against the straight line a linear model would have fitted. Each edge function is drawn on the same axes as the linear effect of the same variable, so that

any departure from the dashed line marks where — and how much — non-linearity is being used (Figures S5 and S6). The annotation "dev" on each panel is the largest absolute difference between the two curves over the input range, in units of the standardised target.

On tahini (Figure S5) the departures are concentrated in the two leading latent variables, and they have a recognisable form: x_1 tracks the linear effect closely up to about the upper quartile of its range and then turns over rather than continuing to rise (dev 1.85), and x_2 flattens at both ends instead of descending throughout (dev 1.66). Both are single bends at the edges of the range, not oscillations — the signature of a response that is linear where the calibration has most of its data and bends where it has least. The remaining six variables deviate by 0.04–1.04 on a much smaller vertical scale. This is the picture the article's near-linearity claim predicts, seen from the model's own side: a non-linear model given a near-linear problem chooses a nearly straight function and spends its flexibility at the edges of the range.

On mango (Figure S6) the curves differ from those of tahini in shape rather than in the size of the departures, consistent with, but not demonstrating, the water-band account. Every mango variable departs from its linear effect by less than tahini's leading two do, and the departures are spread across the twelve rather than concentrated in the first: the largest are x_1 (dev 1.00), x_4 (0.78) and x_8 (0.58), and the smallest x_{12} (0.05). Tahini's two leading departures are single bends at the edges of the range; several of mango's are not — x_7 (dev 0.35) reverses sign in the middle of its range, x_9 (0.39) rises and falls again, and x_{10} (0.31) has a pronounced minimum near the lower end. Dry matter is read indirectly through the water bands, on a scattering background that may vary with fruit size, skin and temperature, so a monotone response is not necessarily expected. Whether these shapes reflect the recorded covariates was not tested (Section S18 examines prediction residuals only), and fruit size and skin properties were not recorded, so their origin is not established. It is worth being clear about what this does and does not show: these shapes are what the model *learned*, and Section 3.6 of the article reports that learning them confers no accuracy advantage over a perceptron of the same size. What they offer instead is that they can be inspected.

What the variables mean. The loading vectors of the leading three latent variables are projected back onto the spectral axis in Figure S7, with tabulated band positions marked. In the mid-infrared (Figure S7a) the three loadings carry little structure between about 2800 and 1800 cm^{-1} — the region of the spectrum where these pastes differ least — and all three change character abruptly at the ester carbonyl near 1745 cm^{-1}, remaining strongly structured through the CH_2/CH_3 bending and C–O stretching envelopes below it. This is the same region in which Figure 2b of the article shows the two adulterants departing from tahini in opposite directions; the latent variables underlying the calibration therefore draw on the part of the spectrum that carries the discriminating information rather than on the dominant but shared C–H and C=O intensities. In the near-infrared (Figure S7b) the loadings are smooth, a consequence of the first-derivative Savitzky–Golay preprocessing, and several of their extrema lie close to tabulated overtone positions — x_2 peaks at the O–H/C–H third overtone near 770 nm and x_3 near the C–H third overtone at about 915 nm — while others, such as x_1's maximum near 880 nm, fall between tabulated bands. The correspondence is therefore partial.

The named bands and their assignments. Table S11 lists every channel the band selection of Section 3.4 of the article retained — the sixteen tahini channels its headline comparison is fitted on, and the eight mango ones — with the assignment the cited sources support and its provenance. The equation prunes further within that set, retaining a median of six of the sixteen over five seeds — four to nine, with only three bands, 1487, 1286 and 1242 cm^{-1}, common to every seed (Section 3.4). The eight that Equation (3) is fitted on

and keeps in full are marked in the last column. In summary, the assignments are only partly available, and less so for the equation than for the set it was selected from: none of the eight channels Equation (3) retains is a band the cited sources tabulate — four of them (1286, 1242, 1180 and 989 cm^{-1}) fall in described regions and four (1502, 1487, 1447 and 1140 cm^{-1}) are unassigned. This is a consequence of the selection: the channels were ranked by their contribution to a linear projection, not by whether they had been assigned, and the two tabulated bands among the sixteen are pruned away. Of the sixteen tahini channels, two fall inside a band tabulated in the cited sources, nine fall inside a region those sources describe but do not tabulate as a discrete band, and five fall outside every tabulated range and are reported as unassigned rather than given an assignment by proximity. Of the eight mango bands, three are tabulated — the O–H second overtone at 981 nm and two C–H third-overtone positions at 924 and 903 nm — and five are unassigned. We prefer an explicit "unassigned" to a plausible label, because a band assignment chosen for its nearness to a selected channel is a post-hoc rationalisation, and an equation whose variables are half-explained is a more useful object than one whose variables are all explained unreliably.

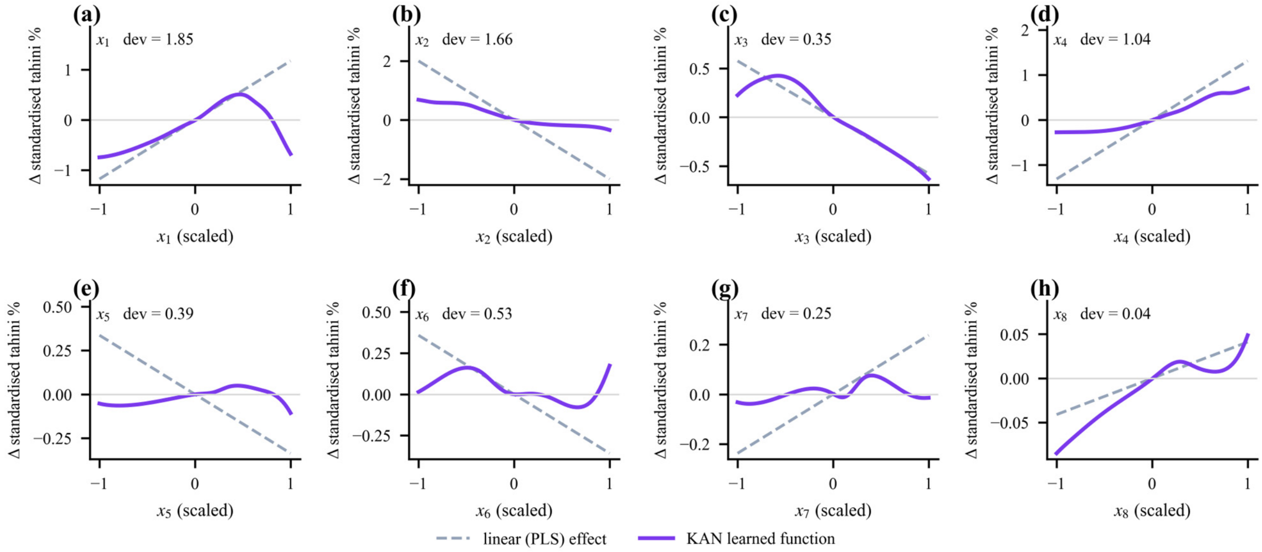

Figure S5. Tahini: the function the network learned on each of the eight latent variables (solid), against the linear effect of the same variable (dashed). "dev" is the largest absolute difference between the two over the input range, in units of the standardised target. Curves are centred at the midpoint of the range so that only the shape is compared.

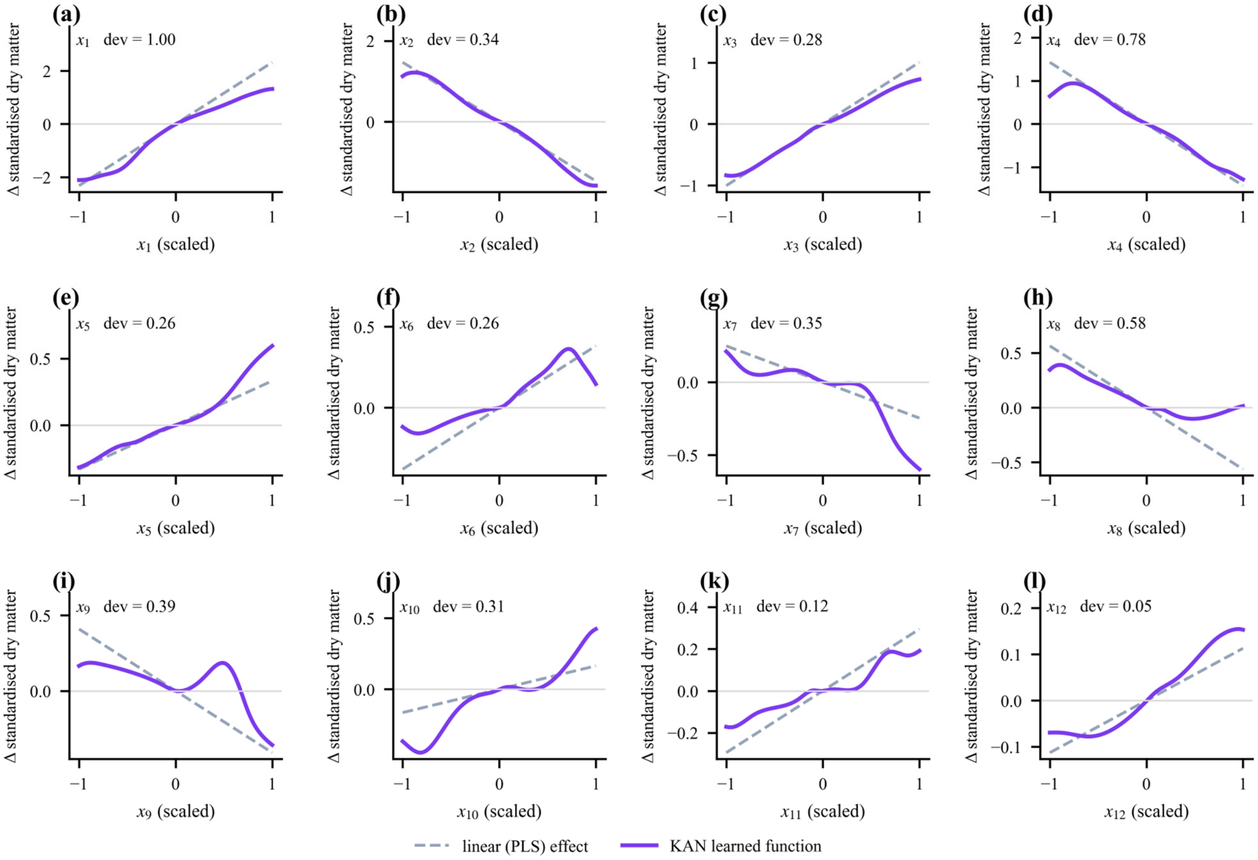

Figure S6. Mango: the function the network learned on each of the twelve latent variables of the equation-bearing configuration, against the linear effect of the same variable. Conventions as in Figure S5.

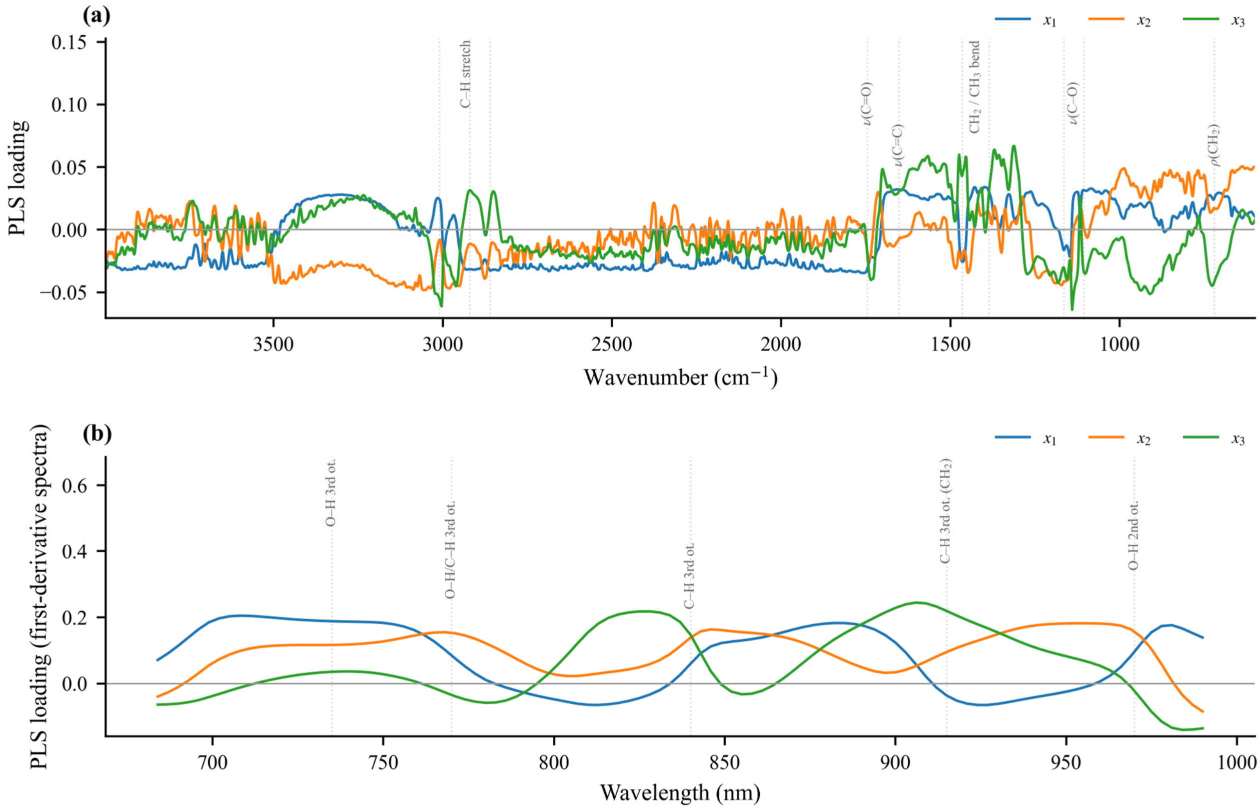

Figure S7. What the equation's variables correspond to spectroscopically. (a) Loading vectors of the leading three tahini latent variables on the mid-infrared axis; (b) the same for mango on the near-infrared axis, computed on first-derivative spectra. Dotted lines mark tabulated band positions [48–50].

Table S11. Channels retained by the band selection of Section 3.4 — sixteen on tahini, eight on mango — and their vibrational assignments. These are the candidate bands available to the equation, which prunes within them; the last column marks the eight tahini channels on which Equation (3) is fitted and which it retains in full. Where a channel falls outside every range tabulated in the cited sources it is reported as unassigned rather than given an assignment by proximity. Positions are rounded to the nearest wavenumber for display; the assignment is made against the unrounded channel.

Dataset	Order	Position	Unit	Assignment	Associated with	Source of the assignment	In Equation (3)
tahini	1	3022	cm^{-1}	unassigned	—	no tabulated assignment	—
tahini	2	3005	cm^{-1}	=C–H stretch (cis C=C)	Unsaturated fatty acids	tabulated band	—
tahini	3	2962	cm^{-1}	C–H stretch region (CH_3 asymmetric)	Lipid acyl chains	region, not a tabulated band	—
tahini	4	1502	cm^{-1}	unassigned	—	no tabulated assignment	yes
tahini	5	1487	cm^{-1}	unassigned	—	no tabulated assignment	yes
tahini	6	1472	cm^{-1}	CH_2 bending (scissor)	Lipids	tabulated band	—
tahini	7	1447	cm^{-1}	unassigned	—	no tabulated assignment	yes
tahini	8	1302	cm^{-1}	C–O stretch / CH_2 wagging region	Esters, acyl chains	region, not a tabulated band	—
tahini	9	1286	cm^{-1}	C–O stretch / CH_2 wagging region	Esters, acyl chains	region, not a tabulated band	yes
tahini	10	1242	cm^{-1}	C–O stretch / CH_2 wagging region	Esters, acyl chains	region, not a tabulated band	yes
tahini	11	1227	cm^{-1}	C–O stretch / CH_2 wagging region	Esters, acyl chains	region, not a tabulated band	—
tahini	12	1196	cm^{-1}	C–O stretch / CH_2 wagging region	Esters, acyl chains	region, not a tabulated band	—
tahini	13	1180	cm^{-1}	C–O stretch / CH_2 wagging region	Esters, acyl chains	region, not a tabulated band	yes
tahini	14	1140	cm^{-1}	unassigned	—	no tabulated assignment	yes
tahini	15	989	cm^{-1}	=C–H out-of-plane bend region	Unsaturation (trans)	region, not a tabulated band	yes
tahini	16	974	cm^{-1}	=C–H out-of-plane bend region	Unsaturation (trans)	region, not a tabulated band	—

Dataset	Order	Position	Unit	Assignment	Associated with	Source of the assignment	In Equation (3)
mango	1	990	nm	unassigned	—	no tabulated assignment	—
mango	2	981	nm	O–H 2nd overtone	Water (complement of dry matter)	tabulated band	—
mango	3	942	nm	unassigned	—	no tabulated assignment	—
mango	4	933	nm	unassigned	—	no tabulated assignment	—
mango	5	924	nm	C–H 3rd overtone (CH_2/CH_3)	Carbohydrate	tabulated band	—
mango	6	903	nm	C–H 3rd overtone (CH_2/CH_3)	Carbohydrate	tabulated band	—
mango	7	894	nm	unassigned	—	no tabulated assignment	—
mango	8	885	nm	unassigned	—	no tabulated assignment	—

S11. Intrinsic Importance Compared with Post-Hoc Attribution

The claim that an intrinsically interpretable model reports directly what a black box yields only through an external explainer is a comparison, and this section tests it as one. A first, smaller version of this analysis had three weaknesses. Its SHAP budget was fixed, so a modest rank correlation could not be distinguished from a sampling artefact; it computed SHAP only for the KAN, so the models were not compared on an equal basis; and it measured agreement on PLS latent variables, which is agreement about latent variables and not about chemistry. All three are addressed below.

The agreement is converged, not a sampling artefact (Table S12). SHAP was recomputed over a ladder of three budgets — background sets of 50, 100 and 200 spectra, 150, 300 and 600 evaluation rows, and 100, 200 and 400 coalition samples — for support-vector regression, the random forest, the perceptron and the KAN. On tahini, the rank correlation between permutation importance and SHAP is flat across that ladder for every model: 0.676, 0.683 and 0.690 for support-vector regression, and unchanged at 0.880, 0.952 and 0.905 for the random forest, the perceptron and the KAN. The correlation between the network’s own intrinsic importance and SHAP is likewise 0.810 at all three budgets, and against permutation importance 0.857 at all three. The largest budget, meanwhile, costs between two and a half and nine times as much computation as the smallest on tahini, and four to eight times as much on mango. These tahini numbers are therefore not undersampled.

On mango the same ladder is not flat, and we do not claim convergence there. Over the twenty-four latent variables of the mango accuracy configuration — sixteen for the perceptron — the permutation-against-SHAP correlation moves from 0.879 to 0.806 for the perceptron across the three budgets and from 0.738 to 0.762 for the KAN, and the intrinsic-against-SHAP correlation from 0.768 to 0.817; only support-vector regression is stable (0.857, 0.860, 0.855). No model changes character across the ladder — every one stays between 0.74 and 0.88 — but the values move, and a reader should treat them as accurate to about a tenth rather than to three decimals.

One qualification applies to all of these values: a Spearman correlation over eight variables is a coarse statistic that moves in steps of about 0.024, so “unchanged across the ladder” means the induced ranking did not change, and a value quoted to three decimals

https://doi.org/10.3390/foods15203639

should be read as one rank ordering rather than as a precise quantity. An independent run of the same tahini comparison at the middle budget returned 0.762 rather than 0.810 for the intrinsic-against-SHAP correlation, which is two of those steps.

Where the accounts differ is in the variables all of them consider unimportant (Figure S8). The three accounts agree on which variables matter and disagree on how much. On tahini all three place x_1 in the leading pair and the same four variables, x_1 to x_4, in the leading four; they do not agree on the order within that group, the intrinsic account ranking x_3 (0.212) marginally above x_2 (0.206) where permutation and SHAP both put x_2 first. The intrinsic account is also flatter across its leading four (0.252, 0.212, 0.206, 0.197) than either explainer, both of which fall away sharply by rank four (0.080 and 0.105). The sharpest disagreements are at the bottom. Permutation importance assigns x_6 and x_7 essentially zero (0.0004 and 0.000) where the network assigns them 0.051 and 0.023 and SHAP 0.025 and 0.027. Permutation works by destroying one variable at a time, so a variable whose contribution is duplicated by a correlated neighbour registers as worthless even when the fitted model does use it; the intrinsic account and SHAP, which do not work by deletion, agree that these carry a small but non-zero effect. None of this establishes which account is correct — there is no ground truth against which any of the three could be scored, and we make no claim that the intrinsic account is more correct. The intrinsic account is, however, free, exact for the model, and available without choosing a background set, a coalition count or a perturbation scheme, each of which is a decision that an explainer imposes on the user.

On the wavenumber axis, where the chemical information resides, the models agree less than the latent comparison suggests (Table S13). Attributions were mapped onto the spectrum in two ways — by propagating latent attributions through the PLS loadings, and by permuting contiguous spectral windows as blocks, which is model-agnostic and so places PLS and the convolutional network, neither of which sees a latent score, on the same axis as the rest. Over the twenty-one pairwise comparisons on tahini the mean Spearman correlation is 0.612 and the median overlap of the five highest-ranked windows is 0.8. Between fitted models it ranges from 0.385 (support-vector regression against the convolutional network) to 0.793 (perceptron against the KAN), and the KAN's *projected intrinsic* importance agrees with the block-permutation account of the KAN itself at 0.757 — better than it agrees with any other model, and better than several model-to-model pairs, but not identical. The two are measuring different things: one reads the trained edge functions, the other deletes windows of the spectrum and measures the resulting change in the prediction. That they agree at 0.76 rather than at 1.00 indicates how far an intrinsic account and a perturbation account of the same model can be expected to coincide.

On mango the agreement is much weaker, which is the most important negative result in this section. The same twenty-one comparisons on the mango wavelength axis give a mean Spearman correlation of 0.224 against tahini's 0.612, and a median top-five overlap of 0.2 against 0.8; seven of the twenty-one pairs share no window at all in their top five. Three correlations are *negative* — PLS against the random forest (−0.135), PLS against the convolutional network (−0.041), and PLS against the KAN's projected intrinsic importance (−0.086) — which is to say that on this matrix two accepted calibrations of the same property can rank the spectrum in opposite orders. The KAN's intrinsic account and the block-permutation account *of the same fitted KAN* agree at 0.048, against 0.757 on tahini. The best-agreeing mango pair, PLS against the block-permutation attribution of the KAN at 0.741, is reached or exceeded by only two of the twenty-one tahini pairs, and no other mango pair exceeds 0.71.

We do not read this as a defect of any one method. Dry matter is read indirectly, through water bands superimposed on a scattering background, over 103 strongly collin-

ear channels; where many different weightings of the spectrum give nearly the same prediction, attribution is not identified, and methods that differ in how they perturb the input will differ in what they credit. What it does mean is that the readability this paper offers is a property of the *equation*, which is exact for the model that produced it, and not a claim that the model's variables identify the chemistry. On tahini the two coincide well enough to be worth reading together; on mango they do not, and Section S10's mango band assignments should be read with that in mind.

One further negative result is reported here because it qualifies Section 3.4 of the article. The two windows that between them lead every model's account are centred near 1490 and 1235 cm^{-1}. Four of the six fitted models put 1490 first (PLS 0.429, the perceptron 0.243, support-vector regression 0.214, the random forest 0.181), as does the KAN's projected intrinsic importance; the KAN's own block-permutation account and the convolutional network put 1235 first instead (0.191 and 0.118, against 0.157 and 0.011 at 1490). Neither falls inside a band tabulated in the sources we cite; the 1490 window is reported as unassigned in Section S10, and the ~1235 window only as lying in the C–O stretching and CH_2 wagging envelope, a region those sources describe but do not tabulate as a discrete band. The most informative part of this spectrum, on every model's account and by two independent methods, is a region for which we can offer no vibrational assignment. No assignment is proposed for it; a candidate absorber near 1490 cm^{-1} is discussed, as a hypothesis only, in Section S14.

What this section supports. The interpretability claimed in the article is that the calibration is a closed-form equation that can be inspected and evaluated and, on the exact route, is the fitted model itself. The results of this section show that this interpretability does not extend to identifying the chemistry on mango, where attribution is not identified, and that on tahini the attribution methods agree well enough for the selected channels to be read together with the composition-dependence tests of Section S14.

Table S12. Agreement between attribution methods on the PLS latent variables, over a ladder of three SHAP budgets. "ρ permutation vs SHAP" is available for every model; the last two columns correlate the KAN's own intrinsic per-variable importance against each model's SHAP and permutation importances, and are not reported (—) where the model's latent dimension differs from the KAN's. The ladder tests whether the correlations change with the budget.

Dataset	Model	Axis	SHAP background	SHAP evaluation rows	SHAP samples	ρ permutation vs SHAP	ρ KAN intrinsic vs SHAP	ρ KAN intrinsic vs permutation
tahini	SVR	PLS latent variables	50	150	100	0.676	—	—
tahini	SVR	PLS latent variables	100	300	200	0.683	—	—
tahini	SVR	PLS latent variables	200	600	400	0.690	—	—
tahini	RF	PLS latent variables	50	150	100	0.880	—	—
tahini	RF	PLS latent variables	100	300	200	0.880	—	—
tahini	RF	PLS latent variables	200	600	400	0.880	—	—
tahini	MLP	PLS latent variables	50	150	100	0.952	0.857	0.857
tahini	MLP	PLS latent variables	100	300	200	0.952	0.857	0.857
tahini	MLP	PLS latent variables	200	600	400	0.952	0.857	0.857
tahini	KAN	PLS latent variables	50	150	100	0.905	0.810	0.857

Dataset	Model	Axis	SHAP background	SHAP evaluation rows	SHAP samples	ρ permutation vs SHAP	ρ KAN intrinsic vs SHAP	ρ KAN intrinsic vs permutation
tahini	KAN	PLS latent variables	100	300	200	0.905	0.810	0.857
tahini	KAN	PLS latent variables	200	600	400	0.905	0.810	0.857
mango	SVR	PLS latent variables	50	150	100	0.857	0.772	0.656
mango	SVR	PLS latent variables	100	300	200	0.860	0.761	0.656
mango	SVR	PLS latent variables	200	600	400	0.855	0.738	0.656
mango	RF	PLS latent variables	50	150	100	0.772	0.600	0.797
mango	RF	PLS latent variables	100	300	200	0.777	0.709	0.797
mango	RF	PLS latent variables	200	600	400	0.796	0.717	0.797
mango	MLP	PLS latent variables	50	150	100	0.879	—	—
mango	MLP	PLS latent variables	100	300	200	0.865	—	—
mango	MLP	PLS latent variables	200	600	400	0.806	—	—
mango	KAN	PLS latent variables	50	150	100	0.738	0.768	0.759
mango	KAN	PLS latent variables	100	300	200	0.788	0.836	0.759
mango	KAN	PLS latent variables	200	600	400	0.762	0.817	0.759

Table S13. Pairwise agreement between the models' attributions on the wavenumber axis, obtained by permuting contiguous spectral windows as blocks so that models which never see a latent score are placed on the same axis. "KAN intrinsic (projected)" is the network's own per-variable importance mapped onto wavenumbers through the PLS loadings, so its comparison with the KAN's own block-permutation row measures how far an intrinsic account and a perturbation account of one model coincide.

Dataset	Comparison	Spearman ρ	Top-5 window overlap
tahini	PLS vs SVR	0.484	0.80
tahini	PLS vs RF	0.577	1.00
tahini	PLS vs MLP	0.719	1.00
tahini	PLS vs CNN	0.675	0.80
tahini	PLS vs KAN	0.723	1.00
tahini	PLS vs KAN intrinsic (projected)	0.410	0.80
tahini	SVR vs RF	0.704	0.80
tahini	SVR vs MLP	0.688	0.80
tahini	SVR vs CNN	0.385	0.60
tahini	SVR vs KAN	0.548	0.80
tahini	SVR vs KAN intrinsic (projected)	0.466	0.80
tahini	RF vs MLP	0.702	1.00
tahini	RF vs CNN	0.460	0.80
tahini	RF vs KAN	0.679	1.00
tahini	RF vs KAN intrinsic (projected)	0.713	0.80
tahini	MLP vs CNN	0.626	0.80
tahini	MLP vs KAN	0.793	1.00

https://doi.org/10.3390/foods15203639

Dataset	Comparison	Spearman ρ	Top-5 window overlap
tahini	MLP vs KAN intrinsic (projected)	0.611	0.80
tahini	CNN vs KAN	0.638	0.80
tahini	CNN vs KAN intrinsic (projected)	0.485	0.60
tahini	KAN vs KAN intrinsic (projected)	0.757	0.80
mango	PLS vs SVR	0.701	0.40
mango	PLS vs RF	−0.135	0.00
mango	PLS vs MLP	0.449	0.00
mango	PLS vs CNN	−0.041	0.20
mango	PLS vs KAN	0.741	0.80
mango	PLS vs KAN intrinsic (projected)	−0.086	0.00
mango	SVR vs RF	0.073	0.20
mango	SVR vs MLP	0.395	0.00
mango	SVR vs CNN	0.065	0.00
mango	SVR vs KAN	0.619	0.60
mango	SVR vs KAN intrinsic (projected)	0.141	0.20
mango	RF vs MLP	0.196	0.40
mango	RF vs CNN	0.103	0.20
mango	RF vs KAN	0.261	0.20
mango	RF vs KAN intrinsic (projected)	0.313	0.40
mango	MLP vs CNN	0.119	0.20
mango	MLP vs KAN	0.484	0.00
mango	MLP vs KAN intrinsic (projected)	0.164	0.20
mango	CNN vs KAN	0.085	0.20
mango	CNN vs KAN intrinsic (projected)	0.003	0.00
mango	KAN vs KAN intrinsic (projected)	0.048	0.20

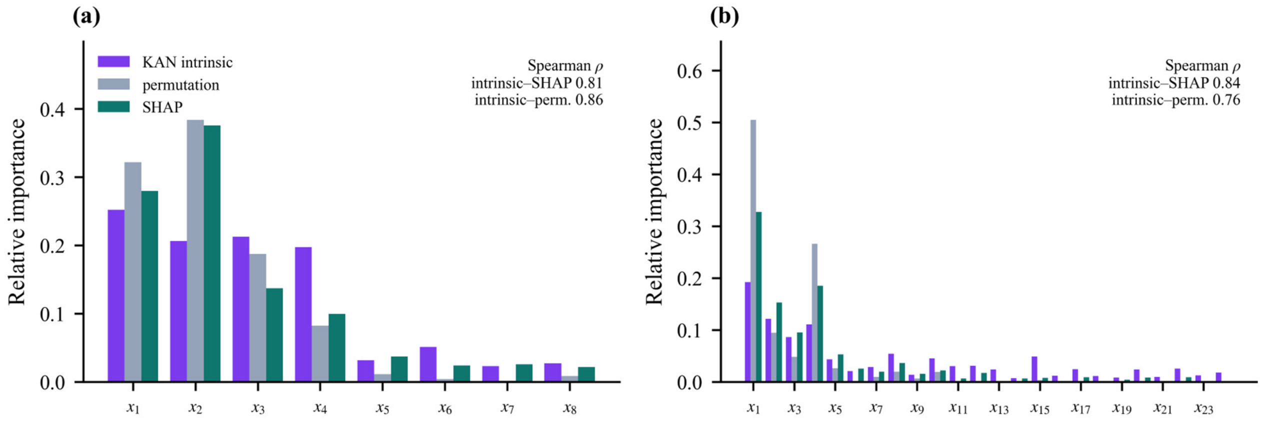

Figure S8. Variable importance from three sources on the same fitted network: the network's own intrinsic importance, permutation importance, and SHAP. (a) Tahini, eight latent variables; (b) mango, twenty-four — the latent width of each dataset's own accuracy configuration, which is the model this comparison is about. Importances are normalised to sum to one within each method so that the orderings can be compared; the Spearman correlations printed in each panel are the middle-budget values of Table S12, computed in the same run.

S12. The Corrected Significance Tests in Full

Section 2.5 of the article states that pairwise model comparisons are tested with the corrected resampled *t*-test of Nadeau and Bengio [45] and that each family of such tests is

adjusted with the Holm–Bonferroni procedure [46]. Table S14 lists every such test behind Sections 3.3–3.6 of the article except those of the parameter-matched comparison, which are given in Section S24; Table S15 gives the detection-limit comparison, which requires a different test and is described below. The article quotes the adjusted p-values it uses; these tables give the complete families, so that a reader can see what was tested and not only what was reported. The comparisons of the KAN with each of the other five models quoted in Section 3.2 of the article form a separate family and are not included in Table S14; they are tested on the cross-validation folds of the training partition and Holm-adjusted over the five comparisons on each matrix. On tahini none is significant (smallest adjusted $p = 0.36$, against PLS); on mango only the difference from the random forest is significant (+0.036, adjusted $p = 0.04$).

Two designs, two variance corrections. The Nadeau–Bengio correction inflates the naive paired variance by $(1/k + \varrho)$, with $\varrho = n_{\text{test}}/n_{\text{train}}$, because resampled training partitions overlap. The comparisons in Table S14 come from two designs, and the family name identifies which: the edge-basis and glass-box families repeat a fit on one fixed external partition, so their unit is the seed, while the equation-under-shift family is leave-one-group-out and its unit is the withheld fold. The edge-basis and glass-box families repeat a fit on one fixed external partition, varying only the training seed, so ϱ is the test-to-training size ratio of that partition (0.356 for tahini, 0.141 for mango). In the equation-under-shift family each fold withholds one production lot or one harvest season, so ϱ is the mean ntest/ntrain over the folds.

Two limits on what these tests can support should be stated explicitly. First, where the design is repeated seeds on a fixed partition, the test asks whether a difference exceeds run-to-run variation; it does not ask whether the difference would survive a different test set, because the test rows never change. Second, the glass-box family has three seeds and therefore two residual degrees of freedom, and one of its models is deterministic, while the boosting machines vary by less than 0.002 across seeds. A difference in that family is therefore significant when it is consistent across the three runs rather than when it is large. A non-significant result in that family provides very little evidence.

What the tests find. Of the 78 tests in Table S14, four reach significance after adjustment, and two of these four express the same finding on the two matrices: the Fourier basis is worse than the basis selected by inner cross-validation, by 0.075 on tahini's named bands (adjusted $p = 0.022$) and by 0.071 on mango's latent scores ($p = 0.006$). The other two are on tahini's latent scores, where the printed equation exceeds gradient boosting (+0.040, $p = 0.004$) and the plain boosting machine (+0.009, $p = 0.0497$). No other comparison — between extraction routes, between the equation and the network from which it was derived, within the shift family or within the mango glass-box family — is significant at this power. The two largest differences in the table are both among the least significant, and for the same reason. The printed equation exceeds genetic-programming symbolic regression by 0.995 R^2 on mango's latent scores at an adjusted p of 0.51, and the exact route exceeds the conversion by 0.665 R^2 on mango's named bands at an adjusted p of 1.00 — in each case because the losing arm's spread (SD 0.46 and 1.19) is larger than the scale the difference is measured on. Where the article reports a numerical ordering without a claim, this table explains why no claim is made; a non-significant result here is not evidence that the compared models are equivalent.

The detection limit needs a different test. The out-of-fold detection limit is one number per model rather than a per-fold vector, so a paired t-test does not apply to it. It is instead tested with the paired cluster bootstrap that produced its intervals: the 2000 draws of the out-of-fold analysis are regenerated identically, every model is scored on the same draws, and the difference between two models is computed draw by draw, which gives an interval and a two-sided bootstrap p-value for the difference itself rather than for each

limit separately. The regenerated draws reproduce all six published point estimates and both interval endpoints exactly before any difference is reported. Five of the fifteen pairs differ significantly after adjustment, and all five involve support-vector regression, whose limit of 21.6% adulterant is separated from those of all five other models. No other pair can be distinguished, including PLS and the KAN, whose limits differ by 5.3 percentage points (95% CI −8.6 to −0.03, adjusted $p = 0.40$). The ordering reported in Section 3.2 of the article is therefore a ranking of point estimates, not a demonstration that the models differ, and the article presents it as such.

Table S14. Corrected resampled t-tests, Holm-adjusted within each family, for every corrected test behind Sections 3.3–3.6 of the article. The twelve-cell parameter-matched comparison of Section 3.6 is tested separately, paired by seed (Section S24); its seed-stability comparison carries no test and is reported descriptively, as standard deviations. "n" is the number of paired units — seeds for the edge-basis and glass-box families, which repeat a fit on one fixed external partition, and withheld folds for the equation-under-shift family, which is leave-one-group-out; the variance correction uses the ϱ implied by each design, as described above. A positive mean difference favours model A. Adjusted p-values are given to three decimal places, and significance is decided on the unrounded value: the comparison shown as 0.050 is 0.0497, and the two shown as 0.052 are 0.0516 and 0.0521. The families are adjusted separately because they answer separate questions; adjusting all 78 together would be too conservative to be informative.

Family and setting	Model A	Model B	n	Mean difference (A − B)	SD of difference	Holm-adjusted p	Significant at 0.05
Edge basis: tahini, latent scores, R^2	Chebyshev basis	B-spline control	5	+0.0036	0.0047	1.000	No
Edge basis: tahini, latent scores, R^2	Chebyshev basis	Fourier basis	5	+0.0460	0.0135	0.052	No
Edge basis: tahini, latent scores, R^2	Chebyshev basis	power basis	5	+0.0034	0.0045	1.000	No
Edge basis: tahini, latent scores, R^2	Chebyshev basis	pykan conversion	5	−0.0011	0.0056	1.000	No
Edge basis: tahini, latent scores, R^2	Chebyshev basis	Gaussian basis	5	+0.0036	0.0109	1.000	No
Edge basis: tahini, latent scores, fidelity	Chebyshev basis	Fourier basis	5	−0.0000	0.0000	1.000	No
Edge basis: tahini, latent scores, fidelity	Chebyshev basis	power basis	5	−0.0000	0.0000	1.000	No
Edge basis: tahini, latent scores, fidelity	Chebyshev basis	pykan conversion	5	+0.0170	0.0064	0.094	No
Edge basis: tahini, latent scores, fidelity	Chebyshev basis	Gaussian basis	5	−0.0000	0.0000	1.000	No
Edge basis: tahini, named bands, R^2	power basis	B-spline control	5	−0.0015	0.0028	1.000	No
Edge basis: tahini, named bands, R^2	power basis	Chebyshev basis	5	+0.0074	0.0094	1.000	No

Family and setting	Model A	Model B	n	Mean difference (A − B)	SD of difference	Holm-adjusted p	Significant at 0.05
Edge basis: tahini, named bands, R^2	power basis	Fourier basis	5	+0.0746	0.0172	0.022	Yes
Edge basis: tahini, named bands, R^2	power basis	pykan conversion	5	+0.0014	0.0057	1.000	No
Edge basis: tahini, named bands, R^2	power basis	Gaussian basis	5	−0.0016	0.0026	1.000	No
Edge basis: tahini, named bands, fidelity	power basis	Chebyshev basis	5	+0.0000	0.0000	0.903	No
Edge basis: tahini, named bands, fidelity	power basis	Fourier basis	5	+0.0000	0.0000	0.570	No
Edge basis: tahini, named bands, fidelity	power basis	pykan conversion	5	+0.2209	0.1724	0.570	No
Edge basis: tahini, named bands, fidelity	power basis	Gaussian basis	5	−0.0000	0.0000	0.903	No
Edge basis: mango, latent scores, R^2	Gaussian basis	B-spline control	5	+0.0033	0.0136	1.000	No
Edge basis: mango, latent scores, R^2	Gaussian basis	Chebyshev basis	5	+0.0021	0.0110	1.000	No
Edge basis: mango, latent scores, R^2	Gaussian basis	Fourier basis	5	+0.0712	0.0148	0.006	Yes
Edge basis: mango, latent scores, R^2	Gaussian basis	power basis	5	+0.0088	0.0050	0.163	No
Edge basis: mango, latent scores, R^2	Gaussian basis	pykan conversion	5	+0.0182	0.0115	0.163	No
Edge basis: mango, latent scores, fidelity	Gaussian basis	Chebyshev basis	5	+0.0000	0.0000	0.516	No
Edge basis: mango, latent scores, fidelity	Gaussian basis	Fourier basis	5	+0.0000	0.0000	0.363	No
Edge basis: mango, latent scores, fidelity	Gaussian basis	power basis	5	+0.0000	0.0000	0.697	No
Edge basis: mango, latent scores, fidelity	Gaussian basis	pykan conversion	5	+0.0467	0.0205	0.071	No
Edge basis: mango, named bands, R^2	Chebyshev basis	B-spline control	5	−0.0002	0.0316	1.000	No

Family and setting	Model A	Model B	n	Mean difference (A − B)	SD of difference	Holm-adjusted p	Significant at 0.05
Edge basis: mango, named bands, R^2	Chebyshev basis	Fourier basis	5	+0.0875	0.0341	0.059	No
Edge basis: mango, named bands, R^2	Chebyshev basis	power basis	5	−0.0432	0.0227	0.125	No
Edge basis: mango, named bands, R^2	Chebyshev basis	pykan conversion	5	+0.6647	1.1466	1.000	No
Edge basis: mango, named bands, R^2	Chebyshev basis	Gaussian basis	5	−0.0025	0.0213	1.000	No
Edge basis: mango, named bands, fidelity	Chebyshev basis	Fourier basis	5	−0.0000	0.0000	1.000	No
Edge basis: mango, named bands, fidelity	Chebyshev basis	power basis	5	+0.0000	0.0000	1.000	No
Edge basis: mango, named bands, fidelity	Chebyshev basis	pykan conversion	5	+0.8046	1.4970	1.000	No
Edge basis: mango, named bands, fidelity	Chebyshev basis	Gaussian basis	5	−0.0000	0.0000	1.000	No
Equation under shift: mango, latent scores, R^2	converted equation	B-spline network	4	+0.0109	0.0222	1.000	No
Equation under shift: mango, latent scores, R^2	exact equation	elementary network	4	+0.0001	0.0005	1.000	No
Equation under shift: mango, latent scores, R^2	exact equation	converted equation	4	−0.0044	0.0427	1.000	No
Equation under shift: mango, latent scores, R^2	exact equation	OLS	4	−0.0039	0.0264	1.000	No
Equation under shift: mango, latent scores, R^2	converted equation	OLS	4	+0.0005	0.0176	1.000	No
Equation under shift: mango, latent scores, R^2	elementary network	B-spline network	4	+0.0064	0.0223	1.000	No
Equation under shift: mango, latent scores, fidelity	exact equation	converted equation	4	+0.0563	0.0227	0.052	No

Family and setting	Model A	Model B	n	Mean difference (A − B)	SD of difference	Holm-adjusted p	Significant at 0.05
Equation under shift: mango, named bands, R^2	converted equation	B-spline network	4	+0.0002	0.0526	1.000	No
Equation under shift: mango, named bands, R^2	exact equation	elementary network	4	+0.0000	0.0016	1.000	No
Equation under shift: mango, named bands, R^2	exact equation	converted equation	4	+0.0315	0.0329	1.000	No
Equation under shift: mango, named bands, R^2	exact equation	OLS	4	+0.0062	0.0524	1.000	No
Equation under shift: mango, named bands, R^2	converted equation	OLS	4	−0.0253	0.0469	1.000	No
Equation under shift: mango, named bands, R^2	elementary network	B-spline network	4	+0.0317	0.0333	1.000	No
Equation under shift: mango, named bands, fidelity	exact equation	converted equation	4	+0.0974	0.0829	0.235	No
Equation under shift: tahini, latent scores, R^2	converted equation	B-spline network	5	−0.0082	0.0107	1.000	No
Equation under shift: tahini, latent scores, R^2	exact equation	elementary network	5	−0.0001	0.0003	1.000	No
Equation under shift: tahini, latent scores, R^2	exact equation	converted equation	5	−0.0273	0.0610	1.000	No
Equation under shift: tahini, latent scores, R^2	exact equation	OLS	5	−0.0266	0.0595	1.000	No
Equation under shift: tahini, latent scores, R^2	converted equation	OLS	5	+0.0006	0.0172	1.000	No
Equation under shift: tahini, latent scores, R^2	elementary network	B-spline network	5	−0.0353	0.0655	1.000	No

https://doi.org/10.3390/foods15203639

Family and setting	Model A	Model B	n	Mean difference (A – B)	SD of difference	Holm-adjusted p	Significant at 0.05
Equation under shift: tahini, latent scores, fidelity	exact equation	converted equation	5	+0.0435	0.0498	0.263	No
Equation under shift: tahini, named bands, R^2	converted equation	B-spline network	5	−0.0301	0.0301	1.000	No
Equation under shift: tahini, named bands, R^2	exact equation	elementary network	5	+0.0004	0.0006	1.000	No
Equation under shift: tahini, named bands, R^2	exact equation	converted equation	5	+0.0308	0.0292	1.000	No
Equation under shift: tahini, named bands, R^2	exact equation	OLS	5	+0.0378	0.0386	1.000	No
Equation under shift: tahini, named bands, R^2	converted equation	OLS	5	+0.0070	0.0387	1.000	No
Equation under shift: tahini, named bands, R^2	elementary network	B-spline network	5	+0.0002	0.0101	1.000	No
Equation under shift: tahini, named bands, fidelity	exact equation	converted equation	5	+0.0642	0.0670	0.226	No
Glass-box rivals: mango, latent scores, R^2	KAN equation (Gaussian basis)	boosting machine	3	+0.0129	0.0058	0.510	No
Glass-box rivals: mango, latent scores, R^2	KAN equation (Gaussian basis)	boosting machine + interactions	3	+0.0012	0.0062	1.000	No
Glass-box rivals: mango, latent scores, R^2	KAN equation (Gaussian basis)	spline additive model	3	+0.0108	0.0054	0.510	No
Glass-box rivals: mango, latent scores, R^2	KAN equation (Gaussian basis)	reference perceptron	3	−0.0288	0.0068	0.176	No
Glass-box rivals: mango, latent scores, R^2	KAN equation (Gaussian basis)	symbolic regression	3	+0.9950	0.4598	0.510	No

Family and setting	Model A	Model B	n	Mean difference (A − B)	SD of difference	Holm-adjusted p	Significant at 0.05
Glass-box rivals: mango, latent scores, R^2	KAN equation (Gaussian basis)	gradient boosting	3	+0.0215	0.0109	0.510	No
Glass-box rivals: mango, latent scores, R^2	KAN equation (Gaussian basis)	spline-activation perceptron	3	+0.0010	0.0097	1.000	No
Glass-box rivals: tahini, latent scores, R^2	KAN equation	boosting machine	3	+0.0091	0.0010	0.050	Yes
Glass-box rivals: tahini, latent scores, R^2	KAN equation	boosting machine + interactions	3	+0.0016	0.0014	0.640	No
Glass-box rivals: tahini, latent scores, R^2	KAN equation	spline additive model	3	+0.0069	0.0009	0.057	No
Glass-box rivals: tahini, latent scores, R^2	KAN equation	reference perceptron	3	+0.0009	0.0039	0.812	No
Glass-box rivals: tahini, latent scores, R^2	KAN equation	symbolic regression	3	+0.4994	0.3337	0.640	No
Glass-box rivals: tahini, latent scores, R^2	KAN equation	gradient boosting	3	+0.0398	0.0012	0.004	Yes
Glass-box rivals: tahini, latent scores, R^2	KAN equation	spline-activation perceptron	3	−0.0042	0.0009	0.108	No

Table S15. Pairwise differences between the out-of-fold detection limits, from the paired cluster bootstrap described above. A negative difference means model A has the lower limit. Intervals are 2.5th and 97.5th percentiles of the difference over 2000 draws; p-values are two-sided bootstrap values, Holm-adjusted over the fifteen pairs.

Model A	Model B	LOD A (%)	LOD B (%)	Difference (percentage points)	95% CI of the difference	Holm-adjusted p	Significant at 0.05
PLS	SVR	8.93	21.59	−12.66	−16.41 to −9.55	0.007	Yes
PLS	RF	8.93	13.11	−4.18	−9.78 to +1.35	0.810	No
PLS	MLP	8.93	10.32	−1.39	−4.64 to +2.83	1.000	No
PLS	CNN	8.93	14.88	−5.95	−9.93 to −1.92	0.150	No
PLS	KAN	8.93	14.27	−5.34	−8.58 to −0.03	0.400	No
SVR	RF	21.59	13.11	+8.48	+2.31 to +14.82	0.022	Yes
SVR	MLP	21.59	10.32	+11.27	+10.54 to +14.68	0.007	Yes
SVR	CNN	21.59	14.88	+6.71	+3.56 to +12.93	0.007	Yes
SVR	KAN	21.59	14.27	+7.32	+5.43 to +14.38	0.007	Yes
RF	MLP	13.11	10.32	+2.79	−3.45 to +10.35	1.000	No
RF	CNN	13.11	14.88	−1.77	−8.77 to +7.47	1.000	No
RF	KAN	13.11	14.27	−1.16	−8.31 to +6.62	1.000	No
MLP	CNN	10.32	14.88	−4.56	−8.74 to −1.05	0.150	No
MLP	KAN	10.32	14.27	−3.95	−6.21 to +0.63	0.400	No
CNN	KAN	14.88	14.27	+0.61	−2.43 to +7.01	1.000	No

S13. Extended Methods, and the Detection Limit in Full

This section gives the procedural details and derivations that the article summarises. It introduces no new results: every number here is reported in the article or in Tables 4, 7 and 8.

Training budgets. Optimisation used Adam at a learning rate of 5 × 10^{-3} with mini-batches of 128, for 300 steps in the accuracy configuration and 250 in the equation-bearing one, followed after pruning by a further 60. Two later experiments use longer training. The named-band comparison of Section 3.4, which fits on a handful of selected channels, uses 400 steps followed by 100 after pruning, while its inner search retains the published 250 and 60. The edge-basis comparison of Section 3.3 is run on both the full latent set and the selected bands. There, the elementary-basis networks are budgeted in epochs rather than steps — 180, plus 72 after pruning — so that the ten-times-larger mango partition is not silently undertrained; the pykan reference arm against which they are compared uses the same 400 and 100 as the named-band experiment.

The additional baselines of Section 3.6. Because a depth-one Kolmogorov–Arnold network *is* a generalised additive model, the explainable boosting machine and a spline GAM were added as older members of the same transparency class. Gradient boosting and a plain perceptron of fixed architecture were added as opaque references, and genetic-programming symbolic regression as the most direct alternative route to a closed-form expression. Finally, a perceptron whose node activations are learnable B-splines on the KAN's own grid was included for two reasons: the controlled architecture literature attributes the KAN's advantage on symbolic tasks to the basis rather than to the edge-wise structure, and a spline on the node, applied after the inputs have been mixed, yields no closed form in the original variables. The reference perceptron is a fixed two-hidden-layer network with batch normalisation and dropout fitted on the KAN's latent width; it is not the tuned benchmark perceptron of Table 3, which is why its mango score differs from the one in that table.

Attribution budgets. SHAP is evaluated at three budgets — background sets of 50, 100 and 200 spectra; 150, 300 and 600 evaluation rows; 100, 200 and 400 coalition samples — so that the convergence of a rank correlation is shown rather than assumed (Section S11).

Why the first detection limit was too optimistic, in detail. A detection limit states how far a prediction must depart from the authentic population before the departure becomes meaningful, so it is only as reliable as the description of that population. Exactly one of the five authentic tahini lots falls on the test side of the published split, so the blank dispersion σ is the scatter of a single lot. A cluster bootstrap that resamples whole physical samples therefore redraws the same 151 scans of that lot in every resample: only 1267 of its 2000 draws contained any authentic material at all, and the remaining 733 were discarded. The out-of-fold estimate pools predictions over three independent grouped five-fold partitions of all 55 samples, so that each lot serves as a blank while it is held out and lot-to-lot variability enters σ directly. The bootstrap of the out-of-fold estimate resamples whole lots for σ and whole samples for the slope; the estimator, preprocessing and model configurations are unchanged.

The re-estimated limits are 8.93% adulterant for PLS (95% CI 4.57–11.13), 10.32% for the MLP (3.60–13.96), 14.27% for the KAN (5.04–18.22) and 14.88% for the CNN (7.15–19.51); the limit of support-vector regression changes little, from 19.61% to 21.59%, whereas that of the random forest improves from 22.40% to 13.11% (Table 4). The KAN's limit rises by a factor of 4.1, the largest of the six, against 3.1 for the MLP and 2.6 for PLS. Its per-repeat limits are also the most scattered — 27.02%, 11.93% and 12.56% across the three partitions, against 10.31%, 7.53% and 9.59% for PLS — a pattern consistent with the

frozen-design results of Section S4, although, with selection nested inside each fold, the KAN was the least variable model across lots.

The anchored-band comparison of Section 3.4, in full. The eight bands fixed in advance from the literature are 3008, 2922, 2853, 1743, 1653, 1465, 1163 and 722 cm^{-1}, each taken at the nearest measured channel. Every model lost accuracy relative to its twelve-band result, and all but one relative to its sixteen-band result. The exception is the KAN (3,) equation, which rises from 0.9034 ± 0.1836 to 0.9263 ± 0.0264, a rise that reflects its own instability at sixteen bands more than any property of the anchored set. In the inverted ordering the additive glass-boxes led — the boosting machine with interactions at 0.9608, the spline GAM at 0.9607, the plain boosting machine at 0.9560 and the additive KAN spline network at 0.9549 — while ordinary least squares, PLS and ridge all reached 0.936. On the anchored bands, the additive form falls from first to last of the three KAN arms (0.9162 ± 0.0314 against 0.9279 ± 0.0257 and 0.9263 ± 0.0264); the margins are small, and all three arms are unstable. The KAN (3,) arm loses more to the conversion at sixteen data-selected bands (0.083) than the additive form loses on the anchored eight (0.039), and a single tahini extraction in Section 3.3 lost about a whole unit of R^2.

Depth on the named bands. Adding a hidden layer changed the equation's R^2 from 0.9895 to 0.9503 ± 0.0584 at a hidden width of two and to 0.9034 ± 0.1836 at a width of three, with fidelity falling from 0.994 to 0.845. Readers who want the shortest formula should use the one-hidden-layer arm; those who want to read a per-band response should use the additive arm. The deeper variants are reported for completeness.

S14. Artefact Controls for the Selected Tahini Channels

Four of the eight channels in Equation (3) (1502, 1487, 1447 and 1140 cm^{-1}; four of the five unassigned channels of Section 3.4 of the article and Table S11) fall outside every band tabulated in the sources we cite, and the two spectral windows that lead every model's attribution (near 1490 and 1235 cm^{-1}, Section S11) are also untabulated. A channel can be selected for reasons unrelated to composition: instrument drift between measurement sessions, differences in baseline or crystal contact, residual atmospheric water vapour, or noise. We therefore tested each selected channel against these explanations directly on the released spectra (scripts revision/r07_band_artifact_controls.py and r07b_band_confound_checks.py). The tests and their results are summarised in Table S16. Each test is computed on sample means (the average of the replicate scans of one physical sample) after standard normal variate correction, the same preprocessing the models use.

Composition dependence. If a channel carried only drift or noise, its absorbance would not vary systematically with the adulterant fraction. Along each dilution series (the five authentic lots together with the 25 blends of one adulterant, 30 samples), we computed the Spearman correlation between absorbance and adulterant fraction and its permutation p-value (2000 permutations). Every channel in Equation (3) varies monotonically with composition in at least one series ($|\varrho| = 0.81$–0.99 in the stronger series, $p \leq 0.0005$). The two leading windows are among the most strongly composition-dependent channels in the spectrum: at 1235 cm^{-1}, $\varrho = -0.98$ for both adulterants (96.8th and 99.4th percentiles of all 1762 channels). The responses are adulterant-specific: 1140 cm^{-1} responds to peanut paste ($\varrho = -0.95$) but not to sunflower paste ($\varrho = -0.08$, $p = 0.67$), whereas 989 cm^{-1} responds more strongly to sunflower paste ($\varrho = +0.98$) than to peanut paste ($\varrho = +0.57$).

Replication within each production lot. Each production lot was prepared and measured as a batch together with its own ten blends. A batch or session artefact would therefore not reproduce the same composition response inside every lot. We fitted the slope of absorbance on adulterant fraction separately within each lot. For every channel in Equation (3) and every series with a significant pooled response, the sign of the slope agrees in all five lots, with one exception: 1286 cm^{-1} with peanut paste, the weakest such response

(ϱ = −0.53; 4/5 lots) (Table S16). For 1140 cm^{-1} with sunflower paste, which shows no pooled response, the sign agrees in 3/5 lots.

Water vapour and lot. Residual atmospheric water vapour adds narrow rotational lines to mid-infrared spectra, in the bending region between about 1300 and 1900 cm^{-1} and in the stretching region above 3500 cm^{-1}, and varies from scan to scan with the purge state. We built a per-scan vapour index from the stretching region (3700–3900 cm^{-1}), where the pastes themselves show little absorption. The replicate-to-replicate variation at 1502 and 1487 cm^{-1} does co-vary with this index (Spearman ϱ = 0.23 and 0.22, the 98th percentile of all channels), so part of the scan-level noise at these two channels is attributable to water vapour. The vapour index also correlated with the sunflower fraction (ϱ = −0.52, $p = 0.004$; peanut series ϱ = −0.32, $p = 0.09$) and differed between lots (Kruskal–Wallis $p = 0.04$). Measurement conditions were therefore not completely independent of composition, and we report this as a limitation. This dependence does not, however, explain the selection: when the composition effect is re-estimated with the vapour index and the production lot as covariates, the composition coefficient at every channel in Equation (3) is essentially unchanged (for example, at 1502 cm^{-1} from −0.024 to −0.026 standardised absorbance units per % peanut paste, and at 1487 cm^{-1} from −0.028 to −0.029) and remains highly significant ($p < 10^{-3}$ in every series where the channel responds).

Selection under shuffled labels. Variable importance in projection (VIP) can favour channels with large spectral variance regardless of the target. When rerun, the published selection procedure (12 PLS components, 15 cm^{-1} minimum separation) reproduces the published sixteen channels exactly; we repeated it 200 times with the composition labels shuffled between physical samples. Channels 1502, 1242 and 989 cm^{-1} were selected in 0%, 1.5% and 2% of shuffled runs, and 1180, 1447 and 1487 cm^{-1} in 8–16%. Two channels were selected often even without true labels, 1286 cm^{-1} (25%) and 1140 cm^{-1} (37%); their selection partly reflects the variance structure of the spectra rather than composition, although both show a clear composition response (above). All eight channels of Equation (3) were selected in all five leave-one-lot-out training sets.

What the calibration loses without the vapour-affected channels. Removing 1502, 1487 and 1472 cm^{-1} from the sixteen-band ordinary least-squares calibration lowers its external R^2 from 0.9923 to 0.9887; removing 1502 and 1487 cm^{-1} from the eight channels of Equation (3) lowers the external R^2 of ordinary least squares on those channels from 0.9894 to 0.9864. The calibration therefore depends only marginally on these two channels.

Interpretation and limitations. These tests show that the selected channels carry composition information that replicates within every production lot, is not explained by the measured water-vapour index or by production lot, and exceeds the replicate-scan noise. They do not assign the channels to a vibrational mode. The candidates are components that differ between sesame, peanut and sunflower pastes, rather than the shared triglyceride matrix, such as aromatic compounds, carbohydrates and proteins, whose amide III region spans about 1230–1300 cm^{-1} [50]. We report the channels as composition-dependent but unassigned where no verified assignment exists.

A candidate, not an assignment. Two of the channels in Equation (3), 1502 and 1487 cm^{-1}, lie within about 2 cm^{-1} of the most intense band (1500 cm^{-1}) and its reported shoulder (near 1488 cm^{-1}) in the experimental infrared spectrum of pure sesamin [62]. Sesamin and sesamolin are the characteristic lignans of sesame; their combined content in commercial virgin and roasted sesame oils has been reported at 444–1601 mg/100 g, and total lignans in sesame products including tahini at 11–763 mg/100 g [63], whereas sunflower and peanut are not natural sources, although traces have been found in their oils as a result of cross-contact during processing [64]. A sesame-specific absorber would explain why both channels decrease with either adulterant, as expected of a diluted sesame constituent, and do so in every lot. We report this proximity only as a hypothesis: the sesamin spectrum

was recorded on the pure solid in KBr rather than by ATR on a paste, sesamin is a minor constituent of tahini, and aromatic and protein absorptions also occur in this region, so the channels remain unassigned.

Table S16. Artefact controls for the channels of Equation (3) and the two leading attribution windows. ϱ: Spearman correlation between sample-mean SNV absorbance and adulterant fraction along each dilution series (n = 30 samples; permutation $p \leq 0.0005$ unless stated). Lots agreeing: number of production lots in which the within-lot slope has the same sign as the pooled response. Coefficient, peanut / sunflower: change in standardised sample-mean absorbance per % adulterant, alone → adjusted for the vapour index and production lot; n.s., adjusted $p \geq 0.05$. Selected under shuffled labels: fraction of 200 label-shuffled selections that picked the channel. Selected in leave-one-lot-out: fraction of the five leave-one-lot-out training sets that selected it. —, not computed for the windows.

Channel (cm^{-1})	ϱ, peanut series	ϱ, sunflower series	Lots agreeing (peanut / sunflower)	Coefficient, peanut (alone → adjusted)	Coefficient, sunflower (alone → adjusted)	Selected under shuffled labels	Selected in leave-one-lot-out
1502	−0.78	−0.81	5/5 / 5/5	−0.024 → −0.026	−0.023 → −0.028	0.000	1.0
1487	−0.93	−0.95	5/5 / 5/5	−0.028 → −0.029	−0.029 → −0.030	0.155	1.0
1447	−0.92	−0.94	5/5 / 5/5	−0.027 → −0.028	−0.028 → −0.028	0.115	1.0
1286	−0.53 (p = 0.003)	+0.84	4/5 / 5/5	−0.018 → −0.014	+0.027 → +0.031	0.250	1.0
1242	−0.98	−0.99	5/5 / 5/5	−0.029 → −0.029	−0.029 → −0.027	0.015	1.0
1180	−0.91	−0.97	5/5 / 5/5	−0.027 → −0.027	−0.028 → −0.025	0.080	1.0
1140	−0.95	−0.08 (p = 0.667)	5/5 / 3/5	−0.028 → −0.028	n.s.	0.370	1.0
989	+0.57 (p = 0.002)	+0.98	5/5 / 5/5	+0.017 → +0.020	+0.030 → +0.029	0.020	1.0
window ~1490	−0.90	−0.92	5/5 / 5/5	—	—	—	—
window ~1235	−0.98	−0.98	5/5 / 5/5	—	—	—	—

S15. Number of Channels and Band Overlap

Two questions follow from calibrating on a few selected channels rather than on the full spectrum: whether the number of channels is sufficient, and whether selecting single channels is fragile when neighbouring bands overlap. We answered both with a linear control (ordinary least squares on the selected channels), so that the result measures the information in the channels and not the capacity of a particular model (script revision/r12_band_count_overlap.py). For k = 2, 3, 4, 6, 8, 10, 12, 16, 20, 24, 30 and 40 channels, the channels were ranked by VIP inside each fold of a grouped five-fold cross-validation on the training partition, and separately on the whole training partition for the external test. Each selection was used in two forms: the absorbance at the single channel, and the mean absorbance over a ±7.5 cm^{-1} window centred on it (its width equals the published minimum separation, so the windows do not overlap), which integrates across the local band shape (Table S17).

Accuracy rises quickly and then saturates. The cross-validated R^2 is 0.931 with three channels, 0.978 with eight and 0.980 with sixteen, and reaches 0.989 only with forty. On the external test, sixteen channels give 0.9923 against 0.9943 for PLS on the full 1762-channel spectrum. Beyond eight to sixteen channels, additional channels add at most about one hundredth of R^2. With eight channels or more, integrating each channel over its band window raises the cross-validated R^2 by 0.002–0.005 and the external R^2 by 0.0005–0.0017;

https://doi.org/10.3390/foods15203639

with two to four channels the effect of integration is mixed and small (cross-validated R^2 −0.011 to +0.001; external R^2 −0.006 to −0.0002). Selecting single channels is therefore not fragile when neighbouring bands overlap, and averaging over the band gives a small gain, consistent with noise reduction.

Interpretation and limitations. A linear calibration on eight to sixteen VIP-selected channels retains nearly all of the accuracy of the full spectrum, and band integration changes R^2 by at most about 0.01. The comparison rests on one training partition and a single external test set, only a ±7.5 cm^{-1} window was examined, and the differences between numbers of channels and between single and band-integrated forms were not tested.

Table S17. Accuracy of ordinary least squares for the tahini fraction on k VIP-selected channels, used as single channels and as band-integrated windows (±7.5 cm^{-1}). Cross-validation is grouped five-fold on the training partition, with selection repeated inside each fold; the external test is the group-independent test set of Table 2. Inner CV: mean ± SD over the five folds. Last row: PLS (12 latent variables) on all 1762 channels, external test only.

Channels (k)	Inner CV R^2, single channel	Inner CV R^2, band-integrated	External R^2, single channel	External R^2, band-integrated
2	0.906 ± 0.056	0.895 ± 0.067	0.9688	0.9628
3	0.931 ± 0.029	0.932 ± 0.030	0.9781	0.9779
4	0.876 ± 0.125	0.875 ± 0.128	0.9838	0.9829
6	0.955 ± 0.038	0.959 ± 0.030	0.9879	0.9895
8	0.978 ± 0.006	0.981 ± 0.006	0.9894	0.9907
10	0.978 ± 0.008	0.980 ± 0.006	0.9900	0.9917
12	0.976 ± 0.007	0.981 ± 0.005	0.9910	0.9926
16	0.980 ± 0.005	0.984 ± 0.004	0.9923	0.9931
20	0.983 ± 0.005	0.986 ± 0.005	0.9916	0.9925
24	0.984 ± 0.005	0.987 ± 0.004	0.9923	0.9930
30	0.985 ± 0.005	0.988 ± 0.003	0.9926	0.9933
40	0.989 ± 0.000	0.991 ± 0.001	0.9956	0.9961
1762 (full spectrum, PLS, 12 LV)	—	—	0.9943	—

S16. Low-Level Identification and False Positives on Authentic Tahini

Each production lot contains its own interleaved set of adulteration levels (T1: 4, 24, 44, 64 and 84%; T2: 8, 28, 48, 68 and 88%; and so on to T5: 20, 40, 60, 80 and 100%) for both adulterants, together with its own authentic tahini. In leave-one-lot-out validation, every level of both adulterants, including 4, 8 and 12%, and every authentic sample is therefore predicted exactly once by a model that has not seen that lot. We used the out-of-fold predictions of the nested leave-one-lot-out design (Section S4; configuration re-selected inside each outer training set), averaged over model seeds, and repeated every analysis on the frozen design (published configurations held fixed) for comparison. No model was refitted for this section (script revision/r03_low_level_authentic_bootstrap.py).

Three quantities were computed for each spectrum. Identification: the spectrum is assigned to the adulterant (peanut or sunflower) with the larger predicted fraction. Detection: the spectrum is flagged when the predicted tahini fraction falls below the detection threshold. The threshold was re-chosen inside each outer training set with the published rule (PLS, grouped three-fold cross-validation, maximum F1), so the held-out lot was never used to set it; the resulting thresholds were 92.5, 89.0, 91.5, 92.5 and 93.0% tahini for T1–T5. Quantification: the predicted adulterant fraction (100 minus predicted tahini) and its absolute error. Each level of each adulterant is a single physical sample (14–17 replicate

spectra), and each lot contains a single authentic sample (150–151 spectra). Per-level results therefore describe one sample each, and spectrum-level rates within a sample are not independent observations.

Low levels (Table S18). Pooled over 4–12% (six samples), identification accuracy was 0.96 for the MLP, 0.86 for PLS, 0.80 for the KAN, 0.66 for SVR, 0.62 for the CNN and 0.45 for RF, and the detection rate was 1.00 for SVR, 0.98 for PLS, 0.97 for the MLP, 0.85 for RF, 0.68 for the KAN and 0.55 for the CNN. Quantification at these levels was poor for every model. At a true adulterant fraction of 4%, PLS predicted 14.8% (peanut) and 18.7% (sunflower), the MLP 14.5% and 14.6%, and the KAN 6.4% and 8.4%. The 4% level lies below every out-of-fold detection limit of Table 4 and Section S17, and the 8 and 12% levels lie below or near them (Table 4: 8.9–21.6%), so the predicted fractions are not meaningful as quantitative estimates. Nor is a high detection rate at low levels, on its own, evidence of sensitivity: a model with a positive bias on authentic tahini flags low-level blends and authentic samples alike. SVR, which detected every low-level spectrum, flagged four of the five authentic samples (below).

Authentic tahini (Table S19). In the nested design, the mean predicted adulterant fraction on authentic spectra (bias) was 4.3% for the KAN, 5.0% for the CNN, 5.5% for the MLP, 6.9% for PLS, 7.4% for RF and 15.6% for SVR, and the fraction of authentic spectra flagged was 0.16, 0.19, 0.38, 0.39, 0.37 and 0.72, respectively. At the sample level (replicate mean below the lot's threshold), the KAN flagged one authentic sample (T5, 8.6% predicted adulterant), the CNN one (T2, 12.7%), PLS two (T1, 15.3%; T5, 10.3%), the MLP two (T1, 11.7%; T5, 10.3%), RF two (T1, T4) and SVR four (T1, T2, T4, T5). The result depends on the validation design: in the frozen design the KAN also flagged T1 (13.6%), whereas PLS did not (6.5%). The KAN's lower false-positive count in the nested design is therefore associated with the configurations re-selected for the T1 fold rather than with the model class alone. Under the grouped five-fold partitions of Table 4 (script revision/r05_oof_lod.py; authentic bias 3.1% for PLS, 3.9% for the MLP, 4.1% for the CNN, 4.3% for the KAN, 4.7% for RF and 11.3% for SVR), T5 showed the largest or near-largest bias for most models; this points to a property of that authentic sample rather than of any one model.

Interpretation and limitations. Identification of the adulterant at 4–12% was possible for some models, but quantification at those levels was not. Every model produced at least one sample-level false positive on authentic tahini at the per-lot threshold (SVR four of five). With five authentic samples, the sample-level false-positive counts (1/5 to 4/5) are highly uncertain, and differences between models in false-positive rate or identification accuracy were not tested; the comparisons above are rankings of point estimates. These results support positioning the method as a screening tool for material resembling the calibration population, with positive results confirmed by a reference method.

Table S18. Identification, detection and quantification at 4–20% adulterant, nested leave-one-lot-out, tahini. Each single-level row is one physical sample, predicted by models trained without its production lot. Identification accuracy: fraction of spectra assigned to the correct adulterant. Detection rate: fraction of spectra with predicted tahini below the lot's threshold (Table S19). Pooled rows: all spectra at 4–12% (six samples) or at all adulterated levels (50 samples). Lot: production lot of the sample. Spectra: replicate spectra of the sample. Predicted adulterant: 100 minus the predicted tahini fraction, mean ± SD over replicate spectra. Mean absolute error: mean absolute difference between predicted and true adulterant fraction (percentage points). —, not applicable to pooled rows; the detection rate is not given for the 4–100% pooled row.

True adulterant (%)	Adulterant	Lot	Spectra	Model	Predicted adulterant, mean ± SD (%)	Mean absolute error (%)	Identification accuracy	Detection rate
4	peanut	T1	16	PLS	14.8 ± 2.5	10.8	1.00	1.00

https://doi.org/10.3390/foods15203639

True adulterant (%)	Adulterant	Lot	Spectra	Model	Predicted adulterant, mean ± SD (%)	Mean absolute error (%)	Identification accuracy	Detection rate
4	peanut	T1	16	SVR	25.1 ± 4.1	21.1	0.31	1.00
4	peanut	T1	16	RF	16.9 ± 4.6	12.9	0.81	1.00
4	peanut	T1	16	MLP	14.5 ± 1.9	10.5	1.00	1.00
4	peanut	T1	16	CNN	10.2 ± 5.0	6.2	0.56	0.50
4	peanut	T1	16	KAN	6.4 ± 2.4	2.5	1.00	0.25
4	sunflower	T1	16	PLS	18.7 ± 2.9	14.7	1.00	1.00
4	sunflower	T1	16	SVR	31.2 ± 3.1	27.2	1.00	1.00
4	sunflower	T1	16	RF	17.7 ± 4.2	13.7	0.25	1.00
4	sunflower	T1	16	MLP	14.6 ± 2.1	10.6	0.94	1.00
4	sunflower	T1	16	CNN	3.5 ± 2.2	1.9	1.00	0.06
4	sunflower	T1	16	KAN	8.4 ± 3.4	4.4	0.44	0.44
8	peanut	T2	16	PLS	13.7 ± 1.7	5.7	0.19	0.94
8	peanut	T2	16	SVR	18.6 ± 2.8	10.6	0.00	1.00
8	peanut	T2	16	RF	17.7 ± 1.4	9.7	0.00	1.00
8	peanut	T2	16	MLP	13.5 ± 1.6	5.5	0.81	0.94
8	peanut	T2	16	CNN	24.7 ± 1.8	16.7	0.56	1.00
8	peanut	T2	16	KAN	14.4 ± 1.5	6.4	0.50	1.00
8	sunflower	T2	16	PLS	14.2 ± 1.9	6.2	1.00	0.94
8	sunflower	T2	16	SVR	18.3 ± 3.4	10.3	1.00	1.00
8	sunflower	T2	16	RF	13.4 ± 7.3	7.3	0.81	0.62
8	sunflower	T2	16	MLP	14.1 ± 2.0	6.1	1.00	0.88
8	sunflower	T2	16	CNN	23.7 ± 5.4	15.7	0.62	1.00
8	sunflower	T2	16	KAN	14.4 ± 2.0	6.4	1.00	0.94
12	peanut	T3	16	PLS	13.6 ± 1.5	1.8	1.00	1.00
12	peanut	T3	16	SVR	15.6 ± 3.0	4.0	1.00	1.00
12	peanut	T3	16	RF	19.2 ± 2.7	7.2	0.50	1.00
12	peanut	T3	16	MLP	11.1 ± 1.0	1.0	1.00	1.00
12	peanut	T3	16	CNN	11.2 ± 5.1	4.6	0.56	0.62
12	peanut	T3	16	KAN	15.9 ± 2.0	3.9	1.00	1.00
12	sunflower	T3	15	PLS	16.5 ± 1.9	4.5	1.00	1.00
12	sunflower	T3	15	SVR	21.4 ± 7.7	9.5	0.67	1.00
12	sunflower	T3	15	RF	9.8 ± 4.5	4.5	0.33	0.47
12	sunflower	T3	15	MLP	11.7 ± 1.8	1.4	1.00	1.00
12	sunflower	T3	15	CNN	4.0 ± 2.7	8.0	0.40	0.07
12	sunflower	T3	15	KAN	9.5 ± 4.3	4.5	0.87	0.47
16	peanut	T4	16	PLS	16.0 ± 1.1	0.9	1.00	1.00
16	peanut	T4	16	SVR	26.6 ± 2.7	10.6	1.00	1.00
16	peanut	T4	16	RF	23.0 ± 6.9	7.2	1.00	1.00
16	peanut	T4	16	MLP	13.1 ± 1.1	2.9	1.00	1.00
16	peanut	T4	16	CNN	14.4 ± 3.8	3.6	1.00	1.00
16	peanut	T4	16	KAN	21.9 ± 2.7	5.9	1.00	1.00
16	sunflower	T4	16	PLS	8.2 ± 2.0	7.8	0.69	0.75
16	sunflower	T4	16	SVR	18.5 ± 2.6	2.9	0.12	1.00
16	sunflower	T4	16	RF	15.9 ± 2.2	1.8	0.00	1.00
16	sunflower	T4	16	MLP	10.3 ± 1.1	5.7	0.44	1.00
16	sunflower	T4	16	CNN	10.9 ± 3.2	5.1	0.00	0.81
16	sunflower	T4	16	KAN	11.2 ± 1.5	4.8	0.69	1.00
20	peanut	T5	16	PLS	25.7 ± 1.9	5.7	1.00	1.00
20	peanut	T5	16	SVR	24.6 ± 1.5	4.6	0.94	1.00
20	peanut	T5	16	RF	22.1 ± 1.7	2.1	0.31	1.00
20	peanut	T5	16	MLP	26.9 ± 3.4	6.9	1.00	1.00

True adulterant (%)	Adulterant	Lot	Spectra	Model	Predicted adulterant, mean ± SD (%)	Mean absolute error (%)	Identification accuracy	Detection rate
20	peanut	T5	16	CNN	26.6 ± 3.4	6.6	0.81	1.00
20	peanut	T5	16	KAN	21.7 ± 1.1	1.7	1.00	1.00
20	sunflower	T5	16	PLS	27.1 ± 1.5	7.1	1.00	1.00
20	sunflower	T5	16	SVR	29.7 ± 1.2	9.7	1.00	1.00
20	sunflower	T5	16	RF	31.8 ± 12.4	15.2	1.00	1.00
20	sunflower	T5	16	MLP	31.7 ± 1.9	11.7	1.00	1.00
20	sunflower	T5	16	CNN	24.0 ± 10.5	10.7	0.94	1.00
20	sunflower	T5	16	KAN	30.1 ± 1.8	10.1	1.00	1.00
4–12 (pooled)	both	T1–T3	—	PLS	—	—	0.86	0.98
4–12 (pooled)	both	T1–T3	—	SVR	—	—	0.66	1.00
4–12 (pooled)	both	T1–T3	—	RF	—	—	0.45	0.85
4–12 (pooled)	both	T1–T3	—	MLP	—	—	0.96	0.97
4–12 (pooled)	both	T1–T3	—	CNN	—	—	0.62	0.55
4–12 (pooled)	both	T1–T3	—	KAN	—	—	0.80	0.68
4–100 (pooled)	both	T1–T5	—	PLS	—	—	0.98	—
4–100 (pooled)	both	T1–T5	—	SVR	—	—	0.94	—
4–100 (pooled)	both	T1–T5	—	RF	—	—	0.89	—
4–100 (pooled)	both	T1–T5	—	MLP	—	—	0.98	—
4–100 (pooled)	both	T1–T5	—	CNN	—	—	0.93	—
4–100 (pooled)	both	T1–T5	—	KAN	—	—	0.97	—

Table S19. Predictions on authentic tahini by production lot. Threshold (% tahini): predicted tahini fraction below which a spectrum is flagged, re-chosen without the held-out lot. Spectra flagged: fraction of the lot's authentic spectra below the threshold. Sample mean flagged: whether the replicate-mean prediction falls below the threshold. Rows 'all': pooled bias, fraction of spectra flagged, and number of authentic samples flagged out of five. Last column: mean predicted adulterant under the three grouped five-fold partitions of Table 4, averaged over partitions. Nested and frozen leave-one-lot-out (LOLO) as in Section S4. SD is over replicate spectra.

Model	Lot	Threshold (% tahini)	Nested LOLO: predicted adulterant, mean ± SD (%)	Nested LOLO: spectra flagged	Nested LOLO: sample mean flagged	Frozen LOLO: predicted adulterant, mean ± SD (%)	Frozen LOLO: spectra flagged	Frozen LOLO: sample mean flagged	Table 4 partitions: predicted adulterant, mean (%)
PLS	T1	92.5	15.3 ± 2.3	1.00	yes	6.5 ± 2.0	0.30	no	3.7
PLS	T2	89.0	4.3 ± 2.0	0.00	no	5.7 ± 2.0	0.01	no	0.4
PLS	T3	91.5	2.4 ± 1.6	0.00	no	1.2 ± 1.3	0.00	no	2.5
PLS	T4	92.5	2.3 ± 0.8	0.00	no	2.3 ± 0.9	0.00	no	2.3
PLS	T5	93.0	10.3 ± 1.8	0.97	yes	10.4 ± 1.8	0.96	yes	6.8

https://doi.org/10.3390/foods15203639

Model	Lot	Threshold (% tahini)	Nested LOLO: predicted adulterant, mean ± SD (%)	Nested LOLO: spectra flagged	Nested LOLO: sample mean flagged	Frozen LOLO: predicted adulterant, mean ± SD (%)	Frozen LOLO: spectra flagged	Frozen LOLO: sample mean flagged	Table 4 partitions: predicted adulterant, mean (%)
PLS	all	—	6.9	0.39	2/5	5.2	0.25	1/5	3.1
SVR	T1	92.5	32.5 ± 2.7	1.00	yes	33.1 ± 3.0	1.00	yes	10.2
SVR	T2	89.0	12.9 ± 5.5	0.56	yes	12.4 ± 5.4	0.54	yes	10.5
SVR	T3	91.5	5.9 ± 4.7	0.30	no	5.7 ± 4.9	0.32	no	8.9
SVR	T4	92.5	14.0 ± 5.4	0.87	yes	16.0 ± 6.3	0.91	yes	11.6
SVR	T5	93.0	12.6 ± 4.7	0.89	yes	13.5 ± 5.2	0.92	yes	15.5
SVR	all	—	15.6	0.72	4/5	16.1	0.74	4/5	11.3
RF	T1	92.5	19.9 ± 4.5	1.00	yes	19.9 ± 4.6	1.00	yes	8.5
RF	T2	89.0	1.2 ± 3.0	0.04	no	1.1 ± 2.9	0.04	no	4.2
RF	T3	91.5	1.1 ± 1.5	0.00	no	1.1 ± 1.5	0.00	no	3.3
RF	T4	92.5	8.5 ± 7.3	0.46	yes	8.3 ± 7.4	0.46	yes	2.6
RF	T5	93.0	6.1 ± 5.6	0.36	no	6.0 ± 5.5	0.36	no	4.8
RF	all	—	7.4	0.37	2/5	7.3	0.37	2/5	4.7
MLP	T1	92.5	11.7 ± 1.8	0.99	yes	12.3 ± 1.8	1.00	yes	4.2
MLP	T2	89.0	3.2 ± 2.1	0.00	no	3.2 ± 2.0	0.00	no	3.5
MLP	T3	91.5	0.7 ± 0.3	0.00	no	0.7 ± 0.3	0.00	no	0.4
MLP	T4	92.5	1.5 ± 0.5	0.00	no	3.9 ± 0.7	0.00	no	3.1
MLP	T5	93.0	10.3 ± 2.2	0.93	yes	10.9 ± 2.5	0.93	yes	8.2
MLP	all	—	5.5	0.38	2/5	6.2	0.39	2/5	3.9
CNN	T1	92.5	5.2 ± 3.7	0.20	no	2.0 ± 2.6	0.05	no	5.2
CNN	T2	89.0	12.7 ± 5.8	0.51	yes	13.6 ± 6.2	0.59	yes	6.3
CNN	T3	91.5	0.9 ± 0.7	0.00	no	0.9 ± 0.7	0.00	no	1.4
CNN	T4	92.5	0.9 ± 1.1	0.00	no	1.4 ± 2.0	0.03	no	1.2
CNN	T5	93.0	5.2 ± 5.1	0.23	no	5.8 ± 5.8	0.25	no	6.3
CNN	all	—	5.0	0.19	1/5	4.8	0.18	1/5	4.1
KAN	T1	92.5	4.6 ± 1.9	0.10	no	13.6 ± 2.5	0.99	yes	6.6
KAN	T2	89.0	3.5 ± 1.4	0.00	no	3.5 ± 1.4	0.00	no	1.6
KAN	T3	91.5	1.1 ± 0.9	0.01	no	1.9 ± 1.2	0.00	no	0.6
KAN	T4	92.5	4.0 ± 0.8	0.00	no	4.6 ± 0.8	0.00	no	3.5
KAN	T5	93.0	8.6 ± 2.3	0.72	yes	9.0 ± 3.8	0.73	yes	9.1
KAN	all	—	4.3	0.16	1/5	6.5	0.34	2/5	4.3

S17. Uncertainty of the Detection Limit and R^2 with Production Lots Resampled

A bootstrap that resamples spectra or physical samples treats all 55 samples as independent, although samples of the same production lot share the same authentic tahini base. We therefore used a two-stage bootstrap: in each of 2000 draws, the five production lots were resampled with replacement, and then the physical samples within each drawn lot were resampled with replacement, keeping all replicate spectra of a drawn sample. On each draw we recomputed the tahini-fraction R^2 and the out-of-fold detection limit, LOD = $3.3\sigma/S$, where σ is the standard deviation of the predicted adulterant fraction on authentic spectra and S is the slope of the regression of predicted on true adulterant fraction. Draws without enough authentic spectra to estimate σ (10 of 2000) were excluded from the LOD analysis. Models were compared on the same draws using the paired difference, its 2.5–97.5 percentile interval, a two-sided bootstrap p-value (twice the smaller tail fraction) and Holm's correction over the 15 model pairs, applied separately for each metric and design. The analysis was applied to two sets of out-of-fold predictions: the three grouped five-fold partitions of Table 4, with each spectrum's prediction averaged over the

partitions (script revision/r05_oof_lod.py), and the nested and frozen leave-one-lot-out designs of Section S4 (script revision/r03_low_level_authentic_bootstrap.py).

Table 4 partitions (Table S20). The median LOD was 7.40% [95% interval 3.30, 11.45] for PLS, 7.24% [2.47, 14.61] for the MLP, 11.68% [2.35, 19.60] for the KAN, 12.27% [7.27, 16.33] for RF, 13.41% [2.67, 21.61] for the CNN and 20.60% [15.51, 26.62] for SVR. After Holm correction, the LOD differed significantly only between SVR and each of PLS, the MLP, the CNN and the KAN ($p < 0.001$; SVR against RF, $p = 0.254$). The PLS–KAN difference was −4.24 percentage points [−10.83, 3.14] ($p = 1.000$). For R^2, PLS was higher than the KAN (+0.017 [0.005, 0.034], $p < 0.001$), the CNN, RF and SVR; the MLP was higher than the CNN ($p < 0.001$), RF ($p = 0.024$) and SVR ($p < 0.001$); and the KAN was higher than SVR ($p = 0.018$). The MLP–KAN ($p = 0.147$) and PLS–MLP ($p = 0.399$) differences were not significant.

Leave-one-lot-out. In the nested design, the median R^2 was 0.977 [0.956, 0.988] for the KAN, 0.963 [0.915, 0.990] for the MLP and 0.960 [0.880, 0.989] for PLS, and the median LOD was 8.19% [2.98, 13.89], 15.30% [1.64, 19.88] and 14.59% [3.41, 24.83], respectively. The corresponding LOD point estimates, computed from all out-of-fold predictions without resampling, were 10.31% for the KAN, 17.52% for the MLP and 19.48% for PLS (20.71% for the CNN, 32.83% for RF and 45.34% for SVR). These are the values reported in the article; the bootstrap medians are lower because the LOD is a ratio with a skewed sampling distribution. There is no single agreed definition of the detection limit for multivariate calibration: an IUPAC-consistent PLS limit has been proposed as an interval that depends on the variation of the background composition in the calibration set [65], so the single-value limits reported here are screening-level indications. The KAN did not differ significantly from PLS, the MLP or the CNN in either metric ($p = 1.000$) but differed from RF and SVR ($p < 0.001$). In the frozen design, the ranking of the KAN and PLS point estimates was reversed (R^2 0.957 against 0.976; LOD 14.84% against 9.93%), again without a significant difference ($p = 1.000$).

Interpretation and limitations. Once lot-to-lot variation is included, the detection-limit intervals of all models except SVR overlap widely. Under the Table 4 partitions, the only statistically significant pairwise LOD differences involve SVR; in the leave-one-lot-out designs, RF also differed from the KAN (nested $p < 0.001$, frozen $p = 0.012$) and, in the nested design, from PLS ($p = 0.033$). The absence of significant differences does not show that the models are equivalent; no equivalence test was performed, and the intervals are wide enough to contain differences of practical size (for example, up to 10.8 percentage points between PLS and the KAN). With only five lots, the first stage of the bootstrap can draw at most five distinct lots, so percentile intervals are approximate and probably too narrow. The bootstrap medians differ from the point estimates of Table 4 (for example, 7.40% against 8.93% for PLS) partly because the LOD is a ratio with a skewed sampling distribution; Table 4 reports the point estimates.

Table S20. Two-stage (lot, then sample) bootstrap of the tahini-fraction R^2 and the out-of-fold detection limit (LOD), 2000 draws (1990 for LOD). Model rows: bootstrap median [2.5, 97.5 percentile]. Difference rows: paired difference (first model minus second) on the same draws, median [2.5, 97.5 percentile]; p: two-sided bootstrap p-value, Holm-adjusted over the 15 pairs within each column. A negative LOD difference favours the first model. Table 4 partitions: predictions averaged over the three grouped five-fold partitions of Table 4. Nested and frozen LOLO as in Section S4.

Model or paired difference	R^2, Table 4 partitions	LOD (%), Table 4 partitions	R^2, nested LOLO	LOD (%), nested LOLO	R^2, frozen LOLO	LOD (%), frozen LOLO
PLS	0.990 [0.978, 0.996]	7.40 [3.30, 11.45]	0.960 [0.880, 0.989]	14.59 [3.41, 24.83]	0.976 [0.947, 0.991]	9.93 [3.69, 16.29]
SVR	0.920 [0.856, 0.957]	20.60 [15.51, 26.62]	0.841 [0.489, 0.939]	26.85 [17.92, 65.20]	0.830 [0.470, 0.938]	31.58 [19.08, 66.74]

Model or paired difference	R^2, Table 4 partitions	LOD (%), Table 4 partitions	R^2, nested LOLO	LOD (%), nested LOLO	R^2, frozen LOLO	LOD (%), frozen LOLO
RF	0.939 [0.894, 0.965]	12.27 [7.27, 16.33]	0.883 [0.715, 0.956]	27.96 [7.28, 41.67]	0.883 [0.715, 0.956]	28.07 [7.15, 41.79]
MLP	0.986 [0.967, 0.993]	7.24 [2.47, 14.61]	0.963 [0.915, 0.990]	15.30 [1.64, 19.88]	0.960 [0.906, 0.989]	15.28 [2.54, 20.75]
CNN	0.965 [0.937, 0.984]	13.41 [2.67, 21.61]	0.950 [0.881, 0.981]	17.76 [2.74, 26.82]	0.945 [0.869, 0.977]	19.75 [4.07, 29.53]
KAN	0.974 [0.944, 0.989]	11.68 [2.35, 19.60]	0.977 [0.956, 0.988]	8.19 [2.98, 13.89]	0.957 [0.891, 0.985]	14.84 [3.68, 21.98]
PLS − SVR	+0.070 [0.036, 0.126]; p < 0.001	−13.31 [−17.74, −8.18]; p < 0.001	+0.123 [0.039, 0.387]; p < 0.001	−21.73 [−42.76, −6.96]; p < 0.001	+0.147 [0.043, 0.497]; p < 0.001	−26.34 [−55.91, −8.60]; p < 0.001
PLS − RF	+0.050 [0.022, 0.095]; p < 0.001	−4.48 [−11.07, 2.95]; p = 1.000	+0.078 [0.017, 0.178]; p = 0.044	−13.68 [−24.82, −0.63]; p = 0.033	+0.091 [0.013, 0.253]; p = 0.077	−18.67 [−33.15, 0.92]; p = 1.000
PLS − MLP	+0.005 [−0.001, 0.012]; p = 0.399	−0.52 [−5.32, 3.89]; p = 1.000	−0.004 [−0.039, 0.008]; p = 1.000	+1.57 [−2.86, 5.65]; p = 1.000	+0.014 [−0.004, 0.055]; p = 1.000	−2.86 [−10.41, 3.31]; p = 1.000
PLS − CNN	+0.024 [0.009, 0.049]; p < 0.001	−6.12 [−12.63, 2.09]; p = 1.000	+0.007 [−0.065, 0.079]; p = 1.000	−1.28 [−19.89, 12.88]; p = 1.000	+0.030 [−0.001, 0.100]; p = 0.657	−8.01 [−21.35, 3.66]; p = 1.000
PLS − KAN	+0.017 [0.005, 0.034]; p < 0.001	−4.24 [−10.83, 3.14]; p = 1.000	−0.013 [−0.089, 0.005]; p = 1.000	+3.21 [−1.73, 18.42]; p = 1.000	+0.017 [−0.005, 0.073]; p = 1.000	−3.17 [−12.34, 3.86]; p = 1.000
SVR − RF	−0.021 [−0.089, 0.046]; p = 1.000	+8.51 [0.35, 15.75]; p = 0.254	−0.052 [−0.250, 0.049]; p = 1.000	+10.26 [−4.94, 24.79]; p = 1.000	−0.062 [−0.265, 0.045]; p = 1.000	+11.24 [−1.19, 26.02]; p = 1.000
SVR − MLP	−0.066 [−0.116, −0.033]; p < 0.001	+12.22 [10.91, 16.88]; p < 0.001	−0.127 [−0.427, −0.034]; p < 0.001	+23.20 [4.79, 47.27]; p < 0.001	−0.134 [−0.439, −0.034]; p < 0.001	+25.33 [5.58, 47.45]; p < 0.001
SVR − CNN	−0.047 [−0.098, −0.004]; p = 0.234	+6.45 [2.37, 18.59]; p < 0.001	−0.109 [−0.447, −0.001]; p = 0.432	+19.45 [−3.28, 52.11]; p = 1.000	−0.112 [−0.466, 0.014]; p = 0.928	+19.08 [−4.92, 58.30]; p = 1.000
SVR − KAN	−0.053 [−0.101, −0.019]; p = 0.018	+7.95 [5.13, 18.31]; p < 0.001	−0.135 [−0.480, −0.040]; p < 0.001	+21.62 [8.26, 57.20]; p < 0.001	−0.131 [−0.425, −0.036]; p < 0.001	+25.24 [7.11, 44.97]; p < 0.001
RF − MLP	−0.046 [−0.093, −0.012]; p = 0.024	+4.28 [−4.00, 13.00]; p = 1.000	−0.084 [−0.211, −0.012]; p = 0.080	+15.81 [−0.12, 27.38]; p = 0.543	−0.079 [−0.205, −0.007]; p = 0.220	+16.00 [−1.84, 26.84]; p = 1.000
RF − CNN	−0.027 [−0.060, 0.002]; p = 0.370	−1.59 [−11.21, 10.22]; p = 1.000	−0.068 [−0.222, 0.045]; p = 1.000	+11.41 [−13.44, 28.11]; p = 1.000	−0.061 [−0.222, 0.056]; p = 1.000	+9.62 [−15.58, 32.70]; p = 1.000
RF − KAN	−0.035 [−0.079, 0.004]; p = 0.370	+1.18 [−9.46, 8.90]; p = 1.000	−0.092 [−0.252, −0.020]; p < 0.001	+20.54 [2.56, 34.48]; p < 0.001	−0.075 [−0.185, −0.014]; p = 0.024	+15.70 [1.38, 24.95]; p = 0.012
MLP − CNN	+0.018 [0.005, 0.043]; p < 0.001	−5.73 [−10.43, 1.79]; p = 0.884	+0.011 [−0.033, 0.083]; p = 1.000	−3.96 [−20.10, 8.08]; p = 1.000	+0.013 [−0.039, 0.093]; p = 1.000	−5.31 [−22.66, 13.14]; p = 1.000
MLP − KAN	+0.012 [0.001, 0.024]; p = 0.147	−4.06 [−7.78, 2.82]; p = 1.000	−0.011 [−0.051, 0.005]; p = 1.000	+4.39 [−3.68, 12.93]; p = 1.000	+0.004 [−0.010, 0.022]; p = 1.000	−0.39 [−6.19, 3.22]; p = 1.000
CNN − KAN	−0.007 [−0.033, 0.010]; p = 1.000	+1.16 [−3.13, 10.68]; p = 1.000	−0.025 [−0.095, 0.005]; p = 1.000	+7.89 [−2.46, 21.83]; p = 1.000	−0.009 [−0.093, 0.054]; p = 1.000	+4.14 [−14.48, 24.02]; p = 1.000

S18. Mango Prediction Errors by Ripening Treatment, Cultivar, Temperature Label and Region

The mango dataset was assembled by its authors to include, rather than to control, variation in harvest season, growing region, cultivar, ripening treatment (hard green or ripened) and a temperature treatment label (Low, Mid, High or No). For each model, we measured how much of the external-test error each factor explains, using the season-4 test predictions from the reproduction of the mango benchmark with the released code (Section S8; script revision/r08_mango_covariates.py). For each factor we report the share of each level in calibration (seasons 1–3) and test (season 4) spectra, and, per model, the bias (mean of predicted minus reference dry matter, DM) and the root-mean-square error (RMSE) within each level.

The factors could not be analysed jointly. In the season-4 test set the temperature label has a single level (No), and cultivar is nested within ripening treatment, so a model containing all factors is not identifiable. Each factor was therefore tested separately in a one-way linear model of the residual on that factor; partial η^2 was obtained from the type-II analysis of variance, and the p-value from a Wald test with covariance clustered by fruit population (18 test populations), because spectra of the same population are not independent. The temperature label was not testable.

Results (Table S21). The main distribution shift between the calibration and test sets was in ripening treatment: 89.3% of calibration spectra but 38.7% of test spectra came from hard-green fruit, and 61.3% of test spectra from ripened fruit. Every test level was represented in calibration; six cultivars (1201, 1243, 4069, Keitt, LadyG and LadyJ) and three temperature labels (Low, Mid and High) occurred only in calibration. Cultivar was associated with the residual for PLS (partial $\eta^2 = 0.200$, clustered $p < 0.001$) and the KAN (0.103, $p = 0.010$), but not significantly for the MLP (0.106, $p = 0.099$), the CNN (0.103, $p = 0.110$), RF (0.136, $p = 0.206$) or SVR (0.047, $p = 0.342$). The two cultivars with the highest mean reference dry matter, KP (19.68%) and HG (18.45%), were underpredicted by all six models (KAN bias −0.46 and −0.36% DM, against +0.33 for Caly and −0.13 for R2E2). Ripening treatment was nominally associated with the residual for the MLP ($p = 0.034$) and region with the residual for SVR ($p = 0.039$), but neither association remained significant after Bonferroni correction; all other ripening-treatment and region tests gave $p > 0.05$. Ripened fruit had a negative bias for all six models (−0.22 to −0.44% DM). These 18 tests were not adjusted for multiplicity; with a Bonferroni correction, only the PLS–cultivar association would remain significant at the 0.05 level.

Interpretation and limitations. Part of the season-4 error varies systematically with cultivar, and the high-dry-matter cultivars are underpredicted by every model. Cultivar and reference dry matter are confounded in this test set, so we cannot separate a cultivar effect from the regression-to-the-mean behaviour that shrinks predictions at the extremes of the calibration range. The clustered tests are based on 3–11 populations per level and are therefore approximate; a non-significant result does not show that a factor has no effect. The temperature label could not be assessed, and fruit size and other properties not recorded in the public dataset could not be examined.

Table S21. Season-4 prediction residuals of the mango dry-matter models by factor level. Shares are percentages of calibration (seasons 1–3) and test (season 4) spectra. Bias: mean of predicted minus reference dry matter (% DM); RMSE in parentheses. One-way test rows: partial η^2 (η^2p) and the Wald p-value clustered by fruit population (18 test populations), one factor at a time; not adjusted for multiplicity. Predictions are those of the reproduction of the mango benchmark with the released code (Section S8), in which the MLP is the (256, 128) configuration on 24 PLS scores; the other models use their published configurations. Test spectra (populations): number of season-4 spectra and fruit populations at the level. Reference DM: mean reference dry matter of those test spectra. —, level absent from the test set; the temperature label was not testable (single test level).

Factor	Level	Calibration share (%)	Test share (%)	Test spectra (populations)	Reference DM, mean (%)	PLS: bias (RMSE), % DM	SVR: bias (RMSE), % DM	RF: bias (RMSE), % DM	MLP: bias (RMSE), % DM	CNN: bias (RMSE), % DM	KAN: bias (RMSE), % DM
Ripening treatment	Hard Green	89.3	38.7	560 (7)	16.07	+0.20 (0.95)	+0.12 (0.97)	+0.08 (1.13)	+0.24 (0.94)	−0.29 (1.13)	+0.09 (0.87)
Ripening treatment	Ripen	10.7	61.3	888 (11)	17.61	−0.34 (1.11)	−0.42 (1.10)	−0.44 (1.27)	−0.37 (0.97)	−0.40 (0.98)	−0.22 (1.06)

Factor	Level	Calibration share (%)	Test share (%)	Test spectra (populations)	Reference DM, mean (%)	PLS: bias (RMSE), % DM	SVR: bias (RMSE), % DM	RF: bias (RMSE), % DM	MLP: bias (RMSE), % DM	CNN: bias (RMSE), % DM	KAN: bias (RMSE), % DM
Ripening treatment	one-way test	—	—	—	—	η^2p = 0.062; p = 0.116	η^2p = 0.063; p = 0.058	η^2p = 0.045; p = 0.181	η^2p = 0.096; p = 0.034	η^2p = 0.003; p = 0.736	η^2p = 0.024; p = 0.283
Cultivar	1201	3.9	0.0	0	—	—	—	—	—	—	—
Cultivar	1243	3.9	0.0	0	—	—	—	—	—	—	—
Cultivar	4069	3.9	0.0	0	—	—	—	—	—	—	—
Cultivar	Caly	29.3	32.0	463 (6)	16.02	+0.52 (0.81)	+0.07 (0.72)	+0.14 (0.80)	+0.28 (0.74)	−0.08 (0.74)	+0.33 (0.74)
Cultivar	HG	14.4	16.5	239 (3)	18.45	−0.71 (1.46)	−0.31 (1.27)	−0.93 (1.61)	−0.54 (1.24)	−0.54 (1.29)	−0.36 (1.11)
Cultivar	KP	22.3	22.0	318 (4)	19.68	−0.33 (1.24)	−0.53 (1.46)	−0.66 (1.68)	−0.39 (1.27)	−0.87 (1.45)	−0.46 (1.39)
Cultivar	Keitt	3.2	0.0	0	—	—	—	—	—	—	—
Cultivar	LadyG	3.4	0.0	0	—	—	—	—	—	—	—
Cultivar	LadyJ	3.9	0.0	0	—	—	—	—	—	—	—
Cultivar	R2E2	11.8	29.6	428 (5)	15.32	−0.36 (0.82)	−0.22 (0.85)	+0.07 (0.86)	−0.15 (0.66)	−0.17 (0.77)	−0.13 (0.77)
Cultivar	one-way test	—	—	—	—	η^2p = 0.200; p < 0.001	η^2p = 0.047; p = 0.342	η^2p = 0.136; p = 0.206	η^2p = 0.106; p = 0.099	η^2p = 0.103; p = 0.110	η^2p = 0.103; p = 0.010
Temperature label	High	13.8	0.0	0	—	—	—	—	—	—	—
Temperature label	Low	14.4	0.0	0	—	—	—	—	—	—	—
Temperature label	Mid	14.4	0.0	0	—	—	—	—	—	—	—
Temperature label	No	57.3	100.0	1448 (18)	17.02	−0.13 (1.05)	−0.21 (1.05)	−0.24 (1.21)	−0.13 (0.96)	−0.36 (1.04)	−0.10 (0.99)
Temperature label	one-way test	—	—	—	—	not testable	not testable	not testable	not testable	not testable	not testable
Region	NT	72.6	48.5	702 (9)	17.76	−0.32 (1.02)	−0.50 (1.15)	−0.48 (1.23)	−0.37 (0.95)	−0.55 (1.16)	−0.32 (1.06)
Region	QLD	27.4	51.5	746 (9)	16.31	+0.05 (1.07)	+0.06 (0.96)	0.00 (1.20)	+0.10 (0.96)	−0.17 (0.91)	+0.11 (0.91)
Region	one-way test	—	—	—	—	η^2p = 0.032; p = 0.295	η^2p = 0.075; p = 0.039	η^2p = 0.040; p = 0.222	η^2p = 0.062; p = 0.107	η^2p = 0.037; p = 0.203	η^2p = 0.047; p = 0.144

S19. Input Representation for the Non-Linear Mango Models

The non-linear score models (the KAN, the MLP and SVR) read PLS latent scores, a supervised but linear compression of the spectrum. On a matrix in which the response is non-linear, a linear compression could discard information that a non-linear model would use. We therefore changed only the input representation and kept all other elements of the published mango benchmark fixed: the external test set (season 4), the preprocessing

(first-derivative Savitzky–Golay filter), the model configurations and the number of components (script revision/r11_mango_input_representation.py). Four representations were compared: the published PLS scores; principal component analysis (PCA) scores, an unsupervised linear compression that isolates the effect of supervision; the full preprocessed spectrum of 103 channels, with no compression; and kernel PCA scores with a radial basis function (RBF) kernel, an unsupervised non-linear compression fitted on a fixed random subset of 3000 training spectra with the kernel width set by the median heuristic before the test set was seen. Stochastic models were fitted with three seeds; SVR is deterministic.

Results (Table S22). The non-linear compression gave the lowest R^2 for the KAN and the MLP, and the second-lowest for SVR: 0.719 for the KAN, 0.687 for the MLP and 0.754 for SVR, against 0.832–0.876 for every other representation except SVR on the full spectrum (0.665). The two linear compressions gave similar results (KAN 0.861 with PLS and 0.853 with PCA; MLP 0.868 and 0.876; SVR 0.844 and 0.858). The full spectrum did not improve on the linear compressions: the KAN reached 0.832, the MLP 0.866 and SVR 0.665.

Interpretation and limitations. On these data, compressing the spectrum linearly before the non-linear model did not reduce accuracy relative to giving the model the full spectrum or a non-linear compression; for the KAN, the published PLS scores gave the highest mean R^2 of the four representations. If the relation is non-linear, a plausible explanation is that the non-linearity of the mango calibration lies not in the spectral directions themselves but in the relation between a small number of these directions and dry matter, a relation that the non-linear model captures after the compression. The comparison covers one kernel with one width selection rule and one subset size, so it does not show that no non-linear compression could perform better. The differences between PLS and PCA scores were not tested.

Table S22. Mango external test (season 4) for the three non-linear score models with four input representations. Preprocessing, model configurations and the number of components are those of the published benchmark (Table 3 of the article); only the representation changes. Input dimension: number of scores or channels passed to the model. R^2: mean and SD over three seeds (SVR deterministic, one run; SD not defined). RMSEP: root-mean-square error of prediction on the season-4 test set (% DM). Seeds: number of model seeds. DM, dry matter.

Model	Input representation	Input dimension	R^2 (mean)	R^2 (SD)	RMSEP (% DM)	Seeds
KAN	PLS scores (published)	24	0.861	0.013	0.995	3
KAN	PCA scores	24	0.853	0.024	1.020	3
KAN	Full spectrum	103	0.832	0.011	1.094	3
KAN	Kernel PCA scores (RBF)	24	0.719	0.033	1.414	3
MLP	PLS scores (published)	16	0.868	0.008	0.968	3
MLP	PCA scores	16	0.876	0.006	0.940	3
MLP	Full spectrum	103	0.866	0.007	0.976	3
MLP	Kernel PCA scores (RBF)	16	0.687	0.054	1.490	3
SVR	PLS scores (published)	16	0.844	—	1.054	1
SVR	PCA scores	16	0.858	—	1.006	1
SVR	Full spectrum	103	0.665	—	1.545	1
SVR	Kernel PCA scores (RBF)	16	0.754	—	1.323	1

S20. Learning Curve over the Number of Training Samples

The primary tahini training partition contains 38 physical samples. To examine whether the flexible models are limited by this number, we trained every model on subsets of these samples and evaluated each on the unchanged 17-sample test partition (script revision/r10_learning_curve.py). For each of five draws, the authentic and adulterated training samples were randomly permuted separately, and subsets of n = 10, 15, 20, 25, 30 and 38 samples were taken from the start of these permutations, preserving the proportion of authentic samples in the training partition. The subsets are therefore nested within a draw. Each model was fitted at its published configuration, with the preprocessing and the PLS projection refitted on each subset and the model seed set to 42 plus the draw index. At n = 38 every draw uses the full partition, so the spread reflects only the model seed and is zero for the deterministic PLS and SVR.

Results (Table S23; Figure S9). PLS reached R^2 = 0.990 ± 0.004 with 10 samples and 0.994 with 38, and was the most accurate model at every n. The MLP's R^2 rose from 0.917 ± 0.042 at n = 10 to 0.972 ± 0.012 at n = 15 and 0.987 ± 0.001 at n = 38, and that of the CNN from 0.888 ± 0.023 to 0.969 ± 0.003 over the same range. The KAN was the most variable model at small n (0.813 ± 0.232 at n = 10, minimum 0.399; 0.849 ± 0.153 at n = 15) and its R^2 rose to 0.928 ± 0.050 at n = 20, 0.964 ± 0.025 at n = 30 and 0.982 ± 0.010 at n = 38. Between 30 and 38 samples its mean R^2 increased by 0.017, against 0.002 for the MLP, 0.004 for the CNN and less than 0.001 for PLS. RF and SVR remained below 0.93 at every n.

Interpretation and limitations. With the published configurations, the MLP and CNN curves had largely flattened by 25–30 samples, whereas the KAN curve was still rising at 38 samples and its between-draw spread was large below 20 samples. Judged by these point estimates, the KAN is the model whose accuracy changes most with the number of training samples in this range, and more samples may improve its accuracy, although the curve does not show by how much. PLS needed the fewest samples. The differences between models were not tested, the points on each curve are not independent (the subsets are nested), and all points were scored on a single 17-sample test partition.

Table S23. External-test R^2 (tahini fraction) against the number of physical samples in training. Nested random subsets of the 38-sample primary training partition, five draws per size, published configurations; test set: the 17-sample primary test partition. Mean ± SD over draws, minimum in parentheses. At n = 38 all draws use the full partition.

Training samples	PLS: R^2 mean ± SD (min)	SVR: R^2 mean ± SD (min)	RF: R^2 mean ± SD (min)	MLP: R^2 mean ± SD (min)	CNN: R^2 mean ± SD (min)	KAN: R^2 mean ± SD (min)
10	0.990 ± 0.004 (0.983)	0.500 ± 0.103 (0.346)	0.773 ± 0.039 (0.716)	0.917 ± 0.042 (0.861)	0.888 ± 0.023 (0.854)	0.813 ± 0.232 (0.399)
15	0.989 ± 0.004 (0.983)	0.793 ± 0.056 (0.708)	0.835 ± 0.045 (0.769)	0.972 ± 0.012 (0.958)	0.933 ± 0.010 (0.922)	0.849 ± 0.153 (0.590)
20	0.992 ± 0.002 (0.990)	0.815 ± 0.061 (0.748)	0.886 ± 0.035 (0.836)	0.975 ± 0.009 (0.961)	0.951 ± 0.007 (0.945)	0.928 ± 0.050 (0.850)
25	0.994 ± 0.001 (0.992)	0.866 ± 0.049 (0.793)	0.889 ± 0.031 (0.847)	0.984 ± 0.002 (0.982)	0.958 ± 0.007 (0.947)	0.943 ± 0.026 (0.919)
30	0.994 ± 0.001 (0.993)	0.861 ± 0.045 (0.795)	0.894 ± 0.033 (0.849)	0.985 ± 0.003 (0.982)	0.965 ± 0.002 (0.961)	0.964 ± 0.025 (0.921)
38	0.994 ± 0.000 (0.994)	0.925 ± 0.000 (0.925)	0.923 ± 0.002 (0.919)	0.987 ± 0.001 (0.985)	0.969 ± 0.003 (0.965)	0.982 ± 0.010 (0.966)

https://doi.org/10.3390/foods15203639

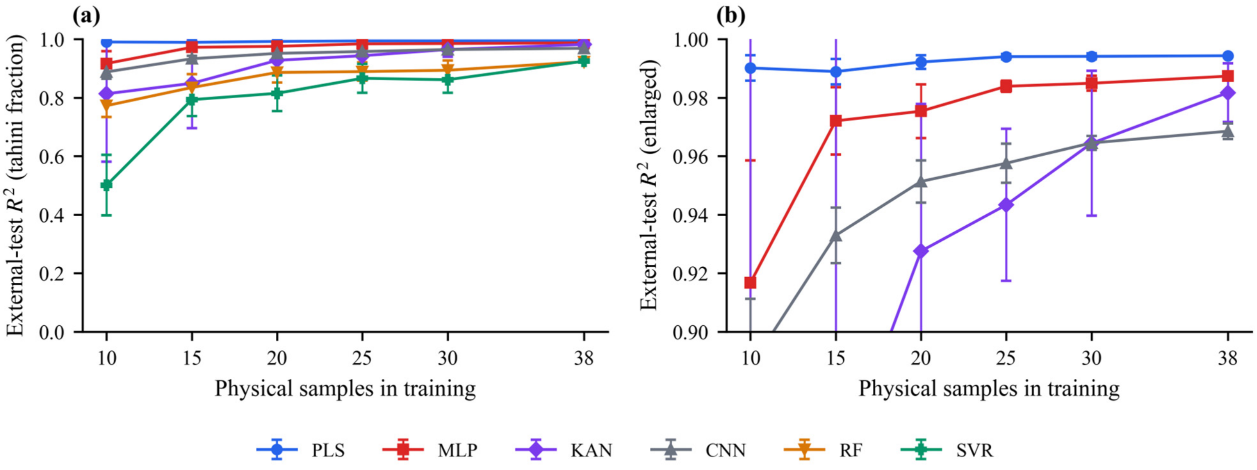

Figure S9. Learning curves for the tahini fraction. External-test R² against the number of physical training samples (10–38): (a) all six models; (b) the four leading models, enlarged above R² = 0.90 (points below 0.90 lie outside the plotted range). Points: mean over five draws of nested random subsets of the primary training partition; error bars: SD over draws. The test set (17 samples) is the same for every point. Data as in Table S23.

S21. Training Budget of the Parameter-Matched Comparison

Under the published fixed-step recipe of Section S7, the parameter-matched MLP received many more gradient updates than the KAN (3600 against 300 on tahini and 32,200 against 300 on mango), because its budget is set in epochs, each comprising one update per mini-batch of the training set, whereas the KAN's budget is a fixed number of 300 mini-batch steps. To test whether this difference, rather than the architecture, explains the comparison, each architecture was trained both at its own budget and at the other's, on the published external splits, at matched trainable parameters and with five seeds (script revision/r09_training_budget.py). The KAN kept its published configuration and was trained for 300 steps or for the MLP's number of updates; the MLP (a perceptron with one hidden layer sized to the KAN's trainable-parameter count and with the published batch-normalisation and dropout block) was trained for its published 200 epochs or for the number of epochs giving approximately 300 updates.

Results (Table S24). A larger budget did not help the KAN: with the MLP's number of updates its external R² fell from 0.980 to 0.962 on tahini, with a larger seed-to-seed SD (0.036 against 0.012), and from 0.860 to 0.854 on mango. A smaller budget cost the perceptron little: with approximately 300 updates it reached 0.986 on tahini and 0.883 on mango, against 0.992 and 0.894 at its own budget, and on both datasets it still scored higher than the KAN trained at the KAN's own budget.

Interpretation and limitations. According to the point estimates, the training budget does not explain the finding of Section 3.6 of the article that a parameter-matched MLP had a higher mean R² than the KAN (significantly so in five of the eight mango comparisons by a simple paired test, though in none after variance correction; Section S24): the perceptron at the KAN's budget still exceeded the KAN at the KAN's own budget (0.986 against 0.980 on tahini; 0.883 against 0.860 on mango), and the KAN did not gain from the perceptron's budget. The comparison uses the published optimiser settings of each architecture and one pair of budgets; the differences in Table S24 were not tested. This result supports the statement in Section S7 that the narrowing of the margin under early stopping reflects that procedure rather than the budget. Under early stopping, the margin narrowed to 0.003, but when the MLP's budget was reduced directly to that of the KAN, it

still scored higher (0.883 against 0.860); the narrowing under early stopping is therefore not evidence that the budget explains the comparison.

Table S24. Training-budget crossover at matched trainable parameters on the published external test sets. "Own" is each architecture's published budget; the other rows give each architecture the other's number of gradient updates. Gradient updates: optimiser steps actually taken (for the perceptron at the KAN's budget, the whole number of epochs closest to 300 updates, hence 306 and 322). Trainable parameters: number of trainable weights and biases. Mean, SD, minimum and maximum over five seeds (42, 7, 2024, 13 and 777). These seeds and the training code differ from those of Table S6, block 3, which is why the tahini KAN at its own budget scores 0.980 ± 0.012 here and 0.986 ± 0.006 there.

Dataset	Model	Budget	Gradient updates	Trainable parameters	R^2 (mean)	R^2 (SD)	Min	Max
tahini	KAN	Own	300	684	0.980	0.012	0.961	0.988
tahini	KAN	Perceptron's	3600	684	0.962	0.036	0.916	0.994
tahini	Parameter-matched MLP	Own	3600	689	0.992	0.001	0.990	0.994
tahini	Parameter-matched MLP	KAN's	306	689	0.986	0.006	0.977	0.991
mango	KAN	Own	300	8996	0.860	0.008	0.848	0.868
mango	KAN	Perceptron's	32,200	8996	0.854	0.006	0.849	0.863
mango	Parameter-matched MLP	Own	32,200	8989	0.894	0.005	0.889	0.899
mango	Parameter-matched MLP	KAN's	322	8989	0.883	0.005	0.875	0.888

S22. The KAN with the Favourable Single Factors Combined

In the single-factor sensitivity analysis (Table S4), eight variants scored above the published tahini KAN configuration in cross-validation. To check whether the model comparisons depend on a configuration that was known to be sub-optimal, we refitted the KAN with the favourable single factors combined: SNV followed by a first-derivative Savitzky–Golay filter (window 11), grid size 3, spline order 4 and hidden width 5, with the same 8 PLS scores as input (script revision/r13_kan_best_factors.py). The published KAN configuration (SNV, grid 5, order 3, width 3) and the published PLS and MLP configurations were run on the same splits. Two designs were used: the group-independent external test of Table 2 (five model seeds for the KANs and the MLP), and leave-one-lot-out validation with each configuration held fixed (three seeds per fold, averaged within the fold, then summarised over the five lots). The combination was not tuned further; the factors were chosen from the cross-validation of Table S4 on the primary training partition, so the external test set was used only once.

Results (Table S25). On the external test, the combined configuration gave $R^2 = 0.9919 \pm 0.0041$ (lowest seed 0.9848), against 0.9797 ± 0.0118 (lowest 0.9606) for the published configuration, 0.9873 ± 0.0017 for the MLP and 0.9943 for PLS. In leave-one-lot-out validation, it gave 0.9751 ± 0.0233 over lots (worst lot T1, 0.9374), against 0.9404 ± 0.0574 (worst 0.8414, T1) for the published configuration, 0.9545 ± 0.0454 (worst 0.8853) for the MLP and 0.9749 ± 0.0170 (worst 0.9522, T5) for PLS. The combined configuration had a higher R^2 than the published one in all five lots.

Interpretation and limitations. The published KAN configuration understated the KAN's accuracy: with the favourable factors combined, the KAN's external and leave-one-lot-out R^2 approached those of PLS. Its external R^2 remained below that of PLS (0.9919

against 0.9943), its leave-one-lot-out mean was nearly identical (0.9751 against 0.9749), and its worst-lot R^2 was lower (0.9374 against 0.9522). None of these differences was tested; they are rankings of point estimates over one external test set and five lots. The leave-one-lot-out result is not fully independent of the factor choice, because every lot contributed samples to the partition on which the factors were selected. Because the configuration was also held fixed rather than re-selected inside each fold, the leave-one-lot-out values are optimistic relative to a nested design, in which the published KAN reached 0.968 (worst 0.948) and PLS 0.950 (worst 0.837; Table S3).

Table S25. Tahini-fraction R^2 of the KAN with the favourable single factors of Table S4 combined, compared with the published KAN, MLP and PLS configurations. External test: the group-independent test set of Table 2, mean ± SD over five model seeds (range in parentheses; PLS is deterministic). LOLO: leave-one-lot-out with the configuration held fixed, three seeds averaged per lot, mean ± SD over the five lots; T1–T5: R^2 on each held-out lot. All KAN variants use 8 PLS scores as input. The single-seed value of Table 2 (0.987) is that of seed 42, one of the five seeds (42, 7, 2024, 13 and 777) summarised here. These seeds and the training code differ from those of Table S6, block 3, which is why the published configuration scores 0.980 ± 0.012 here and 0.986 ± 0.006 there.

Model and configuration	External test R^2, mean ± SD (range; seeds)	LOLO R^2, mean ± SD over lots	LOLO worst lot	T1	T2	T3	T4	T5
KAN, combined factors (SNV + SG first derivative, grid 3, order 4, width 5)	0.9919 ± 0.0041 (0.9848–0.9949; n = 5)	0.9751 ± 0.0233	0.9374	0.9374	0.9942	0.9882	0.9878	0.9678
KAN, published (SNV, grid 5, order 3, width 3)	0.9797 ± 0.0118 (0.9606–0.9880; n = 5)	0.9404 ± 0.0574	0.8414	0.8414	0.9770	0.9786	0.9644	0.9407
MLP, published	0.9873 ± 0.0017 (0.9854–0.9897; n = 5)	0.9545 ± 0.0454	0.8853	0.8853	0.9758	0.9944	0.9849	0.9321
PLS, published	0.9943 (deterministic)	0.9749 ± 0.0170	0.9522	0.9686	0.9702	0.9942	0.9892	0.9522

S23. Thirty Repeated Grouped Hold-Outs with Equivalence Tests

The corrected resampled t-test of Nadeau and Bengio was derived for J independent random training–test splits of one dataset, with the variance of the mean difference inflated by the factor ($1/J + n_{test}/n_{train}$). In Section S12, the test was applied to three seeds or five folds, a setting in which it has little power. Here, it is applied on tahini to the design for which it was derived: 30 random grouped hold-outs of 70% of the physical samples for training and 30% for testing (split seeds 1000–1029, none equal to that of the primary partition), with the complete model selection of the published benchmark (grouped three-fold inner cross-validation over the published search space) repeated inside each training set, so that no test sample informed any configuration (script revision/r04_repeated_grouped_splits.py). The preprocessing was that of the primary partition (SNV), and each model was fitted once per split. For each of the 15 pairs of models, the corrected t-test (with $\varrho = 0.470$, the ratio of test to training spectra averaged over splits) was applied to the 30 paired differences in the R^2 of the tahini fraction, with Holm adjustment over the

15 pairs. Because a non-significant difference does not show that two models are equivalent, corrected two one-sided tests of equivalence (TOST) were also applied at margins of ±0.01 and ±0.02 R^2, with Holm adjustment.

Results (Tables S26 and S27). PLS had the highest mean R^2 (0.990 ± 0.007), followed by the MLP (0.984 ± 0.009), the CNN (0.958 ± 0.022), the KAN (0.956 ± 0.048), RF (0.884 ± 0.059) and SVR (0.859 ± 0.153). The KAN's median (0.974) was higher than its mean, because a few splits gave markedly lower values (minimum 0.834); of the four leading models, it had the largest SD over splits. PLS had a higher R^2 than the KAN in all 30 splits (mean difference 0.034) and than the MLP in 25 of 30. After correction for the dependence between overlapping training sets and adjustment for multiplicity, no pair of models differed significantly (smallest adjusted $p = 0.26$), and no pair was shown to be equivalent at either margin (smallest adjusted TOST $p = 0.146$, for PLS and the MLP at ±0.02).

Interpretation and limitations. Increasing the number of splits from three to thirty did not make any comparison significant, because the corrected test accounts for the overlap between training sets, and this overlap does not shrink as J grows. With 55 physical samples, the design can neither resolve differences of a few hundredths of R^2 between the leading models nor establish their equivalence. The comparisons between models on tahini are therefore reported as rankings of point estimates. The consistent direction of the PLS–KAN difference (30 of 30 splits) is descriptive: the splits share samples, so a sign test on them is not valid. This pattern agrees with the conclusion of the article that on this near-linear matrix the KAN has no accuracy advantage over PLS, and with the larger split-to-split variability of the KAN at this sample size (Sections 3.6 and S20).

Table S26. Tahini-fraction R^2 over 30 random grouped 70/30 hold-outs with the model selection repeated inside each training set. Mean, SD, median, minimum and maximum over the 30 splits.

Model	R^2 (mean)	R^2 (SD)	Median	Minimum	Maximum
PLS	0.990	0.007	0.992	0.964	0.997
MLP	0.984	0.009	0.986	0.959	0.995
CNN	0.958	0.022	0.963	0.877	0.985
KAN	0.956	0.048	0.974	0.834	0.993
RF	0.884	0.059	0.901	0.732	0.955
SVR	0.859	0.153	0.902	0.146	0.964

Table S27. Pairwise comparisons over the 30 hold-outs of Table S26. Mean difference: mean over splits of $R^2(A) - R^2(B)$. Splits won by A: number of splits in which A had the higher R^2. p (corrected t): Nadeau–Bengio corrected resampled t-test, two-sided; p (Holm): adjusted over the 15 pairs. TOST: corrected two one-sided tests of equivalence at the stated margin, Holm-adjusted; a value below 0.05 would indicate equivalence within the margin.

Pair (A − B)	Mean difference in R^2	Splits won by A	p (corrected t)	p (Holm)	TOST ±0.01, p (Holm)	TOST ±0.02, p (Holm)
PLS − SVR	0.131	30/30	0.222	1.000	1.000	1.000
PLS − RF	0.106	30/30	0.017	0.262	1.000	1.000
PLS − MLP	0.005	25/30	0.377	1.000	1.000	0.146
PLS − CNN	0.031	30/30	0.021	0.281	1.000	1.000
PLS − KAN	0.034	30/30	0.306	1.000	1.000	1.000
SVR − RF	−0.025	14/30	0.833	1.000	1.000	1.000
SVR − MLP	−0.125	0/30	0.242	1.000	1.000	1.000
SVR − CNN	−0.099	2/30	0.337	1.000	1.000	1.000
SVR − KAN	−0.096	4/30	0.343	1.000	1.000	1.000
RF − MLP	−0.100	0/30	0.020	0.281	1.000	1.000
RF − CNN	−0.074	1/30	0.078	0.938	1.000	1.000
RF − KAN	−0.071	2/30	0.143	1.000	1.000	1.000
MLP − CNN	0.026	29/30	0.087	0.956	1.000	1.000

https://doi.org/10.3390/foods15203639

Pair (A − B)	Mean difference in R^2	Splits won by A	p (corrected t)	p (Holm)	TOST ±0.01, p (Holm)	TOST ±0.02, p (Holm)
MLP − KAN	0.029	26/30	0.359	1.000	1.000	1.000
CNN − KAN	0.003	9/30	0.930	1.000	1.000	1.000

S24. Paired Tests of the Parameter-Matched Comparison

Section 3.6 of the article compares the KAN with an MLP built to approximately the same number of trainable parameters in twelve configurations (Table S6, block 3): three capacities (tahini and the two mango capacities), two training rules (the fixed-step recipe and early stopping) and two KAN configurations (the published one and the one selected by its own inner search), each against the MLP trained by the same rule. Both architectures used the same five seeds (42, 7, 2024, 100 and 13), the same training partition and the same external test set, so the comparison is paired by seed (script paper1_rigor/r42_parameter_matched_tests.py). Table S28 gives, for each configuration, the mean difference with its 95% interval, a two-sided paired t-test with four degrees of freedom, the same test with the Nadeau–Bengio variance correction at $\varrho = n_{test}/n_{train}$ used for the seed-repeated families of Table S14, and two one-sided tests of equivalence at ±0.02 R^2 on the corrected variance, as in Section S23; each kind of test is Holm-adjusted over the twelve comparisons.

Results. The MLP had the higher mean R^2 in all twelve comparisons, and in all five seeds in eight of them; no comparison favoured the KAN. By the simple paired t-test, none of the four tahini differences was significant (adjusted $p \geq 0.41$), whereas on mango the MLP was significantly more accurate in five of the eight comparisons (adjusted $p \leq 0.029$): all four at the compact capacity, and the published configuration at the larger capacity under the fixed-step recipe. With the Nadeau–Bengio correction, the convention of Section 2.5 of the article for seed-repeated comparisons, no difference reached significance (smallest adjusted $p = 0.066$). On the same corrected variance, equivalence within ±0.02 R^2 was not shown for any pair (smallest adjusted $p = 0.227$); on the plain paired variance, one pair, the published tahini configuration under the fixed-step recipe, would have been classed as equivalent (adjusted $p = 0.042$).

Interpretation and limitations. Because all seeds share one partition, these tests show whether a difference exceeds seed-to-seed variation on this test set, not whether it would hold on new material; for the benchmark models that question is addressed by the thirty repeated grouped hold-outs of Section S23. The conclusion of Section 3.6 is therefore that the two architectures are comparable in accuracy, with the MLP ahead on every point estimate and significantly so in five mango comparisons by the simple test but in none after the Nadeau–Bengio correction; no KAN advantage was found.

Table S28. Paired tests of the parameter-matched comparison of Table S6, block 3: the MLP against the KAN at matched trainable parameters, paired over five seeds on one fixed external test set. Difference: mean over seeds of R^2(MLP) − R^2(KAN), with its uncorrected paired 95% t-interval (95% CI; neither variance-corrected nor adjusted for multiplicity). Seeds: number of seeds (of five) in which the MLP had the higher R^2. p (paired): two-sided paired t-test; p (corrected): the same test with the Nadeau–Bengio variance correction ($\varrho = n_{test}/n_{train}$); equivalence: two one-sided tests at ±0.02 R^2 on the corrected variance, as in Section S23, a value below 0.05 indicating equivalence within that margin. All p-values are Holm-adjusted over the twelve comparisons.

Dataset	Training rule	KAN configuration	MLP R^2	KAN R^2	Difference	95% CI	Seeds MLP higher	p (paired)	p (corrected)	Equivalence ±0.02, corrected (p)
tahini	fixed steps	published	0.9864	0.9859	+0.0005	−0.010 to +0.011	2/5	1.000	1.000	0.227
tahini	fixed steps	inner-CV	0.9864	0.9745	+0.0119	−0.002 to +0.025	5/5	0.418	1.000	1.000

Dataset	Training rule	KAN configuration	MLP R^2	KAN R^2	Difference	95% CI	Seeds MLP higher	p (paired)	p (corrected)	Equivalence ±0.02, corrected (p)
tahini	early stopping	published	0.9887	0.9653	+0.0234	−0.031 to +0.078	3/5	1.000	1.000	1.000
tahini	early stopping	inner-CV	0.9887	0.9690	+0.0197	−0.026 to +0.066	3/5	1.000	1.000	1.000
mango (accuracy)	fixed steps	published	0.8969	0.8615	+0.0354	+0.022 to +0.049	5/5	0.024	0.066	1.000
mango (accuracy)	fixed steps	inner-CV	0.8969	0.8730	+0.0239	+0.005 to +0.043	5/5	0.184	0.405	1.000
mango (accuracy)	early stopping	published	0.8759	0.8588	+0.0171	−0.006 to +0.040	5/5	0.558	1.000	1.000
mango (accuracy)	early stopping	inner-CV	0.8759	0.8729	+0.0030	−0.018 to +0.024	3/5	1.000	1.000	0.895
mango (compact)	fixed steps	published	0.8732	0.8266	+0.0465	+0.027 to +0.066	5/5	0.026	0.069	1.000
mango (compact)	fixed steps	inner-CV	0.8732	0.8117	+0.0615	+0.035 to +0.088	5/5	0.028	0.074	1.000
mango (compact)	early stopping	published	0.8752	0.8360	+0.0392	+0.022 to +0.057	5/5	0.028	0.074	1.000
mango (compact)	early stopping	inner-CV	0.8752	0.8311	+0.0441	+0.026 to +0.062	5/5	0.026	0.069	1.000

S25. Numerical Precision of the Exact Route, Input Clipping and the Constants of Equation (3)

The fidelity of an exact-route equation is reported as, for example, 0.9999995 rather than exactly 1. This is not numerical instability: in an elementary basis the printed expression and the network are the same function, and the departure from 1 arises only because the printed coefficients are rounded to three significant figures and terms smaller than 10^{-3} of the largest coefficient are omitted for readability, and because the network computes in single (32-bit) precision while the printed expression is evaluated in double precision. The network of Equation (3) cannot be regenerated bit for bit, because its initial weights were drawn before the seed was set (Section 2.8 of the article). Table S29 therefore separates the two sources of the departure on a refit of the same configuration, with the training seed unchanged and the initialisation also seeded (script paper1_rigor/r41_exact_route_precision.py; external R^2 0.9837, against 0.9841 for the published network). As printed, the refitted equation reaches a fidelity of $1 - 3.3 \times 10^{-6}$ (the published Equation (3): $1 - 4.7 \times 10^{-7}$); printed at full double precision with nothing omitted, it reaches $1 - 1.3 \times 10^{-14}$, the remaining difference (at most 5.8×10^{-7} in standardised units) being the single-precision round-off of the network. Five further refits that differed only in the initialisation seed gave an external R^2 of 0.9834–0.9846, as-printed fidelities from $1 - 1.5 \times 10^{-5}$ to $1 - 5.9 \times 10^{-7}$, 31–33 terms and all eight bands retained, so the initialisation changes the printed coefficients slightly but moves the external R^2 by less than 0.001 from that of the published network and leaves the bands of the equation unchanged.

Polynomial, Fourier and Gaussian basis functions are defined on [−1, 1], and a polynomial in particular can diverge outside that interval. Every input is therefore min–max scaled to [−1, 1] on the training partition, and any new value outside the training range is clipped to the nearest bound before either the network or the printed equation is evaluated; the edge functions are never evaluated outside the interval on which they were fitted. The rule is active in practice: 3.4% of the input values of the external test set (at least one band in 104 of its 408 spectra, 101 of them from the single authentic lot it contains, T4),

and 0.2–4.2% of those of each withheld production lot (eight bands re-selected and the scaling refitted without that lot), lay outside the training range and were clipped (Table S29). A clipped value indicates that the sample lies outside the calibration domain; its prediction is bounded but should be treated as an extrapolation, consistent with restricting the method to samples similar to the calibration population. That most of the clipped test spectra are authentic tahini from a single lot reflects the lot-to-lot variation of authentic tahini discussed in Section 3.2 of the article.

To evaluate Equation (3) from a recorded spectrum, the spectrum is SNV-corrected over all 1762 channels, the SNV-corrected absorbance a at each of the eight named wavenumbers is scaled as $2(a - \min)/(\max - \min) - 1$, with min and max its training range in Table S29, and clipped to [−1, 1]; these scaled values are the variables A of Equation (3), and the output $\hat{y}$ of the equation is converted to the tahini fraction as $76.16 + 32.73\,\hat{y}$ (%). This gives the tahini output alone, without the sum-to-100% rescaling that the network applies to its three outputs.

Table S29. Numerical precision of the exact route, input clipping and the constants needed to evaluate Equation (3). Every refit uses the published configuration, band selection and training seed; in a refit the initial weights are also drawn from a fixed seed, which the published run did not do (Section 2.8 of the article). Fidelity is the R^2 of the printed equation against the predictions of the network it was printed from, on the external test set; the largest difference is in the standardised output units in which the equation is written. Input values clipped: share of input values (bands × spectra) outside the training range and clipped to [−1, 1]. Training ranges are those of the SNV-corrected absorbance at each band on the training partition, which define the min–max scaling.

Quantity	Setting	Value
External R^2 of the network	Published network of Equation (3)	0.9841
External R^2 of the network	Refit, initialisation seeded (seed 42)	0.9837
External R^2 of the network	Refits, initialisation seeds 1–5	0.9834–0.9846
Fidelity	Published Equation (3), as printed (three significant figures; terms below 10^{-3} of the largest omitted)	$1 - 4.7 \times 10^{-7}$
Fidelity	Refit (seed 42), as printed	$1 - 3.3 \times 10^{-6}$
Fidelity	Refit (seed 42), printed at full double precision with nothing omitted	$1 - 1.3 \times 10^{-14}$
Fidelity	Refits (seeds 1–5), as printed	$1 - 1.5 \times 10^{-5}$ to $1 - 5.9 \times 10^{-7}$
Largest difference (standardised units)	Refit (seed 42), as printed	2.9×10^{-3}
Largest difference (standardised units)	Refit (seed 42), full double precision	5.8×10^{-7}
Terms in the printed equation	Published; refits (seeds 1–5)	31; 31–33
Bands retained	Published; refits (seeds 1–5)	8 of 8
Input values clipped	External test set	3.4%
Input values clipped	Withheld lot T1	0.8%
Input values clipped	Withheld lot T2	0.6%
Input values clipped	Withheld lot T3	0.2%
Input values clipped	Withheld lot T4	4.2%
Input values clipped	Withheld lot T5	1.0%
Training range of input	A_{1502} (SNV absorbance at 1502 cm^{-1})	−0.1891 to 0.0235
Training range of input	A_{1487} (SNV absorbance at 1487 cm^{-1})	−0.2284 to −0.0748
Training range of input	A_{1447} (SNV absorbance at 1447 cm^{-1})	0.9699 to 1.0659
Training range of input	A_{1286} (SNV absorbance at 1286 cm^{-1})	0.6717 to 0.7258
Training range of input	A_{1242} (SNV absorbance at 1242 cm^{-1})	1.4729 to 1.6054
Training range of input	A_{1180} (SNV absorbance at 1180 cm^{-1})	2.3884 to 2.5875
Training range of input	A_{1140} (SNV absorbance at 1140 cm^{-1})	3.0116 to 3.1591
Training range of input	A_{989} (SNV absorbance at 989 cm^{-1})	0.6277 to 0.9986
Output scaling	Tahini fraction (%): training mean and SD	76.16 and 32.73

https://doi.org/10.3390/foods15203639

References Cited Only in the Supplementary Materials

[62] Jackson, S.W.; Jang, M.H.; Asani, E.; Yates, C.R.; Ng, J.; Baudry, J. Infrared spectroscopic characterization of sesamin, a dietary lignan natural product. *PLoS ONE* **2024**, *19*, e0296541. https://doi.org/10.1371/journal.pone.0296541

[63] Moazzami, A.A.; Haese, S.L.; Kamal-Eldin, A. Lignan contents in sesame seeds and products. *Eur. J. Lipid Sci. Technol.* **2007**, *109*, 1022–1027. https://doi.org/10.1002/ejlt.200700057

[64] Górnaś, P.; Siger, A.; Pugajeva, I.; Segliņa, D. Sesamin and sesamolin as unexpected contaminants in various cold-pressed plant oils: NP-HPLC/FLD/DAD and RP-UPLC-ESI/MSn study. *Food Addit. Contam. Part A* **2014**, *31*, 567–573. https://doi.org/10.1080/19440049.2014.884285

[65] Allegrini, F.; Olivieri, A.C. IUPAC-consistent approach to the limit of detection in partial least-squares calibration. *Anal. Chem.* **2014**, *86*, 7858–7866. https://doi.org/10.1021/ac501786u

Disclaimer/Publisher's Note: The statements, opinions and data contained in all publications are solely those of the individual author(s) and contributor(s) and not of MDPI and/or the editor(s). MDPI and/or the editor(s) disclaim responsibility for any injury to people or property resulting from any ideas, methods, instructions or products referred to in the content.
